# Exploring the prevalence of gaming disorder and Internet gaming disorder: a rapid scoping review

**DOI:** 10.1186/s13643-020-01329-2

**Published:** 2020-04-02

**Authors:** Nazia Darvesh, Amruta Radhakrishnan, Chantelle C. Lachance, Vera Nincic, Jane P. Sharpe, Marco Ghassemi, Sharon E. Straus, Andrea C. Tricco

**Affiliations:** 1 Knowledge Translation Program, Li Ka Shing Knowledge Institute, St. Michael’s Hospital, Unity Health Toronto, 209 Victoria Street, East Building, Toronto, Ontario M5B 1W8 Canada; 2 Department of Medicine, University of Toronto, Toronto, Ontario Canada; 3 Epidemiology Division, Dalla Lana School of Public Health, University of Toronto, Toronto, Ontario Canada

**Keywords:** Gaming disorder, Internet gaming disorder, DSM-5, ICD-11, Prevalence, Rapid review, Scoping review, Knowledge synthesis

## Abstract

**Background:**

Internet gaming disorder (IGD) was included in the DSM-5 in 2013 as a condition requiring further research, and gaming disorder (GD) was included in the ICD-11 in 2018. Given the importance of including these conditions in diagnostic guidelines, a review was conducted to describe their prevalence.

**Methods:**

Using guidance from the Joanna Briggs Institute and the Preferred Reporting Items for Systematic reviews and Meta-Analyses extension for Scoping Reviews (PRISMA-ScR), we conducted a rapid scoping review. MEDLINE, Embase, PsycINFO, and the Cochrane library were searched for literature published from inception to July 2018. All review stages were pilot-tested to calibrate reviewers. The titles/abstracts and full-text articles were screened by one reviewer to include quantitative primary studies that reported GD or IGD prevalence. Excluded citations were screened by a second reviewer to confirm exclusion. Charting was conducted by one reviewer and verified by another, to capture relevant data. Results were summarized descriptively in tables or text.

**Results:**

We assessed 5550 potentially relevant citations. No studies on GD were identified. We found 160 studies of various designs that used 35 different methods to diagnose IGD. The prevalence of IGD ranged from 0.21–57.50% in general populations, 3.20–91.00% in clinical populations, and 50.42–79.25% in populations undergoing intervention (severe cases). Most studies were conducted in the Republic of Korea (*n* = 45), China (*n* = 29), and the USA (*n* = 20). Results are also presented for severe IGD and by geographic region, gender/sex, and age groups (child, adolescent, adult). The five most frequently reported health-related variables were depression (67 times), Internet addiction (54 times), anxiety (48 times), impulsiveness (37 times), and attention-deficit hyperactivity disorder (24 times).

**Conclusions:**

Due to the variability in diagnostic approaches, knowledge users should interpret the wide IGD prevalence ranges with caution. In addition to further research on GD, consensus on the definition of IGD and how it is measured is needed, to better understand the prevalence of these conditions.

## Background

Internet gaming disorder (IGD) was included as a non-substance addiction in the appendix of the fifth edition of the Diagnostic and Statistical Manual of Mental Disorders (DSM-5) published in 2013 by the American Psychiatric Association [1]. The need to add IGD to this diagnostic manual was identified by an international expert working group that reviewed over 250 articles [2], some of which showed the detrimental effects of excessive gaming [2]. As part of the DSM-5 approach to defining IGD, draft diagnostic criteria were proposed with some similarities to substance use disorders and added to the appendix of the DSM-5 manual, suggesting IGD was a condition warranting further research [2]. Nine criteria for IGD were recommended in the DSM-5: (1) high pre-occupation with gaming, (2) withdrawal symptoms, (3) increased tolerance to gaming, (4) unsuccessful attempts to stop or reduce gaming, (5) loss of interest in other hobbies or activities, (6) excessive gaming despite negative consequences, (7) deception about gaming activities towards others, (8) use of gaming as escape or relief from a negative mood, and (9) jeopardized or lost relationships, jobs, or educational or career opportunities [2].

The harmful consequences of excessive gaming were also recognized by the World Health Organization (WHO), and gaming disorder (GD) was included in their 2018 release of the 11th revision of the International Classification of Diseases (ICD-11) [3, 4]. According to the WHO, diagnostic criteria for GD are needed, in light of public health and treatment strategies that have been implemented worldwide to address the condition [3, 4]. In the ICD-11 definition, GD is categorized as a disorder due to addictive behaviors, and is “a pattern of persistent or recurrent gaming behavior (“digital gaming” or “video-gaming”), which may be online (i.e., over the Internet) or offline, and manifested by the following three criteria: (1) impaired control over gaming (e.g., onset, frequency, intensity, duration, termination, context), (2) increasing priority given to gaming to the extent that gaming takes precedence over other life interests and daily activities, and (3) continuation or escalation of gaming despite the occurrence of negative consequences. For gaming disorder to be diagnosed, the behavior pattern must be of sufficient severity to result in significant impairment in personal, family, social, educational, occupational, or other important areas of functioning. The pattern of gaming behavior may be continuous or episodic and recurrent. The gaming behavior and other features are normally evident over a period of at least 12 months in order for a diagnosis to be assigned, although the required duration may be shortened if all diagnostic requirements are met and symptoms are severe” [3, 4].

Given the emerging importance of GD and IGD as indicated by additions to the ICD-11 and DSM-5 diagnostic classification systems, a summary of the prevalence of these two conditions is needed. A rapid scoping review was commissioned by the WHO to describe the prevalence of GD and IGD in the literature, for all populations globally, using ICD-11 and DSM-5-specific criteria, respectively.

## Objectives

To conduct a rapid scoping review to describe the following:

1. The prevalence of (i) GD defined by the ICD-11 and (ii) IGD defined by the DSM-5, in people of all ages across all geographic areas.
2. The prevalence of severe cases of (i) GD and (ii) IGD, in people of all ages across all geographic areas. Severe cases were defined as those undergoing intervention.
3. Variables such as mental health outcomes that researchers in the field measure for persons of all ages across all geographic areas who have (i) GD or (ii) IGD.

## Methods

We used the Joanna Briggs Institute (JBI) methodological guidance for scoping reviews to inform our scoping review methods [5]. The Preferred Reporting Items for Systematic reviews and Meta-Analyses extension for Scoping Reviews (PRISMA-ScR) [6] was used to guide the reporting of this rapid scoping review, and the checklist is provided in Additional file 1. At the time of conducting this review, a PRISMA extension to rapid reviews was under development.

### Protocol and registration

The protocol for this rapid scoping review was developed a priori and registered on the Open Science Framework [7] on August 21, 2018 [8].

### Eligibility criteria

The eligibility criteria were developed using the PECOS components, as follows:

#### P—Population

We included individuals of all ages across all geographic areas to explore GD and IGD in all populations.

#### E—Exposures

We included populations that had ICD-11-defined GD [4] or DSM-5-defined IGD [1], according to our objectives. All types of gaming behaviors (e.g., online/offline, mobile/console/computer, single/multi-player) were relevant for our review. For an IGD diagnosis, five or more of the nine criteria needed to be met [2]. As this was a scoping review, we included all studies that applied this definition, so that we could chart all of the various methods and diagnostic tests that have been reported in the literature.

We excluded studies with populations that were defined using adapted criteria from conditions in older versions of the DSM (e.g., DSM-IV) as these did not fit the specific definitions of interest for our review. We also excluded studies with populations who only had gambling conditions or disorders related to gambling games (e.g., casino or poker games) as gambling-related conditions were considered separate from GD or IGD and outside of the scope of this review.

#### C—Comparators

In order to be inclusive of any study that reported prevalence, we included studies with any type of comparator or studies without a comparator.

#### O—Outcomes

We included studies that reported the prevalence of GD or IGD, according to our objectives. Prevalence is defined as a measure of the occurrence of the health condition or exposure of interest; it is the total number of individuals who have the condition (cases) at a particular time (or during a particular period) divided by the number of persons in the population at a specified time [9]. To capture the various methods of reporting GD or IGD in the literature, studies that reported GD or IGD scores on diagnostic scales were also included.

We included studies that reported the prevalence or scores of severe cases of GD or IGD. Severe GD or IGD was defined as cases where an entire population with GD or IGD was undergoing treatment for GD or IGD.

#### S—Study designs

We included primary quantitative studies such as randomized controlled trials (RCTs), controlled before-after studies, uncontrolled before-after studies, controlled after studies, quasi-experimental studies, observational studies, case-control studies, and cross-sectional studies. Studies that were categorized as observational [9] were non-experimental studies where there was not enough information from the article to determine the specific study design.

We included mixed methods studies if they were qualitative studies that reported quantitative data on our outcomes of interest; otherwise, qualitative studies were excluded. We also excluded books, case studies, case series, and reviews in our rapid review; however, a list of potentially relevant reviews is provided in Appendix A (Additional file 2).

#### Year of publication, publication status, and language

We included full-text publications from all years of publication. Gray literature (i.e., difficult to locate or unpublished material) in the form of theses/dissertations and conference abstracts was included if a full-text article was available. We limited the included studies to those in the English language, due to the short timeline to conduct the review; however, a list of potentially relevant non-English studies is provided in Appendix B (Additional file 2).

### Literature search strategy

The literature search strategy was drafted by an experienced librarian (BS) with input from the research team, and peer-reviewed by a second experienced librarian (HM) using the Peer Review of Electronic Search Strategies (PRESS) checklist [10]. An experienced library technician (AE) searched MEDLINE, EMBASE, PsycINFO, and the Cochrane Library on July 9, 2018, exported the literature search results into EndNote and discarded duplicates. To ensure saturation, we also assessed studies that were identified by content experts. The final literature search strategy for MEDLINE, EMBASE, and PsycINFO is found in Appendix C (Additional file 2).

### Selection of sources of evidence

A standardized screening form to determine the eligibility of studies for title and abstract screening was developed and pilot-tested independently by team members on the same 25 citations in Synthesi.SR, proprietary software developed by the Knowledge Translation Program of St. Michael’s Hospital [11]. After one pilot, ≥ 80% inter-rater agreement was reached, whereby the include/exclude status of at least 80% of the studies was unanimously agreed upon by the entire team. The standardized form was modified as needed, and the remaining titles and abstracts were screened by one reviewer (VN, JPS, MG, AD, SL, NT). A second reviewer verified excluded citations (ND, CCL, VN, JPS, MG, RC).

A standardized screening form to determine the eligibility of full-text articles was developed and pilot-tested independently by team members on the same 25 citations in Synthesi.SR [11]. After two pilots of 25 studies each (50 in total), ≥ 80% inter-rater agreement was reached, whereby the include/exclude status of at least 80% of the studies was unanimously agreed upon by the entire team. The standardized form was modified as needed and each remaining full-text article was screened by one reviewer (ND, AR, RC, VN, JPS, MG). A second reviewer verified excluded articles (ND, AR, VN, JPS, MG).

### Data charting process and data items

A standardized charting form was pilot-tested independently by team members on a sample of five studies in Excel. After two pilots of five studies each (10 in total), sufficient agreement was reached. The standardized form was modified, as required, in an iterative process [6]. Each study was charted by one reviewer (VN, JPS, MG, RB) and verified by a second (ND, AR). Due to the time limitations of this rapid scoping review, the authors of the included studies were not consulted to confirm data. We extracted the following data for general, clinical, and severe populations: country, study design, population description, sample size, age, gender/sex proportions, gaming time outcomes, GD or IGD reporting method (self-report, health professional, etc.), GD or IGD measure/instrument/assessment, GD or IGD prevalence numerator/denominator (number of people with GD or IGD, divided by total population at risk), GD or IGD prevalence estimate and 95% confidence interval (CI), GD or IGD mean score estimate and standard deviation (SD), and the list of variables (e.g., mental health outcomes) reported.

We charted data for three groups as follows:

- General: Populations that were not seeking treatment for GD or IGD, did not have GD or IGD at recruitment, and were not undergoing treatment for GD or IGD
- Clinical: Populations who sought treatment and populations with GD or IGD at recruitment
- Severe: Populations undergoing treatment for GD or IGD. Severity could have been reported by the authors of included studies in the following four ways: (i) authors distinguished between GD/IGD and severe GD/IGD using a test/scale to categorize participants into a separate severe group, (ii) a portion of the population was undergoing treatment for GD or IGD, (iii) the entire population with GD or IGD was undergoing treatment for GD or IGD, and (iv) a portion of the population sought treatment for GD or IGD.

For objectives 1 and 2, we charted prevalence results that were defined by having met at least five IGD criteria. In one case where studies separated outcomes based on whether or not participants answered “somewhat agree” versus “strongly agree” on a polytomous scale [12], we charted the outcomes where a response of “strongly agree” was indicative of a criterion being endorsed. For objective 3, we charted all reported variables only in the included studies that reported prevalence or scores for GD or IGD. We considered variables to be all demographic data that study authors reported (e.g., education, income) and all outcomes of interest to study authors (e.g., depression, brain imaging characteristics).

For studies where multiple prevalences or score results were presented, we charted the results from the main analysis (or from a secondary analysis if the main analysis results were not reported as prevalence or scores). Since most studies reported only on gender or only on sex and did not clarify which of these two were measured and how, we charted gender or sex as reported in the article and included it as one data item. We reported the proportions of gender/sex as presented by authors; if authors did not report data for a particular gender/sex category, we did not chart any data for that category. Gender/sex proportions were reported as percentages rounded to the nearest whole number. In cases where authors of included studies only provided raw data for prevalence or gender/sex proportions, we calculated these so that they would be reported consistently across studies.

### Risk of bias appraisal of individual sources of evidence

We did not appraise the risk of bias of individual sources of evidence, which is consistent with established scoping review methods [5, 6].

### Quantitative analyses

We did not conduct summary quantitative analyses (e.g., meta-analyses) of prevalence data, which is consistent with established scoping review methods [5, 6].

### Synthesis of results

To synthesize the rapid scoping review findings, we provide the prevalence or score estimates for GD, IGD, severe GD, and severe IGD for each study in a tabular format and summarize the ranges of prevalence estimates in the text. We do not provide ranges for mean score data (between test comparison), as the tests differed widely and their numerical values could not be compared and summarized together. We also do not provide the ranges of prevalence or mean scores by each of the measures (within test comparison), as most of the tests had results from only one included study, the populations in these included studies were different, and the results may have been on different scales. In addition, we provide a summary of the frequencies of variables reported in the included studies. Where appropriate, we summarize findings by the following subgroups: WHO geographic region [13], gender/sex categories, and age groups. Age groups were defined as follows: children (0–19 years old), adolescents (10–19 years old), and adults (18 years and older) [14]. All adolescent-specific data were also included in the children’s group. WHO geographic regions [13] included the following: African Region, Eastern Mediterranean Region, European Region, Region of the Americas, South-East Asia Region, and the Western Pacific Region. In addition, based on the included studies, we created a map using the Mapchart tool [15] to show the number of articles found and prevalence ranges by WHO regions.

### Deviations from the study protocol

Due to time and resource constraints of this rapid scoping review (the WHO requested results within 5 months), we were unable to scan the reference lists of included studies and relevant systematic reviews or comprehensively chart quantitative comorbidity results as part of our third objective. Instead, we charted all variables that were reported by study authors in included studies, to describe the variables that researchers in the field of IGD and GD measure when studying these populations.

## Results

### Selection of sources of evidence

**Fig. 1:**
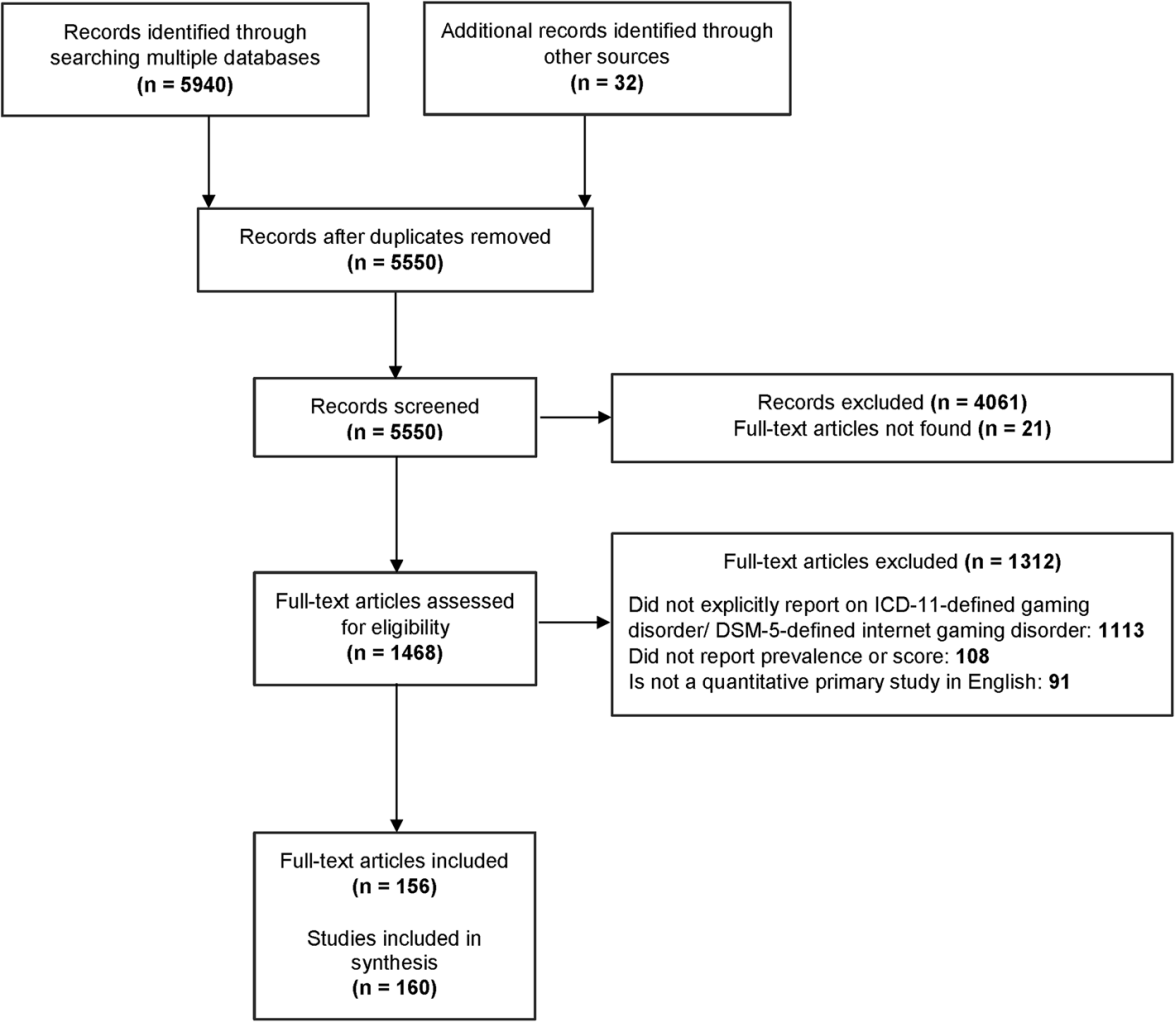
Study flow. Study flow of the review

Figure 1 shows the study flow, and Appendix D (Additional file 2) provides the list of included full-text articles. After removing duplicates, the titles and abstracts of 5550 citations were screened for eligibility. Of the 1489 relevant citations, we retrieved the full-text articles for 1468 citations (21 citations did not have accessible full-texts). From the 1468 full-text articles screened, 1113 were excluded because they did not explicitly report on ICD-11-defined GD or DSM-5-defined IGD, 108 were excluded for not reporting prevalence or score data, and 91 were not quantitative primary studies in English. We found 156 full-text articles published between 2014 and 2018 that represented 160 studies (some full-text articles reported results from multiple studies). Accounting for duplicate studies on the same population, our review identified 137 unique studies and 23 companion reports.

### Study characteristics

We did not identify any studies that reported prevalence or score data for GD. There were 35 different methods reported in the literature to measure IGD. Table 1 shows each of these methods, the range of values for each, and the number of studies in our review that reported that method.

**Table 1:** Methods used to identify people with Internet gaming disorder

Method		Range‡	No. of studies
1	C-IGDS	0–9*	1
2	C-VAT 2.0	NA	1
3	DQVMIA	NR	1
4	DSM-5 criteria for IGD	0–9	48
5	DSM-5 questionnaire - German	0–9*	1
6	GAIT	0–52	1
7	Health professional applying the DSM-5 IGD criteria	NA	46
8	IGD Checklist - 12 items	0–9*	3
9	IGD Checklist - 9 items	0–9*	5
10	IGD Scale - 27 item dichotomous	0–27	1
11	IGD Scale - 27 item polytomous	0–135*	2
12	IGD Scale - 27 item polytomous – Turkish	0–135*	1
13	IGD Scale - 9 item	0–9	2
14	IGD Scale - 9 item dichotomous	0–9	9
15	IGD Scale - 9 item polytomous - Turkish	0–45*	1
16	IGD Scale - dichotomous	0–27	1
17	IGD Scale - polytomous	0–135*	2
18	IGD-20 Test	20 –100*	1
19	IGD-20 Test – Spanish	20–100*	1
20	IGD-9 Scale	0–9	1
21	IGDI	NA	1
22	IGDQ – German	0–9*	2
23	IGDS9-SF	9–45	16
24	IGDS9-SF – Italian	9–45*	3
25	IGDT-10	0–9	3
26	Internet Gaming Addiction Scale	0–7*	1
27	K-scale - Korean Internet Addiction Scale for Adolescents	20––80	1
28	PIE-9	9–45*	1
29	Problem gaming instrument	0–11*	1
30	PVP Scale	0–9	4
31	SCI-IGD	NA	1
32	VAT	0–56*	1
33	VGA questionnaire (revised)	15–45*	1
34	VGAQ	9–45*	1
35	Video Game Dependency Scale	18–72*	1

*NA* not applicable (the method is not based on a numerical score); *NR* not reported (information not provided in the included study); *C-IGDS* Chinese Internet Gaming Disorder Scale; *C-VAT* Clinical Video game Addiction Test; *DQVMIA*: Diagnostic Questionnaires for Video Games, Mobile Phone or Internet Addiction; *GAIT* The Gaming Addiction Identification Test; *IGD* Internet Gaming Disorder; *IGDI* Internet Gaming Disorder Interview; *IGDQ* Internet Gaming Disorder Questionnaire; *IGDS9-SF* Internet Gaming Disorder Scale - Short Form; *IGDT-10* Ten-Item Internet Gaming Disorder Test; *PIE-9* Personal Internet Gaming Disorder Evaluation; *PVP Scale* Problematic Videogame Playing Scale; *SCI-IGD* Structured Clinical Interview for Internet Gaming Disorder; *VAT* Video game Addiction Test; *VGA* Video Game Addiction; *VGAQ* Video Game Addiction Questionnaire

‡Lower to upper limit of responses

*Range was calculated using reported data

Scales varied in terms of the number of questions and response options. The most common methods to diagnose IGD were the general application of the DSM-5 criteria, a health professional application of the DSM-5 criteria, and the Internet Gaming Disorder Scale-Short-Form (IGDS9-SF) [16].

**Fig. 2:**
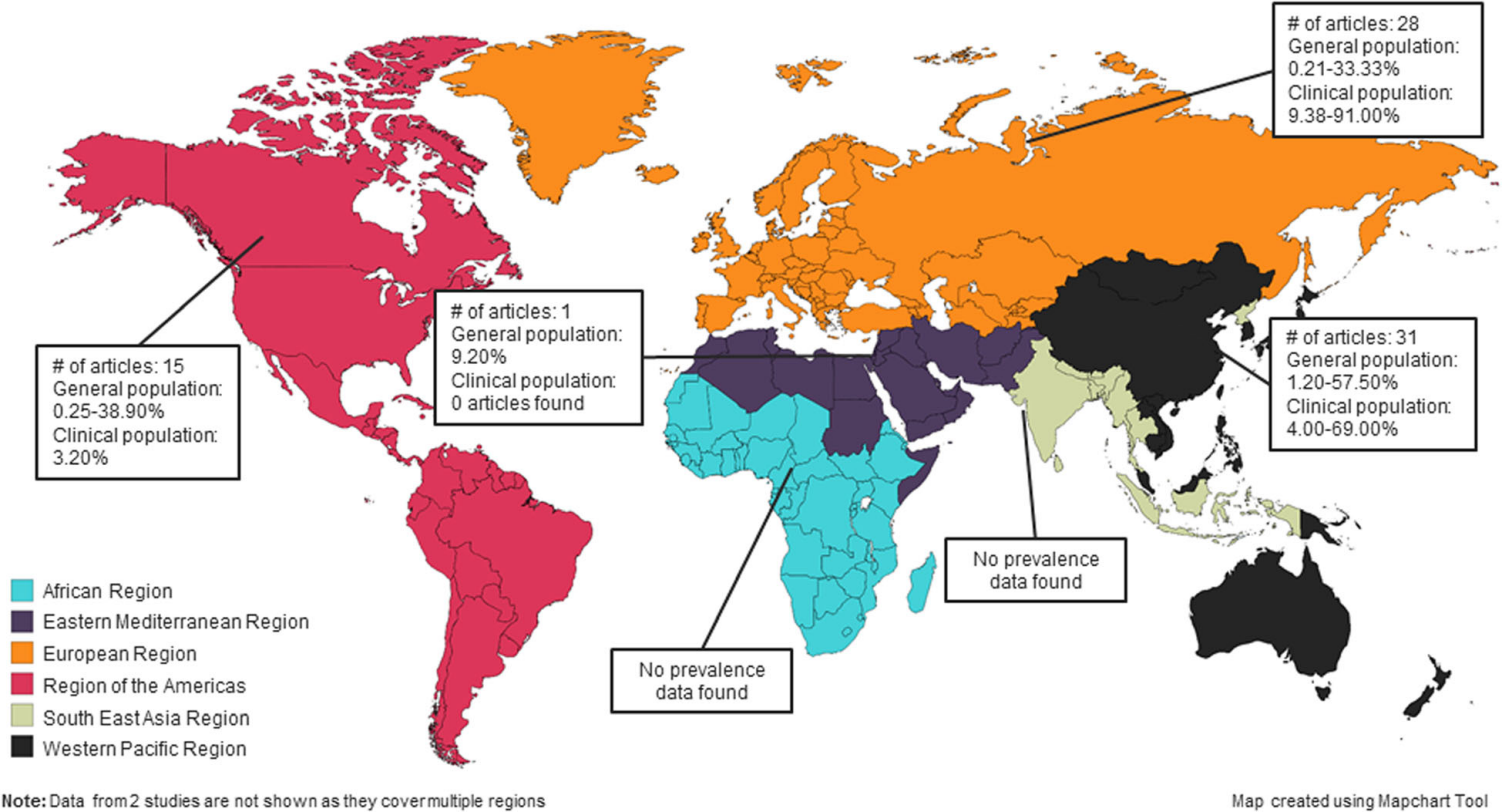
Prevalence of internet gaming disorder by WHO region. Prevalence of internet gaming disorder in the African Region, Eastern Mediterranean Region, European Region, Region of the Americas, South-East Asia Region, and Western Pacific Region

Appendix E (Additional file 2) shows characteristics for studies that reported IGD prevalence or mean score data for general or clinical populations by WHO geographic region. Figure 2 shows the prevalence ranges on a world map [15]. Appendix F (Additional file 2) shows characteristics for studies that reported prevalence or mean scores for general or clinical populations by gender/sex groups. IGD prevalence was reported in males and females and not for other gender/sex categories.

Appendix G (Additional file 2) shows characteristics for studies that reported prevalence or mean scores by age groups. Appendix H (Additional file 2) shows characteristics for studies with severe cases of IGD by WHO geographic region.

Of a total of 160 studies in our review, there were 99 cross-sectional studies, 38 observational studies, six RCTs, six controlled before-after studies, five uncontrolled before-after studies, four case-control studies, one controlled after study, and one quasi-experimental study. Twenty-six percent of studies (*n* = 42) were conducted in male-only populations. The included studies were conducted across 28 countries, the majority in the Republic of Korea (*n* = 45), China (*n* = 29), the USA (USA) (*n* = 20), Australia (*n* = 14), and in Germany (*n* = 13). Twenty-four studies reported prevalence data for children and of these, 21 reported prevalence data specifically on adolescents. Thirty-five studies reported prevalence data on adult populations. Fourteen studies had combined prevalence data for children and adults, and five studies did not describe the ages of the study population. IGD outcomes were self-reported or parent-reported in 91 studies and diagnosed by a health professional in 50 studies.

### Prevalence of Internet gaming disorder in general, clinical, and severe populations

Table 2 shows the ranges of prevalence from included studies by general, clinical, and severe populations.

**Table 2:** Prevalence of Internet gaming disorder by population type

	General populations: prevalence range (%) [# of studies]	Clinical populations: prevalence range (%) [# of studies]	Undergoing intervention (severe): prevalence range (%) [# of studies]
***WHO region***			
African	*No studies found*	*No studies found*	*No studies found*
Eastern Mediterranean	9.20 [1]	*No studies found*	*No studies found*
European	0.21–33.33 [25]	9.38–91.00 [2]	68.60 [1]
Region of the Americas	0.25–38.90 [11]	3.20 [1]	76.60 [3]
South-East Asia	*No studies found*	*No studies found*	*No studies found*
Western Pacific	1.20–57.50 [22]	4.00–69.00 [7]	50.42–79.25 [2]
Multiple regions	0.56–5.28 [2]*	*No studies found*	*No studies found*
***Gender/sex***			
Male	0.21–57.50 [25]	33.91–91.00 [3]	50.42–79.25 [2]
Female	0.25–26.09 [21]	69.00 [1]	*No studies found*
***Age groups***			
Children (0–19 years old)	0.26–38.00 [20]	7.93–11.44 [2]	68.60–79.25 [2]
Adolescents (10–19 years old)	0.26–38.00 [17]	7.93–11.44 [2]	68.60–79.25 [2]
Adults (18 years and older)	0.21–55.77 [27]	3.20–69.00 [6]	76.60 [3]

General: Populations that were not seeking treatment for GD or IGD, did not have GD or IGD at recruitment, and were not undergoing treatment for GD or IGD. Clinical: Populations who sought treatment, and populations with GD or IGD at recruitment. Severe: Populations undergoing treatment for GD or IGD. *WHO* World Health Organization

^#^Number

*Studies covered multiple geographic regions

Of 61 studies that reported prevalence data in a general population, the prevalence of IGD ranged from 0.21–57.50%. Of 10 studies that reported prevalence data in clinical populations, the prevalence of IGD ranged from 3.20–91.00%. In six studies that reported IGD prevalence data for severe cases, the prevalence ranged from 50.42–79.25%. Although all individuals in these severe populations were undergoing treatment for IGD, prevalence ranges were sometimes less than 100%. This is because some studies were conducted to compare different IGD diagnostic methods, and those methods did not always identify 100% of the population as severe.

### Variables reported in populations with Internet gaming disorder

Appendix I (Additional file 2) shows the frequencies of reported variables for the following categories: demographic characteristics, drug-related variables, game-related variables, mental health and well-being, physical and physiological characteristics, and relationship-related variables. In the 108 articles that reported variables, the five most frequently reported health conditions were depression/depressiveness (67 times), Internet addiction (54 times), anxiety (47 times), impulsiveness/impulsivity (37 times), and attention-deficit hyperactivity disorder/attention deficit disorder (23 times). The five most frequently reported non-health conditions were gaming time (e.g., hours gaming per day, duration of gaming sessions) (69 times), education-related outcomes (e.g., education level, years of education, grade point average) (41 times), task-related outcomes (e.g., decision-making during a task, reaction time during a task) (36 times), gaming context outcomes (e.g., favorite gaming genres, type of gaming device) (32 times), and brain imaging characteristics (e.g., brain scans) (28 times).

## Discussion

### Summary of evidence

We conducted a rapid scoping review to describe the prevalence of GD, IGD, severe GD, and severe IGD, in people of all ages, across all geographic areas, and to describe the variables reported in included studies. The majority of studies were conducted in the Western Pacific region [13]. No studies reported GD prevalence which is expected given that it was added to the ICD-11 in 2018. We found 35 different methods that were used to diagnose IGD. The prevalence of IGD ranged from 0.21–57.50% in general populations, 3.20–91.00% in clinical populations, and 50.42–79.25% in populations undergoing interventions (i.e., severe cases). Since different tests were used to diagnose IGD without standardization across studies, the IGD prevalence ranges should be interpreted with caution. The variability in how IGD was measured is indicative of the debate surrounding this topic and lack of consensus regarding diagnostic criteria [17]. To ensure that our reported summary measures were representative of DSM-5-specific criteria, we only included studies in which IGD was diagnosed using five or more of these nine criteria. However, included studies that applied DSM-5 criteria used a number of additional terms to define IGD such as gaming addiction and problematic gaming and applied the DSM-5 criteria in 35 different ways. The wide ranges of IGD prevalence in this review may thus be a result of the various methods that were used to assess IGD.

Previous reviews on gaming-related conditions such as IGD, problematic gaming behavior, and Internet addiction that were found in our search reported a wide range of prevalence estimates. For example, a 2017 systematic review by Mihara et al. that focussed only on cross-sectional and longitudinal studies measuring IGD, reported a global prevalence ranging from 0.70–27.50% [18]. A review by Feng et al*.* published in 2017 [19] reported an IGD prevalence range of 0.70–15.60%. Finally, another recent systematic review published in 2018 by Paulus et al*.* reported a wider prevalence range of IGD that was between 0.60–50.00% [20]. Unlike our review which focussed on DSM-5-defined IGD only, these reviews included studies that diagnosed IGD based on a variety of definitions such as DSM-III R criteria for pathological gambling, DSM-IV criteria for substance dependence, and the Internet Addiction Test. The results from these reviews demonstrate that prevalence estimates in the literature vary considerably. The differences in prevalence are likely due to the populations included or the tools used to diagnose IGD. For instance, in these previously published reviews, primary studies used Young’s Internet Addiction Test to diagnose IGD, to diagnose severe IGD, or to diagnose internet addiction, which is a broad term that includes online addictions that are beyond the scope of gaming; as such, the prevalence of IGD in studies using these criteria would likely differ compared to those that use the more restrictive DSM-5 criteria. Consensus by experts in the field on the definition and scope of IGD and how it should be measured may result in more accurate and precise prevalence ranges in both primary studies and in reviews.

A report of the results of this review was provided to the WHO. A variety of knowledge users can use the results from this review to understand the body of evidence available on GD and IGD. In particular, mental health and public health clinicians, funders, researchers, and policy makers can use the information to understand the various ways that IGD is currently measured in research studies, and the ranges of IGD prevalence by different population types, geographic regions, and age groups.

### Limitations

Our review focused on ICD-11-defined GD and DSM-5-defined IGD only and therefore does not include studies about gaming conditions that do not use these definitions. There are varying definitions of excessive gaming and gaming addiction discussed in the literature [17, 21]. Furthermore, GD was recently added to the ICD-11 in 2018, studies applying these new criteria may not be published yet, and IGD appears in the appendix of the DSM-5, implying that its inclusion as an official disorder remains under discussion.

Our rapid scoping review was also limited to the English language, articles found in major databases, and those provided by content experts; literature published in non-English languages was not captured. Given that there are treatment centers addressing gaming-related issues in countries such as China, India, Japan, and the Republic of Korea, there may be other prevalence estimates in the literature in non-English languages that did not contribute to our results. Although our included studies are English-only, we did not limit our search to the English language and a list of potentially relevant non-English studies is provided in Appendix B (Additional file 2).

We were unable to provide IGD mean score ranges because they were measured on different scales; however, mean score data for individual studies is available in Additional file 2.

We were unable to scan the reference lists of included articles and systematic reviews or describe the prevalence of comorbidities quantitatively as stated in our protocol due to time and resource constraints; however, we provide a list of included full-text articles in Appendix D (Additional file 2), a list of potentially relevant reviews in Appendix A (Additional file 2), and the frequencies of variables in Appendix I (Additional file 2).

### Future considerations

Since previous reviews have been conducted using a variety of definitions for gaming-related conditions and have found studies with different diagnostic measures for these conditions, a consensus on the diagnostic criteria for IGD must be reached, so it can be recognized clinically in a standardized way and so that research on the condition can be summarized appropriately. Given that there were 35 measures for IGD, the validity of different tools should also be assessed [21]. Furthermore, given the recent addition of GD to ICD-11, future reviews on GD must be conducted to provide a range for GD prevalence. Future reviews could conduct a quality appraisal of included studies and quantitative summary analyses to comment on the quality of the evidence and appropriately compare whether the prevalence ranges in the literature are statistically and meaningfully different for different population types (e.g., by clinical groups, by those undergoing intervention, by different geographic areas, by gender/sex classifications). In addition, further quantitative analyses could compare the mean scores for IGD. Finally, future reviews should include studies published in languages other than English.

## Conclusions

We found 160 studies that used 35 different methods to diagnose IGD, with prevalence ranging from 0.21–57.50% in general populations, 3.20–91.00% in clinical populations, and 50.42–79.25% in populations undergoing interventions (severe cases). Knowledge users from a variety of fields can use these results to understand the current evidence on IGD prevalence.

Because of the variety of diagnostic methods found in this review, the reported IGD prevalence ranges should be interpreted with caution. A consensus on the definition of IGD and how it is measured needs to be reached so that its prevalence can be more accurately estimated. Primary studies need to be conducted to estimate GD prevalence, followed by a future review to summarize the ranges.

## Supplementary information

**Additional file 1.** PRISMA-ScR Checklist.

**Additional file 2.** Appendices A-I.

## Data Availability

All datasets supporting the conclusions of this article are included within the article and its additional files.

## Abbreviations

CI: Confidence interval
DSM-5: Diagnostic and Statistical Manual of Mental Disorders, 5th edition
GD: Gaming disorder
ICD-11: International Classification of Diseases, 11th revision
IGD: Internet gaming disorder
IGDS9-SF: Internet Gaming Disorder Scale–Short-Form
JBI: Joanna Briggs Institute
PRESS: Peer Review of Electronic Search Strategies
PRISMA-ScR: Preferred Reporting Items for Systematic reviews and Meta-Analyses extension for Scoping Reviews
RCT: Randomized controlled trial
SD: Standard deviation
WHO: World Health Organization
