# Supplementary material for: Exploring the prevalence of gaming disorder and Internet gaming disorder: a rapid scoping review
Source: Syst Rev. 2020 Apr 2;9:68. doi: 10.1186/s13643-020-01329-2 (PMC7119162; doi:10.1186/s13643-020-01329-2)
Supplement: Supplementary file 2 — Additional file 2. Appendices A-I. [file 13643_2020_1329_MOESM2_ESM.pdf]

Additional File 2

Table of Contents

Appendix A. List of potentially relevant reviews 2

Appendix B. List of potentially relevant non-English studies 3

Appendix C. Search strategy (MEDLINE, Embase, PsycINFO) 8

Appendix D. List of included full-text articles 11

Appendix E. Study and population characteristics for studies with general or clinical populations, by WHO region 21

Appendix F. Study and population characteristics for studies with general or clinical populations, by gender/sex 38

Appendix G. Study and population characteristics for studies with general or clinical populations, by age groups 54

Appendix H. Study and population characteristics for studies with populations undergoing an intervention (severe) 71

Appendix I. Frequency of variables reported in populations with internet gaming disorder 74

References 78

Appendix A. List of potentially relevant reviews

1. González-Bueso V, Santamaría JJ, Fernández D, Merino L, Montero E, Ribas J. Association between Internet Gaming Disorder or Pathological Video-Game Use and Comorbid Psychopathology: A Comprehensive Review. *Int J Environ Res Public Health.* 2018;15(4):668.
2. Mihara S, Higuchi S. Cross-sectional and longitudinal epidemiological studies of Internet gaming disorder: A systematic review of the literature. *Psychiatry Clin Neurosci.* 2017;71(7):425-444.
3. Paulus FW, Ohmann S, von Gontard A, Popow C. Internet gaming disorder in children and adolescents: a systematic review. *Dev Med Child Neurol.* 2018;60(7):645-659.
4. Feng W, Ramo DE, Chan SR, Bourgeois JA. Internet gaming disorder: Trends in prevalence 1998-2016. *Addict Behav.* 2017;75:17-24.

Appendix B. List of potentially relevant non-English studies

1. Abreu CN, Karam RG, Goes DS, Spritzer DT. [Internet and videogame addiction: a review]. *Braz J Psychiatry.* 2008;30(2):156-167.
2. Aguilar MM, Vergara FA, Velasquez EJ, Garcia-Hermoso A. [Physical activity, screen time and sleep patterns in Chilean girls]. *An Pediatr (Barc).* 2015;83(5):304-310.
3. Allard C. Child and computer : Advantages and disadvantages. [French]. *Journal de Pediatrie et de Puericulture.* 2008;21(5-6):209-213.
4. Atlan J. [Protecting children and adolescents from the risks and excesses of the internet]. *Soins Pediatr Pueric.* 2012;Pediatrie, Puericulture.(265):29-31.
5. Batthyany D, Muller KW, Benker F, Wolfling K. [Computer game playing: clinical characteristics of dependence and abuse among adolescents]. *Wien Klin Wochenschr.* 2009;121(15-16):502-509.
6. Bertran E, Chamarro A. Video gamers of League of Legends: The role of passion in abusive use and in performance. *Adicciones.* 2016;28(1):28-34.
7. Beutel ME, Hoch C, Wolfling K, Muller KW. [Clinical characteristics of computer game and internet addiction in persons seeking treatment in an outpatient clinic for computer game addiction]. *Z Psychosom Med Psychother.* 2011;57(1):77-90.
8. Bialokoz-Kalinowska I, Kierus K, Nawrocka B, Piotrowska-Jastrzebska JD. Internet addiction (netaholics) in the secondary school adolescents - Impact on the health and psychosocial skills. [Polish]. *Pediatria i Medycyna Rodzinna.* 2011;7(4):372-377.
9. Bilic V, Golub TL. Pathological videogame use: the role of gender, self-esteem and educational context. *Hrvatska Revija Za Rehabilitacijska Istrazivanja.* 2011;47(2):1-13.
10. Bioulac S, Arfi L, Michel G, Bouvard MP. Interest of the use of the Problem Videogame Playing (PVP) questionnaire from Tejeiro: Exploratory study in ADHD children. [French]. *Ann Med Psychol (Paris).* 2010;168(8):632-635.
11. Boneberger A, Bolte G, von Kries R, Studiengruppe GME. [Excessive media comsumption and behavioural problems--a cross-sectional study on pre-school children]. *Gesundheitswesen.* 2011;73(5):280-285.
12. Bonnaire C, Phan O. [Negative perceptions of the risks associated with gaming in young adolescents: An exploratory study to help thinking about a prevention program]. *Arch Pediatr.* 2017;24(7):607-617.
13. Bonnaire C. Multidimensional Family Therapy: Risk and protective factors based on the case conceptualisation. Clinical illustration of its use in problematic gamers. *Therapie Familiale: Revue Internationale en Approche Systemique.* 2016;37(4):387-406.
14. Bronnec M, Rocher B, Bouju G, Venisse JL. Gaming, gambling and addiction. [French]. *Ann Med Psychol (Paris).* 2010;168(7):509-512.
15. Caillon J. [Prevention of excessive video gaming in school milieu]. *Rev Infirm.* 2010;(162):19.
16. Chamarro A, Carbonell X, Manresa JM, Munoz-Miralles R, Ortega-Gonzalez R, Lopez-Morron MR et al. [The Questionnaire of Experiences Associated with Video games (CERV): an instrument to detect the problematic use of video games in Spanish adolescents]. *Adicciones.* 2014;26(4):303-311.
17. Chirita V, Chirita R, Stefanescu C, Chele G, Ilinca M. Computer use and addiction in Romanian children and teenagers--an observational study. *Rev Med Chir Soc Med Nat Iasi.* 2006;110(3):526-532.
18. Choliz M, Marco C. Pattern of use and dependence on video games in infancy and adolescence. *Anales de Psicologia.* 2011;27(2):418-426.

19. Danet M, Miljkovitch R. The virtual world: Attachment-related issues and risks. *Psychologie Francaise.* 2017;62(1):57-83.
20. Dany L, Moreau L, Guillet C, Franchina C. Video Games, Internet and Social Networks: A Study among French School students. *Sante Publique.* 2016;28(5):569-579.
21. Dejoie JF. [Internet addiction: a different kind of addiction?]. *Rev Med Liege.* 2001;56(7):523-530.
22. Demirtas Madran A, Ferligul Cakilci E. The relationship between agression and online video game addiction: A study on massively multiplayer online video game players. *Anadolu Psikiyatri Derg.* 2014;15(2):99-107.
23. Dreier M, Wolfling K, Beutel ME. Internet addiction in youth. [German]. *Monatsschr Kinderheilkd.* 2014;162(6):496-502.
24. Du W, Liu J, Gao X, Li L, Li W, Li X et al. [Functional magnetic resonance imaging of brain of college students with internet addiction]. *Zhong Nan Da Xue Xue Bao Yi Xue Ban.* 2011;36(8):744-749.
25. Estevez Gutierrez A, Herrero Fernandez D, Sarabia Gonzalvo I, Jauregui Bilbao P. [Mediating role of emotional regulation between impulsive behavior in gambling, Internet and videogame abuse, and dysfunctional symptomatology in young adults and adolescents]. *Adicciones.* 2014;26(4):282-290.
26. Fang T-W. Relations among problematic internet use, coping, loneliness and online social support: A comparison of male and female undergraduates. *Bulletin of Educational Psychology.* 2010;41(4):773-797.
27. Ferchow S, Franke GH, Jagla M, Nowik D. Pathological use of online games - A survey of a collegiate sample. *Sucht.* 2015;61(2):95-102.
28. Fernandez-Villa T, Alguacil Ojeda J, Almaraz Gomez A, Cancela Carral JM, Delgado-Rodriguez M, Garcia-Martin M et al. Problematic Internet Use in University Students: associated factors and differences of gender. *Adicciones.* 2015;27(4):265-275.
29. Ferreira C, Ferreira H, Vieira MJ, Costeira M, Branco L, Dias A et al. [Epidemiology of Internet Use by an Adolescent Population and its Relation with Sleep Habits]. *Acta Med Port.* 2017;30(7-8):524-533.
30. Fleta Zaragozano J, Fleta Asin B. [Video games and the health of adolescent boys]. *Rev Enferm.* 1997;20(221):32, 35.
31. Frolich J, Lehmkuhl G, Dopfner M. [Computer games in childhood and adolescence: relations to addictive behavior, ADHD, and aggression]. *Z Kinder Jugendpsychiatr Psychother.* 2009;37(5):393-402; quiz 403-394.
32. Fuji K, Yoshida F. The effects of Internet behavior and attitudes on sociability and aggression: An examination of web-logs and online-games. *The Japanese Journal of Social Psychology.* 2009;25(2):121-132.
33. Gaetan S, Bonnet A, Pedinielli JL. [Self-perception and life satisfaction in video game addiction in young adolescents (11-14 years old)]. *Encephale.* 2012;38(6):512-518.
34. Gaetan S, Therme P, Bonnet A. The addictive use of video games is it an adaptive solution to the self-perception and depressive symptomatology in young adolescents (11-14 years)?. [French]. *Neuropsychiatr Enfance Adolesc.* 2015;63(4):250-257.
35. Grund J, Schulz W. [The Influence of Media Consumption During Early Childhood on Media Use and Psychological Disorders in Adolescence]. *Prax Kinderpsychol Kinderpsychiatr.* 2017;66(8):558-575.
36. Grusser SM, Thalemann R, Albrecht U, Thalemann CN. [Excessive computer usage in adolescents--results of a psychometric evaluation]. *Wien Klin Wochenschr.* 2005;117(5-6):188-195.
37. Gyepesi A, Elekes Z, Domokos T, Demetrovics Z. [Methodological background and descriptive features of the ESPAD 2011 Study (European School Survey Project on Alcohol and other Drugs) in Hungary]. *Psychiatr Hung.* 2013;28(1):30-38.

38. Hassler F, Reis O. [Attention deficit hyperactivity disorder (ADHD) and addiction]. *MMW Fortschr Med.* 2010;152(18):46-48.
39. Kardashian RA. [Individual psychological features in patients with computer game addiction suffering and predisposed persons]. *Zh Nevrol Psikhiatr Im S S Korsakova.* 2018;118(2):18-22.
40. Kim KS, Kim KH. [A prediction model for internet game addiction in adolescents: using a decision tree analysis]. *J Korean Acad Nurs.* 2010;40(3):378-388.
41. Koenig J, Fischer-Waldschmidt G, Brunner R, Resch F, Kaess M. [Refuge in Digital Worlds - the Association of Critical Life Events with Pathological Internet Use in Adolescence]. *Prax Kinderpsychol Kinderpsychiatr.* 2016;65(7):494-515.
42. Korkeila J. [Internet addiction]. *Duodecim.* 2012;128(7):741-748.
43. Kratzer S. [Pathological computer- and internet-use -"internet addiction". An increasing disorder in clinical practice]. *Fortschr Neurol Psychiatr.* 2011;79(4):242-249; quiz 250.
44. Kuss DJ, Griffiths MD. Internet video game addiction: A systematic review of empirical research in the literature. *Revue Adolescence.* 2012;30(1):17-49.
45. Lagercrantz H. [Too much time in front of the screen shatters children's lives. Obesity, concentration problems, impaired language development etc. may be the result]. *Lakartidningen.* 2013;110(1-2):16-17.
46. Le Heuzey MF, Mouren MC. [Videogame addiction: a danger for only at-risk children or for all children]. *Bull Acad Natl Med.* 2012;196(1):15-23; discussion 24-16.
47. Lehmkuhl G, Frolich J. [The new media and their influence on children and adolescents]. *Z Kinder Jugendpsychiatr Psychother.* 2013;41(2):83-86.
48. Lemos IL, Diniz PRB, Peres JFP, Sougey EB. Neuroimaging in video game addiction: A systematic review. *J Bras Psiquiatr.* 2014;63(1):57-71.
49. Lemos IL, Santana SM. Electronic games dependency: The possibility of a new psychiatric diagnosis. *Rev Psiquiatr Clín.* 2012;39(1):28-33.
50. Li Z-J, Li Y, Guo W. Pre-potent response inhibition functions under "cold" and "hot" representations in excessive internet use college students. *Chinese Mental Health Journal.* 2014;28(5):374-380.
51. Lijuan C, Hialang H, Minzheng W, Chunlin X. A Study on the Implicit Aggressiveness of Gaming Addicts. *Psychological Science (China).* 2006;29(3):570-573.
52. Lloret Irles D, Morell Gomis R, Marzo Campos JC, Tirado Gonzalez S. [Spanish validation of Game Addiction Scale for Adolescents (GASA)]. *Aten Primaria.* 2018;50(6):350-358.
53. Malischnig D. [Online gaming. Potential risk and prevention programs]. *Psychiatr Danub.* 2014;26(4):384-388.
54. Mathiak K. [Shooting games as leisure permit. Do computer games promote violence? (interview by Dr. Thomas Bisswanger-Heim)]. *MMW Fortschr Med.* 2006;148(1-2):15.
55. Mihara S, Osaki Y, Nakayama H, Sakuma H, Ikeda M, Itani O et al. Internet use and problematic Internet use among adolescents in Japan: A nationwide representative survey. *Addict Behav Rep.* 2016;4:58-64.
56. Mori A, Iwadate M, Minakawa NT, Kawashima S. [Game addiction]. *Nihon Rinsho.* 2015;73(9):1567-1573.
57. Mouren MC, Lejoyeux M, Le Heuzey MF. [On-screen games: from excess to addiction]. *Bull Acad Natl Med.* 2012;196(3):739-741.
58. Musalek M, Mechtcheriakov S, Yazdi K. [Non-drug addictions]. *Neuropsychiatrie.* 2014;28(4):218-221.
59. Nau JY. [The dangers of screen time among adolescents]. *Rev Med Suisse.* 2011;7(294):1062-1063.
60. Nickler C. [Video games, a therapeutic mediator for teens]. *Rev Infirm.* 2015;(214):35-36.

61. Nuyens F, Kuss DJ, Lopez-Fernandez O, Griffiths MD. The experimental analysis of problematic video gaming and cognitive skills: A systematic review. *Journal de Therapie Comportementale et Cognitive.* 2017;27(3):110-117.
62. Oliveira JS, Barufaldi LA, Abreu Gde A, Leal VS, Brunken GS, Vasconcelos SM et al. ERICA: use of screens and consumption of meals and snacks by Brazilian adolescents. *Rev Saude Publica.* 2016;50 Suppl 1:7s.
63. Paksi B, Demetrovics Z, Magi A, Felvinczi K. [The methodology and sample description of the National Survey on Addiction Problems in Hungary 2015 (NSAPH 2015)]. *Neuropsychopharmacol Hung.* 2017;19(2):55-85.
64. Pallas A, Meyer CH, Mojon D. [Nintendo 3DS: technology, physiology and possible risks for children's eyes]. *Ophthalmologe.* 2013;110(3):263-266.
65. Pallesen S, Molde H, Bjorvatn B, Mentzoni R, Hanss D, Huang K et al. Treatment of video game addiction-A systematic review. *Tidsskrift for Norsk Psykologforening.* 2015;52(6):471-478.
66. Park HS, Kwon YH, Park KM. [Factors on internet game addiction among adolescents]. *Kanho Hakhoe Chi.* 2007;37(5):754-761.
67. Pejovic-Milovancevic M, Popovic-Deusic S, Draganic-Gajic S, Lecic-Tosevski D. [Internet addiction--a case report]. *Srp Arh Celok Lek.* 2009;137(1-2):86-90.
68. Perez JIR. Pathological gambling and alcohol dependence in a sample of workers and university students: Prevalences, relationships and gender differences. *Psychologia: Avances de la Disciplina.* 2014;8(1):33-42.
69. Peukert P, Sieslack S, Barth G, Batra A. [Internet- and computer game addiction: phenomenology, comorbidity, etiology, diagnostics and therapeutic implications for the addictives and their relatives]. *Psychiatr Prax.* 2010;37(5):219-224.
70. Plessis C, Altintas E, Guerrien A. Online video game addiction: Comparative study of French evaluation scales. *Ann Med Psychol (Paris).* 2018;
71. Plusquellec M. Do virtual worlds threaten children's and teenagers mental health?. [French]. *Arch Pediatr.* 2000;7(2):209-210.
72. Potembska E, Pawlowska B. Severity of symptoms of internet addiction in secondary school girls using diets and laxatives. *Family Medicine and Primary Care Review.* 2010;12(3):797-799.
73. Prilutskaya MV, Kuliev RS. [Play behavior in people with various degrees of pathological gambling risk]. *Zh Nevrol Psikhiatr Im S S Korsakova.* 2015;115(3):25-29.
74. Rehbein F, Jukschat N, Mossle T. Computer game dependency in children and adolescents. [German]. *Padiatrische Praxis.* 2011; 52(3):671-682.
75. Ricquebourg M, Bernede-Bauduin C, Mete D, Dafreville C, Stojcic I, Vauthier M et al. [Internet and video games among students of Reunion Island in 2010: uses, misuses, perceptions and associated factors]. *Rev Epidemiol Sante Publique.* 2013;61(6):503-512.
76. Riedl D, Stockl A, Nussbaumer C, Rumpold G, Sevecke K, Fuchs M. [Usage patterns of internet and computer games : Results of an observational study of Tyrolean adolescents]. *Neuropsychiatrie.* 2016;30(4):181-190.
77. Rocher B, Grall-Bronnec M, Vandermerch N, Venisse JL. [Videogame addiction]. *Arch Pediatr.* 2008;15(5):804-805.
78. Rodriguez Monje MT, Pedrero Perez EJ, Fernandez Giron M, Gallardo Alonso F, Sanz Cuesta T. [Early detection of addictive behaviour in primary health care. Use of MULTICAGE CAD-4]. *Aten Primaria.* 2009;41(1):25-32.
79. Sanchez-Carbonell X, Beranuy M, Castellana M, Chamarro A, Oberst U. [Internet and cell phone addiction: passing fad or disorder?]. *Adicciones.* 2008;20(2):149-159.
80. Schuhler P, Vogelgesang M, Petry J. Pathological PC/Internet use: Clinical issues, diagnostic and therapeutic approaches. *Psychotherapeut.* 2009;54(3):187-192.

81. Suzuki FTI, Matias MV, Silva MTA, De Oliveira MPMT. The use of video, computer and internet games by a sample of college students from the University of Sao Paulo. [Portuguese]. *J Bras Psiquiatr.* 2009;58(3):162-168.
82. Taquet P, Hautekeete M, Gorgeu E. Cognitive, emotional, and behavioral determinants involved in the use of video games: Towards a better understanding of excessive gaming for CBT interventions. *Journal de Therapie Comportementale et Cognitive.* 2014;24(2):53-62.
83. Taquet P, Rolland B, Bence C, Lienard S, Cottencin O. [Identification of gaming and gambling behavior in addiction treatment centers by the regional network on gaming and gambling addiction in Northern France]. *Presse Med.* 2016;45(5):555-558.
84. Taquet P, Romo L, Cottencin O, Ortiz D, Hautekeete M. Video Game Addiction: Cognitive, emotional, and behavioral determinants for CBT treatment. *Journal de Therapie Comportementale et Cognitive.* 2017;27(3):118-128.
85. te Wildt BT, Putzig I, Zedler M, Ohlmeier MD. [Internet dependency as a symptom of depressive mood disorders]. *Psychiatr Prax.* 2007;34 Suppl 3:S318-322.
86. Tejeiro R. Video games addiction. A review. [Spanish]. *Adicciones.* 2001;13(4):407-413.
87. Thomasius R, Sack PM, Strittmatter E, Kaess M. [Substance-related and addictive disorders in the DSM-5]. *Z Kinder Jugendpsychiatr Psychother.* 2014;42(2):115-120.
88. Thorhauge AM, Brus AB. Computer games and addiction. *Paedagogisk Psykologisk Tidsskrift.* 2011;48(1):3-24.
89. Valleur M, Simonnet JF, Wellenstein A. [Better understanding of the new addictions]. *Rev Infirm.* 2010;(162):14-16.
90. Vlachopoulou X, Capart N. What is behind the screen? "Excessive players" online and their Rorschach results. *Psychologie Clinique et Projective.* 2013;19:223-252.
91. von Salisch M. [Computer use and internet time in children]. *Prax Kinderpsychol Kinderpsychiatr.* 2011;60(9):715-718.
92. Wartberg L, Aden A, Thomsen M, Thomasius R. [Relationships between family interactions and pathological internet use in adolescents: an review]. *Z Kinder Jugendpsychiatr Psychother.* 2015;43(1):9-17; quiz 18-19.
93. Wartberg L, Moll B, Baldus C, Thomsen M, Thomasius R. [Differences between adolescents with pathological Internet use in inpatient and outpatient treatment]. *Z Kinder Jugendpsychiatr Psychother.* 2017;45(4):313-322.
94. Wartberg L, Thomsen M, Moll B, Thomasius R. [Pilot study on the effectiveness of a cognitive behavioural group programme for adolescents with pathological internet use]. *Prax Kinderpsychol Kinderpsychiatr.* 2014;63(1):21-35.
95. Wolfling K, Muller KW, Beutel M. [Reliability and validity of the Scale for the Assessment of Pathological Computer-Gaming (CSV-S)]. *Psychother Psychosom Med Psychol.* 2011;61(5):216-224.
96. Xavier C. [The Internet Gaming Disorder in the DSM-5]. *Adicciones.* 2014;26(2):91-95.

Appendix C. Search strategy (MEDLINE, Embase, PsycINFO)

Ovid Multifile

Database: Ovid MEDLINE: Epub Ahead of Print, In-Process & Other Non-Indexed Citations, Ovid MEDLINE® Daily and Ovid MEDLINE® <1946-Present>, Embase Classic+Embase <1947 to 2018 May 14>, PsycINFO <1806 to May Week 1 2018>
Search Strategy:
----
1 IGD.tw,kf.
2 (gam* or internet* or disorder*).tw,kf.
3 1 and 2 [IGD]
4 Video Games/
5 gaming?.tw,kf.
6 videogam*.tw,kf.
7 ((game or games) adj3 (arcade? or console or consoles or computer* or digital* or internet* or mobile or offline or online or pc or video* or web or web-based or WWW)).tw,kf.
8 ((game or games) adj3 (iphon* or "i-phon*" or smartphon* or smart phon*)).tw,kf.
9 ((game or games) adj3 (android* or tablet*)).tw,kf.
10 ((game or games or play*) adj3 (Game boy or Game boys or Gameboy* or Gamecube* or "N Gage" or "NES Classic" or Nintendo* or playstation* or play station* or "Super NES" or Wii or Xbox*)).tw,kf.
11 ((game or games) adj3 (app or apps or Google Play or GooglePlay or iOS)).tw,kf.
12 ("call of duty*" or candy crush* or candycrush* or diablo* or "far cry*" or "first person shooter*" or "grand theft auto*" or "massively multi-player online*" or "massively multiplayer online*" or minecraft* or "mortal combat*" or "mortal kombat*" or "multi-player online battle arena*" or "multiplayer online battle arena*" or "pac man" or "pac manTM" or pokemon* or pong or sonic mania* or (sonic adj2 hedgehog*) or starcraft* or "super mario*" or tetris* or titanfall* or tomb raider* or wolfenstein* or "world of warcraft*" or zelda*).tw,kf.
13 or/4-12 [GAMING]
14 Behavior, Addictive/
15 Habits/
16 Health Behavior/
17 Health Risk Behaviors/
18 Social Behavior Disorders/
19 Video Games/ae [Adverse Effects]
20 Video Games/px [Psychology]
21 (addict* adj3 (computer* or digital* or internet or "i-phon*" or iphon* or online or smart phon* or smartphon* or video* or web or WWW)).tw,kf.
22 ((addict* or compulsive* or detriment* or disorder* or excess* or habitual* or hazardous* or obsess* or problem* or pathologic* or risky) adj5 behav*).tw,kf.
23 ((addict* or compulsive* or depend* or detriment* or disorder* or excess* or habitual* or hazardous* or impair* or obsess* or overus* or (over adj ("use" or used or uses or using)) or problem* or pathologic* or persist* or recur*) adj5 (game or games or gaming or videogam*)).tw,kf.
24 (habit or habits or habit-forming).tw,kf.
25 or/14-24 [PROBLEM BEHAVIOUR]
26 13 and 25 [PROBLEM GAMING BEHAVIOUR]
27 3 or 26 [IGD/PROBLEM GAMING]

28 exp Animals/ not (exp Animals/ and Humans/)
29 27 not 28 [ANIMAL-ONLY REMOVED]
30 29 use ppez [MEDLINE RECORDS]
31 IGD.tw,kw.
32 (gam* or internet* or disorder*).tw,kw.
33 31 and 32 [IGD]
34 video game/
35 gaming?.tw,kw.
36 videogam*.tw,kw.
37 ((game or games) adj3 (arcade? or console or consoles or computer* or digital* or internet* or mobile or offline or online or pc or video* or web or web-based or WWW)).tw,kw.
38 ((game or games) adj3 (iphon* or "i-phon*" or smartphon* or smart phon*)).tw,kw.
39 ((game or games) adj3 (android* or tablet*)).tw,kw.
40 ((game or games or play*) adj3 (Game boy or Game boys or Gameboy* or Gamecube* or "N Gage" or "NES Classic" or Nintendo* or playstation* or play station* or "Super NES" or Wii or Xbox*)).tw,kw.
41 ((game or games) adj3 (app or apps or Google Play or GooglePlay or iOS)).tw,kw.
42 ("call of duty*" or candy crush* or candycrush* or diablo* or "far cry*" or "first person shooter*" or "grand theft auto*" or "massively multi-player online*" or "massively multiplayer online*" or minecraft* or "mortal combat*" or "mortal kombat*" or "multi-player online battle arena*" or "multiplayer online battle arena*" or "pac man" or "pac manTM" or pokemon* or pong or sonic mania* or (sonic adj2 hedgehog*) or starcraft* or "super mario*" or tetris* or titanfall* or tomb raider* or wolfenstein* or "world of warcraft*" or zelda*).tw,kw.
43 or/34-42 [GAMING]
44 addiction/
45 behavior disorder/
46 behavioral addiction/
47 computer addiction/
48 game addiction/
49 habit/
50 health behavior/
51 high risk behavior/
52 problem behavior/
53 (addict* adj3 (computer* or digital* or internet or "i-phon*" or iphon* or online or smart phon* or smartphon* or video* or web or WWW)).tw,kw.
54 ((addict* or compulsive* or detriment* or disorder* or excess* or habitual* or hazardous* or obsess* or problem* or pathologic* or risky) adj5 behav*).tw,kw.
55 ((addict* or compulsive* or depend* or detriment* or disorder* or excess* or habitual* or hazardous* or impair* or obsess* or overus* or (over adj ("use" or used or uses or using)) or problem* or pathologic* or persist* or recur*) adj5 (game or games or gaming or videogam*)).tw,kw.
56 (habit or habits or habit-forming).tw,kw.
57 or/44-56 [PROBLEM BEHAVIOUR]
58 43 and 57 [PROBLEM GAMING BEHAVIOUR]
59 33 or 58 [IGD/PROBLEM GAMING]
60 exp animal/ or exp animal experimentation/ or exp animal model/ or exp animal experiment/ or nonhuman/ or exp vertebrate/
61 exp human/ or exp human experimentation/ or exp human experiment/
62 60 not 61
63 59 not 62 [ANIMAL-ONLY REMOVED]
64 63 use emczd [EMBASE RECORDS]

65 IGD.tw.
66 (gam* or internet* or disorder*).tw.
67 65 and 66 [IGD]
68 Computer Games/
69 gaming?.tw.
70 videogam*.tw.
71 ((game or games) adj3 (arcade? or console or consoles or computer* or digital* or internet* or mobile or offline or online or pc or video* or web or web-based or WWW)).tw.
72 ((game or games) adj3 (iphon* or "i-phon*" or smartphon* or smart phon*)).tw.
73 ((game or games) adj3 (android* or tablet*)).tw.
74 ((game or games or play*) adj3 (Game boy or Game boys or Gameboy* or Gamecube* or "N Gage" or "NES Classic" or Nintendo* or playstation* or play station* or "Super NES" or Wii or Xbox*)).tw.
75 ((game or games) adj3 (app or apps or Google Play or GooglePlay or iOS)).tw.
76 ("call of duty*" or candy crush* or candycrush* or diablo* or "far cry*" or "first person shooter*" or "grand theft auto*" or "massively multi-player online*" or "massively multiplayer online*" or minecraft* or "mortal combat*" or "mortal kombat*" or "multi-player online battle arena*" or "multiplayer online battle arena*" or "pac man" or "pac manTM" or pokemon* or pong or sonic mania* or (sonic adj2 hedgehog*) or starcraft* or "super mario*" or tetris* or titanfall* or tomb raider* or wolfenstein* or "world of warcraft*" or zelda*).tw.
77 or/68-76 [GAMING]
78 Addiction/
79 Behavior Disorders/
80 Behavior Problems/
81 Habits/
82 Health Behavior/
83 Internet Addiction/
84 (addict* adj3 (computer* or digital* or internet or "i-phon*" or iphon* or online or smart phon* or smartphon* or video* or web or WWW)).tw.
85 ((addict* or compulsive* or detriment* or disorder* or excess* or habitual* or hazardous* or obsess* or problem* or pathologic* or risky) adj5 behav*).tw.
86 ((addict* or compulsive* or depend* or detriment* or disorder* or excess* or habitual* or hazardous* or impair* or obsess* or overus* or (over adj ("use" or used or uses or using)) or problem* or pathologic* or persist* or recur*) adj5 (game or games or gaming or videogam*)).tw.
87 (habit or habits or habit-forming).tw.
88 or/78-87 [PROBLEM BEHAVIOUR]
89 77 and 88 [PROBLEM GAMING BEHAVIOUR]
90 67 or 89 [IGD/PROBLEM GAMING]
91 exp Animals/ not (exp Animals/ and Humans/)
92 90 not 91 [ANIMAL-ONLY REMOVED]
93 92 use ppez,emczd
94 92 not 93 [PSYCINFO RECORDS]
95 30 or 64 or 94 [ALL DATABASES]
96 limit 95 to yr="2014-current"
97 remove duplicates from 96
98 95 not 96
99 remove duplicates from 98
100 97 or 99 [TOTAL UNIQUE RECORDS]
101 100 use ppez [MEDLINE UNIQUE RECORDS]
102 100 use emczd [EMBASE UNIQUE RECORDS]
103 100 not (101 or 102) [PSYCINFO UNIQUE RECORDS]

Appendix D. List of included full-text articles

1. Adams BL, Stavropoulos V, Burleigh TL, Liew LW, Beard CL, Griffiths MD. Internet gaming disorder behaviors in emergent adulthood: A pilot study examining the interplay between anxiety and family cohesion. *Int J Ment Health Addict.* 2018:1-17.
2. Allen JJ, Anderson CA. Satisfaction and frustration of basic psychological needs in the real world and in video games predict internet gaming disorder scores and well-being. *Comput Human Behav.* 2018;84:220-229.
3. Arcelus J, Bouman WP, Jones BA, Richards C, Jimenez-Murcia S, Griffiths MD. Video gaming and gaming addiction in transgender people: An exploratory study. *J Behav Addict.* 2017;6(1):21-29.
4. Bae S, Han DH, Jung J, Nam KC, Renshaw PF. Comparison of brain connectivity between Internet gambling disorder and Internet gaming disorder: A preliminary study. *J Behav Addict.* 2017;6(4):505-515.
5. Bae S, Hong JS, Kim SM, Han DH. Bupropion Shows Different Effects on Brain Functional Connectivity in Patients With Internet-Based Gambling Disorder and Internet Gaming Disorder. *Front Psychiatry.* 2018;9:130.
6. Bargeron AH, Hormes JM. Psychosocial correlates of internet gaming disorder: Psychopathology, life satisfaction, and impulsivity. *Comput Human Behav.* 2017;68:388-394.
7. Bouna-Pyrrou P, Muhle C, Kornhuber J, Lenz B. Internet gaming disorder, social network disorder and laterality: handedness relates to pathological use of social networks. *J Neural Transm.* 2015;122(8):1187-1196.
8. Buiza-Aguado C, Alonso-Canovas A, Conde-Mateos C, Buiza-Navarrete JJ, Gentile D. Problematic Video Gaming in a Young Spanish Population: Association with Psychosocial Health. *Cyberpsychol Behav Soc Netw.* 2018;21(6):388-394.
9. Burleigh TL, Stavropoulos V, Liew LW, Adams BL, Griffiths MD. Depression, internet gaming disorder, and the moderating effect of the gamer-avatar relationship: An exploratory longitudinal study. *Int J Ment Health Addict.* 2018;16(1):102-124.
10. Cai C, Yuan K, Yin J, Feng D, Bi Y, Li Y et al. Striatum morphometry is associated with cognitive control deficits and symptom severity in internet gaming disorder. *Brain Imaging Behav.* 2016;10(1):12-20.
11. Carlisle KL. Personality, motivation, and internet gaming disorder: Understanding the addiction. *Diss Abstr Int: Section B: The Sciences and Engineering.* 2018;78(11-B(E))
12. Cho YU, Lee D, Lee JE, Kim KH, Lee DY, Jung YC. Exploratory metabolomics of biomarker identification for the internet gaming disorder in young Korean males. *J Chromatogr B Analyt Technol Biomed Life Sci.* 2017;1057:24-31.
13. Choi J, Cho H, Kim JY, Jung DJ, Ahn KJ, Kang HB et al. Structural alterations in the prefrontal cortex mediate the relationship between Internet gaming disorder and depressed mood. *Sci Rep.* 2017;7(1):1245.
14. Choi SW, Kim HS, Kim GY, Jeon Y, Park SM, Lee JY et al. Similarities and differences among Internet gaming disorder, gambling disorder and alcohol use disorder: a focus on impulsivity and compulsivity. *J Behav Addict.* 2014;3(4):246-253.
15. De Pasquale C, Dinaro C, Sciacca F. Relationship of Internet gaming disorder with dissociative experience in Italian university students. *Ann Gen Psychiatry.* 2018;17:28.
16. Deleuze J, Nuyens F, Rochat L, Rothen S, Maurage P, Billieux J. Established risk factors for addiction fail to discriminate between healthy gamers and gamers endorsing DSM-5 Internet gaming disorder. *J Behav Addict.* 2017;6(4):516-524.

17. Dong G, Li H, Wang L, Potenza MN. Cognitive control and reward/loss processing in Internet gaming disorder: Results from a comparison with recreational Internet game-users. *Eur Psychiatry.* 2017a;44:30-38.
18. Dong G, Li H, Wang L, Potenza MN. The correlation between mood states and functional connectivity within the default mode network can differentiate Internet gaming disorder from healthy controls. *Prog Neuropsychopharmacol Biol Psychiatry.* 2017b;77:185-193.
19. Dong G, Wang L, Du X, Potenza MN. Gaming Increases Craving to Gaming-Related Stimuli in Individuals With Internet Gaming Disorder. *Biol Psychiatry Cogn Neurosci Neuroimaging.* 2017c;2(5):404-412.
20. Dong G WZ, Wang Y, Du X, Potenza MN. Gender-related functional connectivity and craving during gaming and immediate abstinence during a mandatory break: Implications for development and progression of internet gaming disorder. *Prog Neuropsychopharmacol Biol Psychiatry.* 2018b;88:1-10.
21. Dong G WL, Wang Z, Wang Y, Du X, Potenza MN. Diffusion-weighted MRI measures suggest increased white-matter integrity in Internet gaming disorder: Evidence from the comparison with recreational Internet game users. *Addict Behav.* 2018a;81:32-38.
22. Evren C, Dalbudak E, Topcu M, Kutlu N, Evren B. The psychometric properties of the Turkish version of the Internet Gaming Disorder Scale. *Dusunen Adam.* 2017;30(4):316-324.
23. Evren C, Dalbudak E, Topcu M, Kutlu N, Evren B, Pontes HM. Psychometric validation of the Turkish nine-item Internet Gaming Disorder Scale-Short Form (IGDS9-SF). *Psychiatry Res.* 2018;265:349-354.
24. Fu W, Yu CK-C. Predicting disordered gambling with illusory control, gaming preferences, and Internet gaming addiction among Chinese youth. *Int J Ment Health Addict.* 2015;13(3):391-401.
25. Fuster H, Carbonell X, Pontes HM, Griffiths MD. Spanish validation of the Internet Gaming Disorder-20 (IGD-20) Test. *Comput Human Behav.* 2016;56:215-224.
26. Gonzalez-Bueso V, Santamaria JJ, Fernandez D, Merino L, Montero E, Jimenez-Murcia S et al. Internet Gaming Disorder in Adolescents: Personality, Psychopathology and Evaluation of a Psychological Intervention Combined With Parent Psychoeducation. *Front Psychol.* 2018;9:787.
27. Gunuc S. Relationships and associations between video game and Internet addictions: Is tolerance a symptom seen in all conditions. *Comput Human Behav.* 2015;49:517-525.
28. Han JW, Han DH, Bolo N, Kim B, Kim BN, Renshaw PF. Differences in functional connectivity between alcohol dependence and internet gaming disorder. *Addict Behav.* 2015;41:12-19.
29. Hawi NS, Samaha M, Griffiths MD. Internet gaming disorder in Lebanon: Relationships with age, sleep habits, and academic achievement. *J Behav Addict.* 2018;7(1):70-78.
30. Jeong H, Yim HW, Lee SY, Lee HK, Potenza MN, Kwon JH et al. Discordance between self-report and clinical diagnosis of Internet gaming disorder in adolescents. *Sci Rep.* 2018;8(1):10084.
31. Jeong JE, Rhee JK, Kim TM, Kwak SM, Bang SH, Cho H et al. The association between the nicotinic acetylcholine receptor alpha4 subunit gene (CHRNA4) rs1044396 and Internet gaming disorder in Korean male adults. *PLoS One.* 2017;12(12):e0188358.
32. Jeromin F, Rief W, Barke A. Using two web-based addiction Stroops to measure the attentional bias in adults with Internet Gaming Disorder. *J Behav Addict.* 2016a;5(4):666-673.
33. Jeromin F, Rief W, Barke A. Validation of the Internet Gaming Disorder Questionnaire in a Sample of Adult German-Speaking Internet Gamers. *Cyberpsychol Behav Soc Netw.* 2016b;19(7):453-459.

34. Jo SJ, Yim HW, Lee HK, Lee HC, Choi JS, Baek KY. The Internet Game Use-Elicited Symptom Screen proved to be a valid tool for adolescents aged 10-19 years. *Acta Paediatr.* 2018;107(3):511-516.
35. Kaess M, Parzer P, Mehl L, Weil L, Strittmatter E, Resch F et al. Stress vulnerability in male youth with Internet Gaming Disorder. *Psychoneuroendocrinology.* 2017;77:244-251.
36. Kaptsis D, King DL, Delfabbro PH, Gradisar M. Trajectories of abstinence-induced Internet gaming withdrawal symptoms: A prospective pilot study. *Addict Behav Rep.* 2016;4:24-30.
37. Kim H, Kim YK, Gwak AR, Lim JA, Lee JY, Jung HY et al. Resting-state regional homogeneity as a biological marker for patients with Internet gaming disorder: A comparison with patients with alcohol use disorder and healthy controls. *Prog Neuropsychopharmacol Biol Psychiatry.* 2015;60:104-111.
38. Kim JY, Jeong JE, Rhee JK, Cho H, Chun JW, Kim TM et al. Targeted exome sequencing for the identification of a protective variant against Internet gaming disorder at rs2229910 of neurotrophic tyrosine kinase receptor, type 3 (NTRK3): A pilot study. *J Behav Addict.* 2016a;5(4):631-638.
39. Kim M, Lee TH, Choi JS, Kwak YB, Hwang WJ, Kim T et al. Neurophysiological correlates of altered response inhibition in internet gaming disorder and obsessive-compulsive disorder: Perspectives from impulsivity and compulsivity. *Sci Rep.* 2017a;7:41742.
40. Kim NR, Hwang SS, Choi JS, Kim DJ, Demetrovics Z, Kiraly O et al. Characteristics and Psychiatric Symptoms of Internet Gaming Disorder among Adults Using Self-Reported DSM-5 Criteria. *Psychiatry Investig.* 2016b;13(1):58-66.
41. Kim YJ, Lee JY, Oh S, Park M, Jung HY, Sohn BK et al. Associations between prospective symptom changes and slow-wave activity in patients with Internet gaming disorder: A resting-state EEG study. *Medicine.* 2017b;96(8):e6178.
42. Kim YJ, Lim JA, Lee JY, Oh S, Kim SN, Kim DJ et al. Impulsivity and compulsivity in Internet gaming disorder: A comparison with obsessive-compulsive disorder and alcohol use disorder. *J Behav Addict.* 2017c;6(4):545-553.
43. King DL, Adair C, Saunders JB, Delfabbro PH. Clinical predictors of gaming abstinence in help-seeking adult problematic gamers. *Psychiatry Res.* 2018a;261:581-588.
44. King DL, Delfabbro PH. The Cognitive Psychopathology of Internet Gaming Disorder in Adolescence. *J Abnorm Child Psychol.* 2016;44(8):1635-1645.
45. King DL, Delfabbro PH. Features of parent-child relationships in adolescents with Internet gaming disorder. *Int J Ment Health Addict.* 2017c;15(6):1270-1283.
46. King DL, Herd MC, Delfabbro PH. Motivational components of tolerance in internet gaming disorder. *Comput Human Behav.* 2018b;78:133-141.
47. King DL, Kaptsis D, Delfabbro PH, Gradisar M. Effectiveness of Brief Abstinence for Modifying Problematic Internet Gaming Cognitions and Behaviors. *J Clin Psychol.* 2017b;73(12):1573-1585.
48. Kiraly O, Sleczka P, Pontes HM, Urban R, Griffiths MD, Demetrovics Z. Validation of the Ten-Item Internet Gaming Disorder Test (IGDT-10) and evaluation of the nine DSM-5 Internet Gaming Disorder criteria. *Addict Behav.* 2017;64:253-260.
49. Ko CH, Wang PW, Liu TL, Chen CS, Yen CF, Yen JY. The adaptive decision-making, risky decision, and decision-making style of Internet gaming disorder. *Eur Psychiatry.* 2017;44:189-197.
50. Ko CH, Yen JY, Chen SH, Wang PW, Chen CS, Yen CF. Evaluation of the diagnostic criteria of Internet gaming disorder in the DSM-5 among young adults in Taiwan.[Erratum appears in J Psychiatr Res. 2014 Oct;57():185]. *J Psychiatr Res.* 2014;53:103-110.

51. Laconi S, Pires S, Chabrol H. Internet gaming disorder, motives, game genres and psychopathology. *Comput Human Behav.* 2017;75:652-659.
52. Lee D, Hong SJ, Jung YC, Park J, Kim IY, Namkoong K. Altered Heart Rate Variability During Gaming in Internet Gaming Disorder. *Cyberpsychol Behav Soc Netw.* 2018a;21(4):259-267.
53. Lee D, Lee J, Yoon KJ, Kee N, Jung YC. Impaired anterior insular activation during risky decision making in young adults with internet gaming disorder. *Neuroreport.* 2016;27(8):605-609.
54. Lee D, Namkoong K, Lee J, Jung YC. Abnormal gray matter volume and impulsivity in young adults with Internet gaming disorder. *Addict Biol.* 2017a;08:08.
55. Lee D, Namkoong K, Lee J, Jung YC. Preliminary evidence of altered gray matter volume in subjects with internet gaming disorder: associations with history of childhood attention-deficit/hyperactivity disorder symptoms. *Brain Imaging Behav.* 2018b;11:11.
56. Lee J, Lee S, Chun JW, Cho H, Kim DJ, Jung YC. Compromised Prefrontal Cognitive Control Over Emotional Interference in Adolescents with Internet Gaming Disorder. *Cyberpsychol Behav Soc Netw.* 2015;18(11):661-668.
57. Lee M, Cho H, Jung SH, Yim SH, Cho SM, Chun JW et al. Circulating MicroRNA Expression Levels Associated With Internet Gaming Disorder. *Front Psychiatry.* 2018c;9:81.
58. Lee SY, Lee HK, Jeong H, Yim HW, Bhang SY, Jo SJ et al. The Hierarchical Implications of Internet Gaming Disorder Criteria: Which Indicate more Severe Pathology? *Psychiatry Investig.* 2017b;14(3):249-259.
59. Lemmens JS, Hendriks SJ. Addictive Online Games: Examining the Relationship Between Game Genres and Internet Gaming Disorder. *Cyberpsychol Behav Soc Netw.* 2016;19(4):270-276.
60. Lemmens JS, Valkenburg PM, Gentile DA. The Internet Gaming Disorder Scale. *Psychol Assess.* 2015;27(2):567-582.
61. Li AY, Lo BC, Cheng C. It Is the Family Context That Matters: Concurrent and Predictive Effects of Aspects of Parent-Child Interaction on Video Gaming-Related Problems. *Cyberpsychol Behav Soc Netw.* 2018a;21(6):374-380.
62. Li W. Mindfulness-Oriented Recovery Enhancement for video game addiction in U.S. emerging adults. *Diss Abstr Int: Section A: Humanities and Social Sciences.* 2017a; 77(11-A(E)).
63. Li W, Garland EL, Howard MO. Therapeutic mechanisms of Mindfulness-Oriented Recovery Enhancement for internet gaming disorder: Reducing craving and addictive behavior by targeting cognitive processes. *J Addict Dis.* 2018b:1-9.
64. Li W, Garland EL, McGovern P, O'Brien JE, Tronnier C, Howard MO. Mindfulness-oriented recovery enhancement for internet gaming disorder in U.S. adults: A stage I randomized controlled trial. *Psychol Addict Behav.* 2017b;31(4):393-402.
65. Liew LW, Stavropoulos V, Adams BL, Burleigh TL, Griffiths MD. Internet gaming disorder: The interplay between physical activity and user-avatar relationship. *Behav Inf Technol.* 2018;37(6):558-574.
66. Lim JA, Lee JY, Jung HY, Sohn BK, Choi SW, Kim YJ et al. Changes of quality of life and cognitive function in individuals with Internet gaming disorder: A 6-month follow-up. *Medicine.* 2016;95(50):e5695.
67. Lopez-Fernandez O, Honrubia-Serrano M, Baguley T, Griffiths MD. Pathological video game playing in Spanish and British adolescents: Towards the exploration of Internet Gaming Disorder symptomatology. *Comput Human Behav.* 2014;41:304-312.
68. Mallorqui-Bague N, Fernandez-Aranda F, Lozano-Madrid M, Granero R, Mestre-Bach G, Bano M et al. Internet gaming disorder and online gambling disorder: Clinical and personality correlates. *J Behav Addict.* 2017;6(4):669-677.

69. Martin-Fernandez M, Matali JL, Garcia-Sanchez S, Pardo M, Lleras M, Castellano-Tejedor C. Adolescents with Internet Gaming Disorder (IGD): profiles and treatment response. *Adicciones.* 2017;29(2):125-133.
70. Milani L, La Torre G, Fiore M, Grumi S, Gentile DA, Ferrante M et al. Internet gaming addiction in adolescence: Risk factors and maladjustment correlates. *Int J Ment Health Addict.* 2018;16(4):888-904.
71. Monacis L, de Palo V, Griffiths MD, Sinatra M. Exploring Individual Differences in Online Addictions: the Role of Identity and Attachment. *Int J Ment Health Addict.* 2017;15(4):853-868.
72. Monacis L, de Palo V, Griffiths MD, Sinatra M. Validation of the Internet Gaming Disorder Scale - Short-Form (IGDS9-SF) in an Italian-speaking sample. *J Behav Addict.* 2016;5(4):683-690.
73. Monacis L, Sinatra M, Griffiths MD, de Palo V. Assessment of the Italian Version of the Internet Disorder Scale (IDS-15). *Int J Ment Health Addict.* 2018;16(3):680-691.
74. Na E, Choi I, Lee TH, Lee H, Rho MJ, Cho H et al. The influence of game genre on Internet gaming disorder. *J Behav Addict.* 2017a:1-8.
75. Na E, Lee H, Choi I, Kim DJ. Comorbidity of Internet gaming disorder and alcohol use disorder: A focus on clinical characteristics and gaming patterns. *Am J Addict.* 2017b;26(4):326-334.
76. Paik SH, Cho H, Chun JW, Jeong JE, Kim DJ. Gaming Device Usage Patterns Predict Internet Gaming Disorder: Comparison across Different Gaming Device Usage Patterns. *Int J Environ Res Public Health.* 2017a;14(12):05.
77. Paik SH, Choi MR, Kwak SM, Bang SH, Chun JW, Kim JY et al. An association study of Taq1A ANKK1 and C957T and - 141C DRD2 polymorphisms in adults with internet gaming disorder: a pilot study. *Ann Gen Psychiatry.* 2017b;16:45.
78. Palo V, Monacis L, Sinatra M, Griffiths MD, Pontes H, Petro M et al. Measurement invariance of the nine-item internet gaming disorder scale (IGDS9-SF) across Albania, USA, UK, and Italy. *Int J Ment Health Addict.* 2018:1-12.
79. Park JH, Hong JS, Han DH, Min KJ, Lee YS, Kee BS et al. Comparison of QEEG Findings between Adolescents with Attention Deficit Hyperactivity Disorder (ADHD) without Comorbidity and ADHD Comorbid with Internet Gaming Disorder. *J Korean Med Sci.* 2017a;32(3):514-521.
80. Park JHL, YS.; Sohn, JH.; Han, DH. Effectiveness of atomoxetine and methylphenidate for problematic online gaming in adolescents with attention deficit hyperactivity disorder. *Hum Psychopharmacol.* 2016a;31(6):427-432.
81. Park M, Choi JS, Park SM, Lee JY, Jung HY, Sohn BK et al. Dysfunctional information processing during an auditory event-related potential task in individuals with Internet gaming disorder. *Transl Psychiatry.* 2016b;6:e721.
82. Park M, Kim YJ, Kim DJ, Choi JS. Differential neurophysiological correlates of information processing in Internet gaming disorder and alcohol use disorder measured by event-related potentials. *Sci Rep.* 2017b;7(1):9062.
83. Park S, Ryu H, Lee JY, Choi A, Kim DJ, Kim SN et al. Longitudinal Changes in Neural Connectivity in Patients With Internet Gaming Disorder: A Resting-State EEG Coherence Study. *Front Psychiatry.* 2018;9:252.
84. Park SM, Lee JY, Kim YJ, Lee JY, Jung HY, Sohn BK et al. Neural connectivity in Internet gaming disorder and alcohol use disorder: A resting-state EEG coherence study. *Sci Rep.* 2017d;7(1):1333.
85. Pearcy BT, McEvoy PM, Roberts LD. Internet Gaming Disorder Explains Unique Variance in Psychological Distress and Disability After Controlling for Comorbid Depression, OCD, ADHD, and Anxiety. *Cyberpsychol Behav Soc Netw.* 2017;20(2):126-132.

86. Pearcy BT, Roberts LD, McEvoy PM. Psychometric Testing of the Personal Internet Gaming Disorder Evaluation-9: A New Measure Designed to Assess Internet Gaming Disorder. *Cyberpsychol Behav Soc Netw.* 2016;19(5):335-341.
87. Peeters M, Koning I, van den Eijnden R. Predicting Internet Gaming Disorder symptoms in young adolescents: A one-year follow-up study. *Comput Human Behav.* 2018;80:255-261.
88. Pontes HM. Investigating the differential effects of social networking site addiction and Internet gaming disorder on psychological health. *J Behav Addict.* 2017a;6(4):601-610.
89. Park M, Kim YJ, Kim DJ, Choi JS. Sustained dysfunctional information processing in patients with Internet gaming disorder: 6-month follow-up ERP study. *Medicine.* 2017c;96(36):e7995.
90. King DL, Herd MCE, Delfabbro PH. Tolerance in Internet gaming disorder: A need for increasing gaming time or something else? *J Behav Addict.* 2017a;6(4):525-533.
91. Koo HJ, Han DH, Park SY, Kwon JH. The Structured Clinical Interview for DSM-5 Internet Gaming Disorder: Development and Validation for Diagnosing IGD in Adolescents. *Psychiatry Investig.* 2017;14(1):21-29.
92. Krossbakken E, Torsheim T, Mentzoni RA, King DL, Bjorvatn B, Lorvik IM et al. The effectiveness of a parental guide for prevention of problematic video gaming in children: A public health randomized controlled intervention study. *J Behav Addict.* 2018;7(1):52-61.
93. Pontes HM, Griffiths MD. Measuring DSM-5 Internet Gaming Disorder: Development and validation of a short psychometric scale. *Comput Human Behav.* 2015;45:137-143.
94. Pontes HM, Kiraly O, Demetrovics Z, Griffiths MD. The conceptualisation and measurement of DSM-5 Internet Gaming Disorder: the development of the IGD-20 Test. *PLoS One.* 2014;9(10):e110137.
95. Pontes HM, Macur M, Griffiths MD. Internet Gaming Disorder Among Slovenian Primary Schoolchildren: Findings From a Nationally Representative Sample of Adolescents. *J Behav Addict.* 2016;5(2):304-310.
96. Pontes HM, Stavropoulos V, Griffiths MD. Measurement Invariance of the Internet Gaming Disorder Scale-Short-Form (IGDS9-SF) between the United States of America, India and the United Kingdom. *Psychiatry Res.* 2017b;257:472-478.
97. Przybylski AK. Mischievous responding in Internet Gaming Disorder research. *PeerJ.* 2016a;4:e2401.
98. Przybylski AK. Mischievous responding in Internet Gaming Disorder research. *PeerJ*. 2016b;4:e2401.
99. Przybylski AK, Weinstein N, Murayama K. Internet Gaming Disorder: Investigating the Clinical Relevance of a New Phenomenon. *Am J Psychiatry.* 2017a;174(3):230-236.
100. Przybylski AK, Weinstein N, Murayama K. Internet Gaming Disorder: Investigating the Clinical Relevance of a New Phenomenon. *Am J Psychiatry*. 2017b;174(3):230-236.
101. Przybylski AK, Weinstein N, Murayama K. Internet Gaming Disorder: Investigating the Clinical Relevance of a New Phenomenon. *Am J Psychiatry*. 2017c;174(3):230-236.
102. Przybylski AK, Weinstein N, Murayama K. Internet Gaming Disorder: Investigating the Clinical Relevance of a New Phenomenon. *Am J Psychiatry*. 2017d;174(3):230-236.
103. Rao J, Wang H, Pang M, Yang J, Zhang J, Ye Y et al. Cyberbullying perpetration and victimisation among junior and senior high school students in Guangzhou, China. *Inj Prev.* 2017;06:06.
104. Rehbein F, Kliem S, Baier D, Mosle T, Petry NM. Prevalence of Internet gaming disorder in German adolescents: diagnostic contribution of the nine DSM-5 criteria in a state-wide representative sample. *Addiction.* 2015;110(5):842-851.

105. Rho MJ, Jeong JE, Chun JW, Cho H, Jung DJ, Choi IY et al. Predictors and patterns of problematic Internet game use using a decision tree model. *J Behav Addict.* 2016;5(3):500-509.
106. Rho MJ, Lee H, Lee TH, Cho H, Jung DJ, Kim DJ et al. Risk Factors for Internet Gaming Disorder: Psychological Factors and Internet Gaming Characteristics. *Int J Environ Res Public Health.* 2017;15(1):27.
107. Thomsen KR, Callesen MB, Hesse M, Kvamme TL, Pedersen MM, Pedersen MU et al. Impulsivity traits and addiction-related behaviors in youth. *J Behav Addict.* 2018;7(2):317-330.
108. Ryu H, Lee JY, Choi A, Park S, Kim DJ, Choi JS. The Relationship between Impulsivity and Internet Gaming Disorder in Young Adults: Mediating Effects of Interpersonal Relationships and Depression. *Int J Environ Res Public Health.* 2018;15(3):06.
109. Sakuma H, Mihara S, Nakayama H, Miura K, Kitayuguchi T, Maezono M et al. Treatment with the Self-Discovery Camp (SDiC) improves Internet gaming disorder. *Addict Behav.* 2017;64:357-362.
110. Sanders J, Williams R, Damgaard M. Video Game Play and Internet Gaming Disorder Among Canadian Adults: A National Survey. *Can J Addict.* 2017;8(2):6-12.
111. Schneider LA, King DL, Delfabbro PH. Maladaptive coping styles in adolescents with internet gaming disorder symptoms. *Int J Ment Health Addict.* 2018;16(4):905-916.
112. Seok JW, Sohn JH. Altered Gray Matter Volume and Resting-State Connectivity in Individuals With Internet Gaming Disorder: A Voxel-Based Morphometry and Resting-State Functional Magnetic Resonance Imaging Study. *Front Psychiatry.* 2018;9:77.
113. Shin YB, Kim JJ, Kim MK, Kyeong S, Jung YH, Eom H et al. Development of an effective virtual environment in eliciting craving in adolescents and young adults with internet gaming disorder. *PLoS One.* 2018;13(4):e0195677.
114. Sigerson L, Li AY, Cheung MW, Luk JW, Cheng C. Psychometric properties of the Chinese Internet Gaming Disorder Scale. *Addict Behav.* 2017;74:20-26.
115. Sioni SR, Burleson MH, Bekerian DA. Internet gaming disorder: Social phobia and identifying with your virtual self. *Comput Human Behav.* 2017;71:11-15.
116. Snodgrass JG, Dengah Ii HJF, Lacy MG, Else RJ, Polzer ER, Arevalo JMG et al. Social genomics of healthy and disordered internet gaming. *Am J Hum Biol.* 2018;30(5):e23146.
117. Son KL, Choi JS, Lee J, Park SM, Lim JA, Lee JY et al. Neurophysiological features of Internet gaming disorder and alcohol use disorder: a resting-state EEG study. *Transl Psychiatry Psychiatry.* 2015;5:e628.
118. Song J, Park JH, Han DH, Roh S, Son JH, Choi TY et al. Comparative study of the effects of bupropion and escitalopram on Internet gaming disorder. *Psychiatry Clin Neurosci.* 2016;70(11):527-535.
119. Stavropoulos V, Beard C, Griffiths MD, Buleigh T, Gomez R, Pontes HM. Measurement Invariance of the Internet Gaming Disorder Scale-Short-Form (IGDS9-SF) Between Australia, the USA, and the UK. *Int J Ment Health Addict.* 2018;16(2):377-392.
120. Stockdale L, Coyne SM. Video game addiction in emerging adulthood: Cross-sectional evidence of pathology in video game addicts as compared to matched healthy controls. *J Affect Disord.* 2018;225:265-272.
121. Stubblefield S, Datto G, Phan TT, Werk LN, Stackpole K, Siegel R et al. Problem Video Gaming Among Children Enrolled in Tertiary Weight Management Programs. *Cyberpsychol Behav Soc Netw.* 2017;20(2):109-116.
122. Subramaniam M, Chua BY, Abdin E, Pang S, Satghare P, Vaingankar JA et al. Prevalence and Correlates of Internet Gaming Problem among Internet Users: Results from an Internet Survey. *Ann Acad Med Singapore.* 2016;45(5):174-183.

123. Taquet P, Romo L, Cottencin O, Ortiz D, Hautekeete M. Video Game Addiction: Cognitive, emotional, and behavioral determinants for CBT treatment. *Journal de Therapie Comportementale et Cognitive.* 2017;27(3):118-128.
124. Tejeiro R, Espada J, Gonzalvez M, Christiansen P. Psychometric properties of the Problem Video Game Playing scale in adults. *Eur Rev Appl Psychol.* 2016;66(1):9-13.
125. Tian M, Tao R, Zheng Y, Zhang H, Yang G, Li Q et al. Internet gaming disorder in adolescents is linked to delay discounting but not probability discounting. *Comput Human Behav.* 2018;80:59-66.
126. Triberti S, Milani L, Villani D, Grumi S, Peracchia S, Curcio G et al. What matters is when you play: Investigating the relationship between online video games addiction and time spent playing over specific day phases. *Addict Behav Rep.* 2018;
127. Vadlin S, Aslund C, Nilsson KW. A longitudinal study of the individual- and group-level problematic gaming and associations with problem gambling among Swedish adolescents. *Brain and Behavior.* 2018;8(4):e00949.
128. van Rooij AJ, Schoenmakers TM, van de Mheen D. Clinical validation of the C-VAT 2.0 assessment tool for gaming disorder: A sensitivity analysis of the proposed DSM-5 criteria and the clinical characteristics of young patients with 'video game addiction'. *Addict Behav.* 2017;64:269-274.
129. Wang CY, Wu YC, Su CH, Lin PC, Ko CH, Yen JY. Association between Internet gaming disorder and generalized anxiety disorder. *J Behav Addict.* 2017a;6(4):564-571.
130. Wang HR, Cho H, Kim DJ. Prevalence and correlates of comorbid depression in a nonclinical online sample with DSM-5 internet gaming disorder. *J Affect Disord.* 2018a;226:1-5.
131. Wang L, Wu L, Lin X, Zhang Y, Zhou H, Du X et al. Altered brain functional networks in people with Internet gaming disorder: Evidence from resting-state fMRI. *Psychiatry Res Neuroimaging.* 2016a;254:156-163.
132. Wang L, Wu L, Lin X, Zhang Y, Zhou H, Du X et al. Dysfunctional default mode network and executive control network in people with Internet gaming disorder: Independent component analysis under a probability discounting task. *Eur Psychiatry.* 2016b;34:36-42.
133. Wang L, Wu L, Wang Y, Li H, Liu X, Du X et al. Altered Brain Activities Associated with Craving and Cue Reactivity in People with Internet Gaming Disorder: Evidence from the Comparison with Recreational Internet Game Users. *Front Psychol.* 2017b;8:1150.
134. Wang L, Zhang Y, Lin X, Zhou H, Du X, Dong G. Group independent component analysis reveals alternation of right executive control network in Internet gaming disorder. *Cns Spectrums.* 2017c:1-11.
135. Wang S, Liu J, Tian L, Chen L, Wang J, Tang Q et al. Increased Insular Cortical Thickness Associated With Symptom Severity in Male Youths With Internet Gaming Disorder: A Surface-Based Morphometric Study. *Front Psychiatry.* 2018b;9:99.
136. Wang Y, Hu Y, Xu J, Zhou H, Lin X, Du X et al. Dysfunctional Prefrontal Function Is Associated with Impulsivity in People with Internet Gaming Disorder during a Delay Discounting Task. *Front Psychiatry.* 2017d;8:287.
137. Wang Y, Wu L, Wang L, Zhang Y, Du X, Dong G. Impaired decision-making and impulse control in Internet gaming addicts: evidence from the comparison with recreational Internet game users. Addict Biol. 2017f;22(6):1610-1621.
138. Wang Y, Wu L, Zhou H, Lin X, Zhang Y, Du X et al. Impaired executive control and reward circuit in Internet gaming addicts under a delay discounting task: independent component analysis. *Eur Arch Psychiatry Clin Neurosci.* 2017e;267(3):245-255.
139. Wang Z, Wu L, Yuan K, Hu Y, Zheng H, Du X et al. Cortical thickness and volume abnormalities in Internet gaming disorder: Evidence from comparison of recreational Internet game users. *Eur J Neurosci.* 2018c;08:08.

140. Wartberg L, Kriston L, Kammerl R. Associations of Social Support, Friends Only Known Through the Internet, and Health-Related Quality of Life with Internet Gaming Disorder in Adolescence. *Cyberpsychol Behav Soc Netw.* 2017a;20(7):436-441.
141. Wartberg L, Kriston L, Kramer M, Schwedler A, Lincoln TM, Kammerl R. Internet gaming disorder in early adolescence: Associations with parental and adolescent mental health. *Eur Psychiatry.* 2017b;43:14-18.
142. Wartberg L, Kriston L, Thomasius R. The Prevalence and Psychosocial Correlates of Internet Gaming Disorder. *Dtsch.* 2017c;114(25):419-424.
143. Wartberg L, Kriston L, Zieglmeier M, Lincoln T, Kammerl R. A longitudinal study on psychosocial causes and consequences of Internet gaming disorder in adolescence. *Psychol Med.* 2018:1-8.
144. Wichstrom L, Stenseng F, Belsky J, von Soest T, Hygen BW. Symptoms of Internet Gaming Disorder in Youth: Predictors and Comorbidity. *J Abnorm Child Psychol.* 2018;05:05.
145. Wu AM, Lai MH, Yu S, Lau JT, Lei MW. Motives for online gaming questionnaire: Its psychometric properties and correlation with Internet gaming disorder symptoms among Chinese people. *J Behav Addict.* 2017a;6(1):11-20.
146. Wu AMS, Chen JH, Tong KK, Yu S, Lau JTF. Prevalence and associated factors of Internet gaming disorder among community dwelling adults in Macao, China. *J Behav Addict.* 2018a;7(1):62-69.
147. Wu TY, Lin CY, Arestedt K, Griffiths MD, Brostrom A, Pakpour AH. Psychometric validation of the Persian nine-item Internet Gaming Disorder Scale - Short Form: Does gender and hours spent online gaming affect the interpretations of item descriptions? *J Behav Addict.* 2017b;6(2):256-263.
148. Wu Y, Sescousse G, Yu H, Clark L, Li H. Cognitive distortions and gambling near-misses in Internet Gaming Disorder: A preliminary study. *PLoS One.* 2018b;13(1):e0191110.
149. Yao YW, Chen PR, Li CR, Hare TA, Li S, Zhang JT et al. Combined reality therapy and mindfulness meditation decrease intertemporal decisional impulsivity in young adults with Internet gaming disorder. *Comput Human Behav.* 2017;68:210-216.
150. Yeh YC, Wang PW, Huang MF, Lin PC, Chen CS, Ko CH. The procrastination of Internet gaming disorder in young adults: The clinical severity. *Psychiatry Res.* 2017;254:258-262.
151. Yen JY, Liu TL, Wang PW, Chen CS, Yen CF, Ko CH. Association between Internet gaming disorder and adult attention deficit and hyperactivity disorder and their correlates: Impulsivity and hostility. *Addict Behav.* 2017a;64:308-313.
152. Yen JY, Yeh YC, Wang PW, Liu TL, Chen YY, Ko CH. Emotional Regulation in Young Adults with Internet Gaming Disorder. *International Journal of Environmental Research & Public Health [Electronic Resource].* 2017b;15(1):25.
153. Yip SW, Gross JJ, Chawla M, Ma SS, Shi XH, Liu L et al. Is Neural Processing of Negative Stimuli Altered in Addiction Independent of Drug Effects? Findings From Drug-Naive Youth with Internet Gaming Disorder. *Neuropsychopharmacology.* 2018;43(6):1364-1372.
154. Youh J, Hong JS, Han DH, Chung US, Min KJ, Lee YS et al. Comparison of Electroencephalography (EEG) Coherence between Major Depressive Disorder (MDD) without Comorbidity and MDD Comorbid with Internet Gaming Disorder. *J Korean Med Sci.* 2017;32(7):1160-1165.
155. Yu H, Cho J. Prevalence of Internet Gaming Disorder among Korean Adolescents and Associations with Non-psychotic Psychological Symptoms, and Physical Aggression. *Am J Health Behav.* 2016;40(6):705-716.

156. Yu S, Mao S, Wu AMS. The interplay among stress, frustration tolerance, mindfulness, and social support in Internet gaming disorder symptoms among Chinese working adults. *Asia-Pac Psychiatry.* 2018;10(4):e12319.
157. Yuan K, Yu D, Cai C, et al. Frontostriatal circuits, resting state functional connectivity and cognitive control in internet gaming disorder. Addict Biol. 2017;22(3):813-822.
158. Zhai J, Luo L, Qiu L, Kang Y, Liu B, Yu D et al. The topological organization of white matter network in internet gaming disorder individuals. *Brain Imaging Behav.* 2017;11(6):1769-1778.
159. Zhang Y, Lin X, Zhou H, Xu J, Du X, Dong G. Brain Activity toward Gaming-Related Cues in Internet Gaming Disorder during an Addiction Stroop Task. *Front Psychol.* 2016;7:714.
160. Weinstein N, Przybylski AK, Murayama K. A prospective study of the motivational and health dynamics of Internet Gaming Disorder. *PeerJ.* 2017;5:e3838.

Appendix E. Study and population characteristics for studies with general or clinical populations, by WHO region

Study	Country	Study Design	Study Population Description	*N*	Age (years) *M (SD) or M (range)*	Gender/ sex (%) *(Male/ female/ other)*	IGD Reporting Method	IGD Measure/ Instrument/ Assessment	IGD Num/Den	IGD Prevalence (%) *Prevalence (95% CI)*	IGD Score *M (SD)*
Eastern Mediterranean Region – General Population											
Hawi 2018[1]	Lebanon	Cross-sectional	High school students 15 - 19y	524	16.2 (1.0)	NR/48/NR	Self-report	IGD-20 Test	NR	9.20 (NR)	NR (NR)
Wu 2017b[2]	Iran	Cross-sectional	Adolescent students 12-19y	2363	15.6 (1.2)	NR/65/NR	Self-report	IGDS9-SF	NR	NR (NR)	24.0 (7.1)
European Region – General Population											
Arcelus 2017[3]	United Kingdom	Cross-sectional	Transgender gamers	245	27.41 (12.4)	45/35/11*	Self-report	IGDS9-SF	2/245	0.70 (NR)	NR (NR)
Bouna-Pyrrou 2015[4]	Germany	Cross-sectional	Male and female volunteers	2465	NR (NR)	62/38/0*	Self-report	DSM-5 questionnaire - German	27/2465	1.10 (NR)	NR (NR)
Buiza-Aguado 2018[5]	Spain	Cross-sectional	Students 12-18y from Málaga	708	15.6 (2.7)	44/56/0	Self-report	IGD Scale - 9 item dichotomous	59/708	8.30 (NR)	2.1 (1.8)
Deleuze 2017[6]	Belgium	Observational	Adult gamers 18-39y	97	22.21 (3.73)	NR/87/NR	Self-report	DSM-5 criteria for IGD	32/97	33.00 (NR)	NR (NR)
Evren 2018[7]	Turkey	Cross-sectional	University students and gamers 15-48y	1250	21.84 (3.42)	56/44/0	Self-report	IGDS9-SF	12/1250	0.96 (NR)	15.50 (6.89)
Jeromin 2016a[8]	Germany	Observational	Adults	87	NR (NR)	0/100/0	Self-report	IGDQ - German	29/686	5.30 (NR)	NR (NR)
Jeromin 2016b[9]	Germany	Cross-sectional	Adult gamers 18-75y	894	26.49 (8.46 NR)	13/87/0	Self-report	IGDQ - German	71/894	7.94 (1.86π)	1.70 (1.86π)

**Study**	**Country**	**Study Design**	**Study Population Description**	***N***	**Age (years)** ***M (SD) or M (range)***	**Gender/ sex (%)** ***(Male/ female/ other)***	**IGD Reporting Method**	**IGD Measure/ Instrument/ Assessment**	**IGD Num/Den**	**IGD Prevalence (%)** ***Prevalence (95% CI)***	**IGD Score** ***M (SD)***
Király 2017[10]	Hungary	Cross-sectional	Gamers 14-64y	4887	22.2 (6.4)	NR/93/NR	Self-report	IGDT-10	138/4887	2.90 (NR)	0.83 (1.36)
Lemmens 2016[11]	Netherlands	Cross-sectional	Adults and adolescents 13-40y	2444	NR (13 to 40)	51/NR/NR	Self-report	IGD Scale - 9 item dichotomous	73/1251	5.80 (NR)	1.17 (1.91)
								IGD Scale - polytomous	NR	NR (NR)	0.17 (1.03)
Lemmens 2015[12]	Netherlands	Cross-sectional	Adults and adolescents 13-40y	2444	NR (13 to 40)	51/NR/NR	Self-report	IGD Scale - 9 item dichotomous	NR	5.40 (NR)	NR (NR)
					NR (13 to 40)	51/NR/NR	Self-report	IGD Scale - 9 item dichotomous	51/1247	4.00 (NR)	NR (NR)
					Ages 31-40: 35.9 (2.8)	51/NR/NR	Self-report	IGD Scale - 9 item dichotomous	NR	5.50 (NR)	NR (NR)
					Ages 21-30: 25.1 (2.8)	51/NR/NR	Self-report	IGD Scale - 9 item dichotomous	NR	6.70 (NR)	NR (NR)
					Ages 13-20: 17.6 (2.2)	51/NR/NR	Self-report	IGD Scale - 9 item dichotomous	NR	3.90 (NR)	NR (NR)
					Ages 31-40: 35.9 (2.8)	51/NR/NR	Self-report	IGD Scale - dichotomous	NR	NR (NR)	4.48 (5.14)
					Ages 21-30: 25.1 (2.8)	51/NR/NR	Self-report	IGD Scale - dichotomous	NR	NR (NR)	4.63 (5.90)
					Ages 13-20: 17.6 (2.2)	51/NR/NR	Self-report	IGD Scale - dichotomous	NR	NR (NR)	3.22 (5.07)
					Ages 31-40: 35.9 (2.8)	51/NR/NR	Self-report	IGD Scale - polytomous	NR	NR (NR)	1.57 (0.87)

Study	Country	Study Design	Study Population Description	*N*	Age (years)<br><br>*M (SD) or M (range)*	Gender/ sex (%)<br><br>*(Male/ female/ other)*	IGD Reporting Method	IGD Measure/ Instrument/ Assessment	IGD Num/Den	IGD Prevalence (%)<br><br>*Prevalence (95% CI)*	IGD Score<br><br>*M (SD)*
					Ages 21-30: 25.1 (2.8)	51/NR/NR	Self-report	IGD Scale - polytomous	NR	NR (NR)	1.61 (0.97)
					Ages 13-20: 17.6 (2.2)	51/NR/NR	Self-report	IGD Scale - polytomous	NR	NR (NR)	1.51 (0.91)
					NR (13 to 40)	51/NR/NR	Self-report	IGD Scale - 27 item dichotomous	NR	NR (NR)	4.20 (5.37)
					NR (13 to 40)	51/NR/NR	Self-report	IGD Scale - 27 item polytomous	NR	NR (NR)	0.58 (0.91)
Monacis 2017[13]	Italy	Cross-sectional	Students	712	21.63 (3.90)	46/54/0*	Self-report	IGDS9-SF - Italian	NR	NR (NR)	15.79 (8.87)
Monacis 2016[14]	Italy	Cross-sectional	Students 16y and older	687	21.62 (3.90)	45/55/0*	Self-report	IGDS9-SF - Italian	NR	NR (NR)	NR (NR)
Monacis 2018[15]	Italy	Cross-sectional	Students and gamers	455	NR (NR)	NR/NR/NR	Self-report	IGDS9-SF - Italian	NR	NR (NR)	14.49 (7.463)
Pontes 2017a[16]	Portugal	Cross-sectional	Students 10-18y in grades 6, 7, 8, and 9	509	13.02 (1.64)	NR/54/NR	Self-report	IGDS9-SF	NR	NR (NR)	15.92 (6.99)
Pontes 2016[17]	Slovenia	Cross-sectional	Students in grade 8 12-16y	1071	13.44 (0.59)	NR/50/NR	Self-report	IGDS9-SF	26/1071	2.60 (1.70 to 3.70)	NR (NR)
Pontes 2017b[18]	United Kingdom	Cross-sectional	Adult gamers	272	41.61 (14.03)	NR/51/NR	Self-report	IGDS9-SF	NR	NR (NR)	14.04 (5.67)
Przybylski 2016a[19]	United Kingdom	Cross-sectional	Adults 18y and older	1899	NR (NR)	50/50/0*	Self-report	DSM-5 criteria for IGD	NR	NR (NR)	NR (NR)
Przybylski 2017b[20]	United Kingdom	Cross-sectional	Adults	1899	NR (NR)	50/50/0*	Self-report	DSM-5 criteria for IGD	NR	0.47 (0.23 to 0.93)	NR (NR)
Rehbein 2015[21]	Germany	Cross-sectional	Students 13-18y in grade 9	11003	14.88 (0.74)	49/51/0*	Self-report	Video Game Dependency Scale	128/11003	1.16 (0.96 to 1.36)	NR (NR)

Study	Country	Study Design	Study Population Description	*N*	Age (years)<br><br>*M (SD) or M (range)*	Gender/ sex (%)<br><br>*(Male/ female/ other)*	IGD Reporting Method	IGD Measure/ Instrument/ Assessment	IGD Num/Den	IGD Prevalence (%)<br><br>*Prevalence (95% CI)*	IGD Score<br><br>*M (SD)*
Thomsen 2018[22]	Denmark	Cross-sectional	People 16-26y	109	21.7 (2.7)	NR/69/NR	Self-report	IGDS9-SF	NR	NR (NR)	9.7 (9.2)
Stavropoulos 2018[23]	United Kingdom	Cross-sectional	American, British, and Australian gamers	281	29.49 (9.47)	NR/86/NR	Self-report	IGDS9-SF	NR	NR (NR)	17.99 (7.02)
Vadlin 2018[24]	Sweden	Observational	Adolescents	1576	13, 15, 16, 18 NR (NR)	58/NR/NR	Self-report	GAIT	NR	NR (NR)	NR (NR)
Wartberg 2017a[25]	Germany	Cross-sectional	Adolescents 12-14y	1095	12.99 (0.82)	49/51/0	Self-report	IGD Scale - 9 item dichotomous	260/1095	23.74* (NR)	NR (NR)
Wartberg 2017b[26]	Germany	Cross-sectional	Adolescents 12-14y	1095	12.99 (0.82)	49/51/0	Self-report	IGD Scale - 9 item dichotomous	260/1095	5.50 (NR)	NR (NR)
Wartberg 2017c[27]	Germany	Cross-sectional	People 12-25y	1531	18.86 (4.06)	49/51/0	Self-report	IGD Scale - 9 item dichotomous	88/1531	5.70 (4.5 to 6.9)	NR (NR)
Wartberg 2018[28]	Germany	Observational	Adolescents 12-14y	1095	*Time 1:* 12.99(0.82)	49/51/0	NR	IGD Scale - 9 item dichotomous	242/985	24.57* (NR)	NR (NR)
					Time 2: 13.89(0.89)	49/51/0	NR	IGD Scale - 9 item dichotomous	257/985	26.09* (NR)	NR (NR)
Wichstrøm 2018[29]	Norway	Observational	Children 10y	740	8, 10 NR (NR)	51/49/0	Health professional	IGDI	14/740	1.70 (0.70 to 2.70)	NR (NR)
De Pasquale 2018[30]	Italy	Cross-sectional	Students 18-25y	221	21.56 (1.42)	58/42/0*	Interview, not specified	IGDS9-SF	33/221	14.90 (NR)	NR (NR)

Study	Country	Study Design	Study Population Description	*N*	Age (years)<br><br>*M (SD) or M (range)*	Gender/ sex (%)<br><br>*(Male/ female/ other)*	IGD Reporting Method	IGD Measure/ Instrument/ Assessment	IGD Num/Den	IGD Prevalence (%)<br><br>*Prevalence (95% CI)*	IGD Score<br><br>*M (SD)*
Evren 2017[31]	Turkey	Cross-sectional	People	457	NR (NR)	62/38/0	Self-report	*Baseline*: IGD Scale - 27 item polytomous - Turkish	NR	NR (NR)	15.61 (22.74)
								Retest: IGD Scale - 27 item polytomous - Turkish	NR	NR (NR)	16.51 (22.50)
								Baseline: IGD Scale - 9 item polytomous - Turkish	19/457	4.20 (NR)	5.39 (8.06)
								Retest: IGD Scale - 9 item polytomous - Turkish	42/457	9.20 (NR)	5.55 (7.69)
Fuster 2016[32]	Spain	Cross-sectional	Gamers 12-58y	1074	26.14 (6.1)	NR/95/NR	Self-report	IGD-20 Test - Spanish	NR	NR (NR)	44.08 (13.19)
Gunuc 2015[33]	Turkey	Cross-sectional	Adolescents 15-18y	131	NR (15 to 18)	NR/NR/NR	Self-report	VGAQ	21/131	16.00 (NR)	21.435 (6.437)
Laconi 2017[34]	France	Cross-sectional	Adult gamers 18-30y	418	21.9 (3)	49/51/0	Self-report	IGDT-10	8/418	1.90 (NR)	28.3 (5.3)
Lopez-Fernandez 2014[35]	Spain	Cross-sectional	Adolescent students 11-18y	1996	NR (11 to 18)	41/58/NR*	Self-report	PVP Scale	81/1047	7.74* (NR)	1.74 (1.70)
	Great Britain	Cross-sectional	Adolescent students 11-18y	1996	NR (NR)	41/58/NR*	Self-report	PVP Scale	179/949	18.86* (NR)	2.47 (2.33)

Study	Country	Study Design	Study Population Description	*N*	Age (years)<br><br>*M (SD) or M (range)*	Gender/ sex (%)<br><br>*(Male/ female/ other)*	IGD Reporting Method	IGD Measure/ Instrument/ Assessment	IGD Num/Den	IGD Prevalence (%)<br><br>*Prevalence (95% CI)*	IGD Score<br><br>*M (SD)*
Milani 2018[36]	Italy	Cross-sectional	Students 9-19y	612	13.94 (2.44)	53/47/0	Self-report	VGA questionnaire (revised)	13/612	2.10 (NR)	NR (NR)
de Palo 2018[37]	Albania	Cross-sectional	People 14-70y	1411	31.38 (10.97)	36/NR/NR	Self-report	IGDS9-SF	NR	NR (NR)	21.1 (7.08)
	United Kingdom	Cross-sectional	People 14-70y	1411	29.50 (9.48)	36/NR/NR	Self-report	IGDS9-SF	NR	NR (NR)	17.99 (7.02)
	Italy	Cross-sectional	People 14-70y	1411	21.62 (3.9)	36/NR/NR	Self-report	IGDS9-SF	NR	NR (NR)	15.93 (8.96)
Peeters 2018[38]	Netherlands	Observational	Adolescents 11-15y	544	13.9 (0.74)	NR/49/NR	Self-report	IGD Scale - 9 item dichotomous	NR	NR (NR)	*Time 1:* 0.686 (1.348)
											Time 2: 0.738 (1.453)
Taquet 2017[39]	France	Cross-sectional	Adult gamers 18-53y	124	25.274 (7.424)	15/85/0*	Self-report	PVP Scale	NR	NR (NR)	4.226 (1.878)
Tejeiro 2016[40]	Spain	Cross-sectional	Adolescent students 11-17y and adult students 20-50y	909	NR (NR)	50/50/0	Self-report	PVP Scale	NR	NR (NR)	*Adolescents*: 1.67 (1.55)
											*Adults:*0.96 (1.31)
Triberti 2018[41]	Italy	Cross-sectional	Gamers 12-47y	133	24.93 (5.31)	17/83/0	Self-report	PVP Scale	NR	NR (NR)	2.84 (1.96)
Region of the Americas – General Population											
Pontes 2017b[18]	USA	Cross-sectional	Adult gamers	405	32.57 (11.33)	NR/62/NR	Self-report	IGDS9-SF	NR	NR (NR)	18.06 (7.36)
Przybylski 2017a[42]	USA	Cross-sectional	Adults 18-24y	1247	NR (18 to 24)	42/58/0*	Self-report	DSM-5 criteria for IGD	NR	1.04 (0.58 to 1.83)	NR (NR)
Przybylski 2017d[43]	USA	Cross-sectional	Adults	5777	46.59 (17.80)	58/42/0*	Self-report	DSM-5 criteria for IGD	NR	0.32 (0.18 to 0.56)	NR (NR)

**Study**	**Country**	**Study Design**	**Study Population Description**	***N***	**Age (years)**<br>***M (SD) or M (range)***	**Gender/ sex (%)**<br>***(Male/ female/ other)***	**IGD Reporting Method**	**IGD Measure/ Instrument/ Assessment**	**IGD Num/Den**	**IGD Prevalence (%)**<br>***Prevalence (95% CI)***	**IGD Score**<br>***M (SD)***
Snodgrass 2018[44]	USA	Cross-sectional	Gamers	58	23.79 (4.15)	NR/64/NR	Self-report	IGDS9-SF	NR	NR (NR)	19.82 (7.57)
									Scale cut-off of ≥ 28: 8/56	14.29* (NR)	37 (1.00)
									Scale cut-off of ≥ 36: 3/56	5.36* (NR)	33.8 (3.85)
Stavropoulos 2018[23]	USA	Cross-sectional	American, British, and Australian gamers	463	25.23 (2.76)	NR/58/NR	Self-report	IGDS9-SF	NR	NR (NR)	20.82 (7.85)
Stockdale 2018[45]	USA	Cross-sectional	Adult university student gamers	174	NR (NR)	NR/NR/NR	Self-report	IGD Scale - 9 item	87/1205	7.22* (NR)	NR (NR)
Stubblefield 2017[46]	USA	Cross-sectional	Children 11-17y	454	13.7 (1.9)	53/47/NR	Self-report	Problem gaming instrument	37/454	8.20 (NR)	NR (NR)
Weinstein 2017[47]	USA	Observational	Adults	2316	NR (NR)	62/38/0*	Health professional	Health professional applying DSM-5 criteria	NR	*Time 1:* 1.49 (1.11 to 2.00)	0.56 (1.13)
										Time 2: 0.99 (0.65 to 1.51)	0.47 (0.97)
Allen 2018[48]	USA	Cross-sectional	Student gamers 18-40y	315	19.34 (2.01)	19/81/0	Self-report	IGD Scale - 27 item polytomous	18/315	5.70 (NR)	0.78 (0.81)
Bargeron 2017[49]	USA	Cross-sectional	Adults	257	21.81 (6.80)	NR/NR/NR	Self-report	DSM-5 criteria for IGD	21/257	8.70 (NR)	1.92 (1.77)
Carlisle 2017[50]	USA	Cross-sectional	Adult gamers 18-95y	1881	28.27 (18 to 95)	39/59/2	Self-report	IGDT-10	79/1881	4.20 (NR)	1.16 (1.54)
de Palo 2018[37]	USA	Cross-sectional	People 14-70y	1411	29.09 (10.72)	36/NR/NR	Self-report	IGDS9-SF	NR	NR (NR)	17.5 (6.01)

Study	Country	Study Design	Study Population Description	*N*	Age (years)<br><br>*M (SD) or M (range)*	Gender/ sex (%)<br><br>*(Male/ female/ other)*	IGD Reporting Method	IGD Measure/ Instrument/ Assessment	IGD Num/Den	IGD Prevalence (%)<br><br>*Prevalence (95% CI)*	IGD Score<br><br>*M (SD)*
Li 2017a[51]	USA	RCT	Adults	30	25.0 (5.4)	17/80/3.3	Self-report	DSM-5 criteria for IGD	61/159	38.90 (NR)	NR (NR)
Sioni 2017[52]	USA	Cross-sectional	Adult gamers 18-77y	394	34.3 (11.6)	NR/50/NR	Self-report	IGD Scale - 9 item dichotomous	102/394	25.90 (NR)	11.8 (2.7)
South-East Asia Region – General Population											
Pontes 2017b[18]	India	Cross-sectional	Adult gamers	336	30.37 (8.90)	NR/68/NR	Self-report	IGDS9-SF	NR	NR (NR)	25.57 (7.64)
Western Pacific Region – General Population											
Jeong 2018[53]	South Korea	Cross-sectional	Adolescents from the 3rd, 4th, and 7th grades	273	NR (NR)	45/55/0*	Health professional	Health professional applying DSM-5 criteria	45/273	16.48* (NR)	NR (NR)
							Self-report	DSM-5 criteria for IGD	47/273	17.22* (NR)	NR (NR)
							Health professional	Health professional applying DSM-5 criteria	43/834	5.16* (NR)	NR (NR)
Jo 2018[54]	South Korea	Cross-sectional	Adolescent gamers 10-19y	121	14‡(10 to 19)	26/74/0	Health professional	Health professional applying DSM-5 criteria	46/121	38.00 (NR)	NR (NR)
Kim 2016b[55]	South Korea	Cross-sectional	Adult gamers 20-49y	3041	NR (20 to 49)	40.0/60/0	Self-report	DSM-5 criteria for IGD	419/3041	13.80 (NR)	NR (NR)
					NR (20 to 29)	40.0/60/0	Self-report	DSM-5 criteria for IGD	160/1221	13.10* (NR)	NR (NR)
					NR (30 to 39)	40.0/60/0	Self-report	DSM-5 criteria for IGD	175/1215	14.40* (NR)	NR (NR)
					NR (40 to 49)	40.0/60/0	Self-report	DSM-5 criteria for IGD	84/605	13.88* (NR)	NR (NR)

Study	Country	Study Design	Study Population Description	*N*	Age (years)<br>*M (SD) or M (range)*	Gender/ sex (%)<br>*(Male/ female/ other)*	IGD Reporting Method	IGD Measure/ Instrument/ Assessment	IGD Num/Den	IGD Prevalence (%)<br>*Prevalence (95% CI)*	IGD Score<br>*M (SD)*
King 2016[56]	Australia	Cross-sectional	Secondary school students 12y and older	824	14.1 (1.5)	51/49/0*	Self-report	IGD Checklist - 9 item	26/824	3.10 (NR)	NR (NR)
King 2017a[57]	Australia	Cross-sectional	Adult gamers 18-56y	630	25.8 (7.1)	NR/76/NR*	Self-report	IGD Checklist - 9 item	20/630	3.17* (NR)	NR (NR)
Lee 2017b[58]	South Korea	Cross-sectional	First year middle school students	330	NR (NR)	51/49/0	Health professional	Health professional applying DSM-5 criteria	16/330	4.90 (NR)	NR (NR)
									Author-defined severe cut-off: 4/330	1.21* (NR)	NR (NR)
Na 2017a[59]	South Korea	Cross-sectional	Gamers 20-49y	2923	NR (20 to 49)	29/71/0*	Self-report	DSM-5 criteria for IGD	494/2923	16.90* (NR)	NR (NR)
					NR (20 to 29)	29/71/0*	Self-report	DSM-5 criteria for IGD	180/1147	15.69* (NR)	NR (NR)
					NR (30 to 39)	29/71/0*	Self-report	DSM-5 criteria for IGD	222/1233	18.00* (NR)	NR (NR)
					NR (40 to 49)	29/71/0*	Self-report	DSM-5 criteria for IGD	92/543	16.94* (NR)	NR (NR)
Na 2017b[60]	South Korea	Cross-sectional	Adults 20-49y	1819	NR (20 to 49)	47/53/0	Self-report	DSM-5 criteria for IGD	257/1819	14.13* (NR)	NR (NR)
					NR (20 to 29)	47/53/0	Self-report	DSM-5 criteria for IGD	89/669	13.30* (NR)	NR (NR)
					NR (30 to 39)	47/53/0	Self-report	DSM-5 criteria for IGD	120/804	14.93* (NR)	NR (NR)
					NR (40 to 39)	47/53/0	Self-report	DSM-5 criteria for IGD	48/346	13.87* (NR)	NR (NR)
Paik 2017a[61]	South Korea	Cross-sectional	Adult gamers 20-39y	3058	26.95 (5.859)	NR/NR/NR	Self-report	DSM-5 criteria for IGD	396/3058	12.90 (NR)	NR (NR)

Study	Country	Study Design	Study Population Description	*N*	Age (years) *M (SD) or M (range)*	Gender/ sex (%) *(Male/ female/ other)*	IGD Reporting Method	IGD Measure/ Instrument/ Assessment	IGD Num/Den	IGD Prevalence (%) *Prevalence (95% CI)*	IGD Score *M (SD)*
Pearcy 2017[62]	Australia	Cross-sectional	Gamers 16-60y	404	23.8 (7.2)	30/70/0	Self-report	PIE-9	34/404	8.42* (NR)	NR (NR)
Pearcy 2016[63]	Australia	Cross-sectional	Gamers older than 16y	408	NR (NR)	31/69/NR	Self-report	PIE-9	34/404	8.42* (NR)	NR (NR)
Rao 2017[64]	China	Cross-sectional	Students in grades 7, 8, 9, and 10	2590	14.1 (NR)	40/60/NR*	Self-report	IGDS9-SF	NR	NR (NR)	NR (NR)
Rho 2017[65]	South Korea	Cross-sectional	Adult gamers 20-49y	3568	NR (20 to 49)	43/57/0	Self-report	DSM-5 criteria for IGD	481/3568	13.50 (NR)	NR (NR)
					NR (20 to 29)	43/57/0	Self-report	DSM-5 criteria for IGD	170/1259	13.50* (NR)	NR (NR)
					NR (30 to 39)	43/57/0	Self-report	DSM-5 criteria for IGD	215/1559	13.79* (NR)	NR (NR)
					NR (40 to 49)	43/57/0	Self-report	DSM-5 criteria for IGD	96/750	12.80* (NR)	NR (NR)
Sigerson 2017[66]	China	Cross-sectional	Adult gamers 18-60y	502	37.1 (13.3)	50/50/0*	Self-report	C-IGDS	NR	NR (NR)	1.29 (1.73)
Stavropoulos 2018[23]	Australia	Cross-sectional	American, British, and Australian gamers	171	25.72 (5.52)	NR/77/NR	Self-report	IGDS9-SF	NR	NR (NR)	18.9 (7.63)
Subramaniam 2016[67]	Singapore	Cross-sectional	Internet users 13-20y	1236	23.7 (5.3)	45/55/0	Self-report	DSM-5 criteria for IGD	172/972	17.71 (NR)	NR (NR)
Wang 2018a[68]	South Korea	Cross-sectional	People 14-39y	7200	NR (14 to 39)	56/44/0	Self-report	IGD-9 Scale	774/7200	10.80 (NR)	6.24 (1.21)
Wu 2017a[69]	China, Taiwan	Cross-sectional	Adult online gamers 18-82y	383	23.7 (6.7)	45/55/0	Self-report	DSM-5 criteria for IGD	64/383	16.71* (NR)	NR (NR)
Wu 2018a[70]	China	Cross-sectional	Adults 18-97y	1000	40.0 (15.3)	56/44/0	Self-report	DSM-5 criteria for IGD	20/1000	2.00 (1.10 to 2.90)	NR (NR)

Study	Country	Study Design	Study Population Description	*N*	Age (years) *M (SD) or M (range)*	Gender/ sex (%) *(Male/ female/ other)*	IGD Reporting Method	IGD Measure/ Instrument/ Assessment	IGD Num/Den	IGD Prevalence (%) *Prevalence (95% CI)*	IGD Score *M (SD)*
Yu 2016[71]	South Korea	Cross-sectional	Middle school students 13-15y	2014	14.5 (0.50)	50/51/NR*	Self-report	DSM-5 criteria for IGD	119/2014	5.90 (NR)	16.63 (7.6)
Yu 2018[72]	China	Cross-sectional	Adult gamers 18-67y	327	31.93 (9.04)	NR/79/NR	Self-report	DSM-5 criteria for IGD	NR	2.30 (NR)	NR (NR)
Adams 2018[73]	Australia	Observational	Adult gamers 18-29y	125	23.34 (3.39)	25/75/0	Self-report	IGDS9-SF	NR	NR (NR)	*Time 1:* 19.48 (7.07)
											Time 2: 18.67 (6.86)
											Time 3: 17.78 (5.80)
Burleigh 2018[74]	Australia	Observational	Adult gamers 18-29y	125	23.02 (3.43)	25/75/0	Self-report	IGDS9-SF	NR	NR (NR)	*Time 1:* 19.48 (7.06)
											Time 2: 18.67 (6.86)
											Time 3: 17.78 (SD 5.80)
Fu 2015[75]	China	Cross-sectional	Students 14-23y	700	17.0 (1.25)	52/48/0	Self-report	Internet Gaming Addiction Scale	NR	NR (NR)	NR (NR)
King 2017c[76]	Australia	Cross-sectional	Students 12-17y	824	14.1 (1.5)	51/49/NR*	Self-report	IGD Checklist - 12 item	25/799	3.10 (NR)	NR (NR)
Liew 2018[77]	Australia	Observational	Adult gamers 18-29y	125	NR (18 to 29)	25/75/0	Self-report	IGDS9-SF	NR	NR (NR)	*Time 1:* 19.44 (7.07)
											Time 2: 18.8 (6.91)
											Time 3: 17.95 (5.93)

Study	Country	Study Design	Study Population Description	*N*	Age (years)<br><br>*M (SD) or M (range)*	Gender/ sex (%)<br><br>*(Male/ female/ other)*	IGD Reporting Method	IGD Measure/ Instrument/ Assessment	IGD Num/Den	IGD Prevalence (%)<br><br>*Prevalence (95% CI)*	IGD Score<br><br>*M (SD)*
Schneider 2018[78]	Australia	Cross-sectional	Adolescents 12-19y	823	14.2 (1.4)	51/49/0*	Self-report	IGD Checklist - 12 item	26/823	3.20 (NR)	NR (NR)
International – General Population											
Pontes 2014[79]	Canada, Finland, Germany, the Netherlands, Sweden, United Kingdom, USA, Other countries	Cross-sectional	Gamers 16-58y	1003	26.5 (0.26)	NR/85/NR	Self-report	DSM-5 criteria for IGD	53/1003	5.28* (NR)	NR (NR)
Przybylski 2016b[80]	Canada, Germany, United Kingdom, USA	Cross-sectional	Adults 18-24y	10009	NR (18 to 24)	50/50/0*	Self-report	DSM-5 criteria for IGD	NR	NR (NR)	NR (NR)
Przybylski 2017c[81]	Canada, Germany, United Kingdom, USA	Cross-sectional	Adults 18-24y	10099	NR (18 to 24)	49/50/NR*	Self-report	DSM-5 criteria for IGD	NR	0.68 (0.53 to 0.87)	NR (NR)
Pontes 2015[82]	Germany, Netherland, Sweden, United Kingdom, USA, Other countries	Cross-sectional	Gamers 16-70y	1060	27.3 (9.02)	NR/85/NR	Self-report	IGDS9-SF	NR	NR (NR)	18 (6.63)

Study	Country	Study Design	Study Population Description	*N*	Age (years)	Gender/ sex (%)	IGD Reporting Method	IGD Measure/ Instrument/ Assessment	IGD Num/Den	IGD Prevalence (%)	IGD Score
					M (SD) or M (range)	*(Male/ female/ other)*				*Prevalence (95% CI)*	*M (SD)*
European Region – Clinical Population											
Evren 2017[31]	Turkey	Cross-sectional	People	457	NR (NR)	62/38/0	Self-report	IGD Scale - 27 item polytomous - Turkish	NR	NR (NR)	91.74 (10.34)
								IGD Scale - 9 item polytomous - Turkish	NR	NR (NR)	33.32 (3.25)
Krossbakken 2018[83]	Norway	RCT	Guardians of children 8-12y	1657	10.1 (NR)	46/53/NR*	Parent report	DSM-5 criteria for IGD	NR	NR (NR)	NR (NR)
Mallorquí-Bagué 2017[84]	Spain	Cross-sectional	Adults with IGD or online gambling disorder	288	NR (NR)	5/95/0*	Health professional	Health professional	27/288	9.38* (NR)	NR (NR)
Region of the Americas – Clinical Population											
Sanders 2017[85]	Canada	Cross-sectional	Adults	542	π	39/61/7	Self-report	DSM-5 criteria for IGD	39/1238	3.20 (NR)	NR (NR)
Western Pacific Region – Clinical Population											
Cai 2016[86]	China	Observational	Adult gamers with IGD	57	NR (NR)	NR/79/NR*	Self-report	DSM-5 criteria for IGD	NR	NR (NR)	NR (NR)
Dong 2017c[87]	China	Observational	University student gamers from Shanghai with IGD or RGU; normal or corrected-to-normal vision	70	NR (NR)	0/100/0	NR	DSM-5 criteria for IGD	NR	NR (NR)	5.89 (1.12)

**Study**	**Country**	**Study Design**	**Study Population Description**	***N***	**Age (years)** *M (SD) or M (range)*	**Gender/ sex (%)** *(Male/ female/ other)*	**IGD Reporting Method**	**IGD Measure/ Instrument/ Assessment**	**IGD Num/Den**	**IGD Prevalence (%)** *Prevalence (95% CI)*	**IGD Score** *M (SD)*
Dong 2018a[88]	China	Cross-sectional	University student gamers with IGD or recreational Internet game use	86	NR (NR)	NR/NR/NR	NR	DSM-5 criteria for IGD	NR	NR (NR)	5.92 (1.14)
Kaptsis 2016[89]	Australia	Uncontrolled before-after	Adult gamers 18-25y	24	24.6 (5.1)	17/83/0*	Self-report	IGD Checklist - 9 item	NR	NR (NR)	5.89π (0.86)
Kim 2017a[90]	South Korea	Observational	Participants with IGD or OCD	77	NR (NR)	21/79/0*	Health professional	Health professional applying DSM-5 criteria	NR	NR (NR)	NR (NR)
Kim 2017c[91]	South Korea	Observational	Participants with IGD, AUD, or OCD	225	NR (NR)	10/90/0*	Health professional	Health professional applying DSM-5 criteria	NR	NR (NR)	NR (NR)
King 2018a[92]	Australia	Quasi-experimental	Adult gamers 18-48y with gaming problems	186	23.4 (5.2)	NR/95/NR	Self-report	IGD Checklist - 9 item	NR	NR (NR)	NR (NR)
King 2017b[93]	Australia	Uncontrolled before-after	Adult gamers 18-36y	24	24.6 (5.1)	17/83/0*	Self-report	IGD Checklist - 9 item	9/24	37.50* (NR)	NR (NR)
Ko 2017[94]	Taiwan	Observational	Gamers 20-30y with IGD	174	NR (20 to 30)	NR/NR/NR	Health professional	Health professional applying DSM-5 criteria	NR	NR (NR)	NR (NR)
Ko 2014[95]	Taiwan	Cross-sectional	Gamers 20-30y with IGD or in IGD remission	225	NR (NR)	16/84/0	Health professional	Health professional applying DSM-5 criteria	NR	NR (NR)	NR (NR)

Study	Country	Study Design	Study Population Description	*N*	Age (years)<br>*M (SD) or M (range)*	Gender/ sex (%)<br>*(Male/ female/ other)*	IGD Reporting Method	IGD Measure/ Instrument/ Assessment	IGD Num/Den	IGD Prevalence (%)<br>*Prevalence (95% CI)*	IGD Score<br>*M (SD)*
Koo 2017[96]	South Korea	Cross-sectional	Adolescents (middle school students)	236	13.61 (0.87)	29/71/0	Health professional	SCI-IGD	27/236	11.44* (NR)	NR (NR)
Lee 2016[97]	South Korea	Observational	Adult gamers with IGD	48	NR (NR)	NR/NR/NR	Health professional	Health professional applying DSM-5 criteria	NR	NR (NR)	NR (NR)
Lee 2018c[98]	South Korea	Case-control	Adolescents 12-18y with IGD	87	NR (12 to 18)	NR/NR/NR	Self-report	DSM-5 criteria for IGD	251/3166	7.93* (NR)	NR (NR)
Li 2018a[99]	China	Observational	Primary and secondary school students 8-15y	241	12.09 (1.41)	43/NR/NR	Self-report	K-scale - Korean Internet Addiction Scale for Adolescents	NR	NR (NR)	*Time 1:* 31.33 (6.38)<br>*Time 2:* 30.92 (6.87)
Paik 2017b[100]	South Korea	Case-control	Adults 19-47y with IGD	150	30.09 (6.343)	NR/77/NR	NR	DSM-5 criteria for IGD	63/150	42.00* (NR)	NR (NR)
Park 2016b[101]	South Korea	Observational	Gamers with IGD	49	NR (NR)	18/82/0*	Health professional	Health professional applying DSM-5 criteria	NR	NR (NR)	NR (NR)
Rho 2016[102]	South Korea	Cross-sectional	Adult gamers 20-49y	1022	NR (20 to 49)	40/60/0	Self-report	DSM-5 criteria for IGD	511/3881	13.17* (NR)	NR (NR)
Ryu 2018[103]	South Korea	Cross-sectional	Young adults with IGD	123	24.92 (4.71)	NR/79/NR	Health professional	Health professional applying DSM-5 criteria	NR	NR (NR)	NR (NR)
Wang 2017a[104]	Taiwan	Cross-sectional	Adult gamers 20-30y with IGD	383	NR (20 to 30)	45/55/0	Health professional	Health professional applying DSM-5 criteria	NR	NR (NR)	NR (NR)

Study	Country	Study Design	Study Population Description	*N*	Age (years)<br><br>*M (SD) or M (range)*	Gender/ sex (%)<br><br>*(Male/ female/ other)*	IGD Reporting Method	IGD Measure/ Instrument/ Assessment	IGD Num/Den	IGD Prevalence (%)<br><br>*Prevalence (95% CI)*	IGD Score<br><br>*M (SD)*
Wang 2017b[105]	China	Observational	Gamers with IGD or recreational Internet game use	70	NR (NR)	10/NR/NR	NR	DSM-5 criteria for IGD	NR	NR (NR)	5.8 (1.10)
Wang 2018c[106]	China	Cross-sectional	University student gamers with IGD or RGU	104	NR (NR)	NR/62/NR*	NR	DSM-5 criteria for IGD	NR	NR (NR)	5.86 (1.04)
Yeh 2017[107]	Taiwan	Cross-sectional	Gamers 20-30y with IGD	174	NR (20 to 30)	20/80/0*	Health professional	Health professional applying DSM-5 criteria	NR	NR (NR)	NR (NR)
Yen 2017a[108]	Taiwan	Cross-sectional	Gamers 20-30y with IGD	174	NR (20 to 30)	20/80/0*	Health professional	Health professional applying DSM-5 criteria	NR	NR (NR)	NR (NR)
Yen 2017b[109]	Taiwan	Cross-sectional	Gamers 20-30y with IGD	174	NR (20 to 30)	20/80/0*	Health professional	Health professional applying DSM-5 criteria	NR	NR (NR)	NR (NR)
Yuan 2017[110]	China	Observational	Student gamers with IGD	87	NR (NR)	24/76/0*	Self-report	DSM-5 criteria for IGD	NR	NR (NR)	NR (NR)
Zhai 2017[111]	China	Observational	Gamers with IGD	32	NR (NR)	NR/69/NR*	NR	DSM-5 criteria for IGD	NR	NR (NR)	NR (NR)
King 2018b[112]	Australia	Cross-sectional	Adult gamers 18-56y	630	NR (NR)	NR/76/NR*	Self-report	IGD Scale - 9 item	20/630	4.00 (NR)	NR (NR)

Study	Country	Study Design	Study Population Description	*N*	Age (years)<br><br>*M (SD) or M (range)*	Gender/ sex (%)<br><br>*(Male/ female/ other)*	IGD Reporting Method	IGD Measure/ Instrument/ Assessment	IGD Num/Den	IGD Prevalence (%)<br><br>*Prevalence (95% CI)*	IGD Score<br><br>*M (SD)*
Tian 2018[113]	China	Observational	Adolescents with IGD	88	NR (NR)	45/49/NR*	Health professional	Health professional applying DSM-5 criteria	NR	NR (NR)	NR (NR)

Note: No data found for African region; IGD prevalence values were charted to two decimal places. All other values are as reported by authors.
***** Calculated using raw data provided in the study; **‡** Median; **π** Unclear

CI: *Confidence Interval*; **C-IGDS**: *Chinese Internet Gaming Disorder Scale;* **GAIT**: *The Gaming Addiction Identification Test;* **IGD**: *Internet Gaming Disorder*; **IGDI**: *Internet Gaming Disorder Interview;* **IGDQ**: *Internet Gaming Disorder Questionnaire;* **IGDS9-SF**: *Internet Gaming Disorder Scale - Short Form;* **IGDT-10**: *Ten-Item Internet Gaming Disorder Test*; **M**: *Mean*; **N**: *Sample size*; **NR**: *Data not reported in the study*; **Num/Den**: *Prevalence numerator/Prevalence Denominator*; **PIE-9**: *Personal Internet Gaming Disorder Evaluation*; **PVP Scale**: *Problematic Videogame Playing Scale*; **SCI-IGD**: *Structured Clinical Interview for Internet Gaming Disorder*; **SD**: *Standard Deviation;* **USA**: *United States of America;* **VGA**: *Video Game Addiction;* **VGAQ**: *Video Game Addiction Questionnaire*

Ranges for measures used: **C-IGDS**: *0-9**; **DSM-5 criteria for IGD**: *0-9*; **IGD Checklist - 9 item**: *No lower or upper limit reported*; **IGD Scale - 9 item dichotomous**: *0-9*; **IGD Scale - 27 item dichotomous**: *0-27*; **IGD Scale - 27 item polytomous**: *0-135**; **IGD Scale - 27 item polytomous - Turkish**: *0-135**
IGD Scale - 9 item polytomous - Turkish: *0-45**; **IGD Scale - dichotomous**: *No lower or upper limit reported*; **IGD Scale - polytomous**: *No lower or upper limit reported*; **IGD-20 Test - Spanish**: *20-100**; **IGD-9 Scale**: *0-9*; **IGDQ - German**: *0-9**; **IGDS9-SF**: *9-45*; **IGDS9-SF - Italian**: *9-45**; **IGDT-10**: *0-9*; **K-scale - Korean Internet Addiction Scale for Adolescents**: *20-80*; **PVP Scale**: *0-9*; **VGAQ**: *9-45**

Appendix F. Study and population characteristics for studies with general or clinical populations, by gender/sex

Study	Country	Study Design	Study Population Description	*N*	Age (years)<br>*M (SD) or M (range)*	IGD Reporting Method	IGD Measure/ Instrument/ Assessment	IGD Num/Den	IGD Prevalence (%)<br>*Prevalence (95% CI)*	IGD Score<br>*M (SD)*
Males – General Population										
Bouna-Pyrrou 2015[4]	Germany	Cross-sectional	Male and female volunteers	2465	30‡ (24 to 43)^	Self-report	DSM-5 questionnaire - German	17/941	1.80 (NR)	NR (NR)
Buiza-Aguado 2018[5]	Spain	Cross-sectional	Students 12-18y from Málaga	708	NR (NR)	Self-report	IGD Scale - 9 item dichotomous	51/394	12.90 (NR)	2.5 (1.9)
Evren 2018[7]	Turkey	Cross-sectional	University students and gamers 15-48y	1250	NR (NR)	Self-report	IGDS9-SF	NR	NR (NR)	18.6 (NR 7.43)
Jeromin 2016b[9]	Germany	Cross-sectional	Adult gamers 18-75y	894	NR (NR)	Self-report	IGDQ - German	NR	NR (NR)	1.7 (1.9π)
Király 2017[10]	Hungary	Cross-sectional	Gamers 14-64y	4887	NR (NR)	Self-report	IGDT-10	124/4517	2.75* (NR)	NR (NR)

Study	Country	Study Design	Study Population Description	*N*	Age (years)<br><br>*M (SD) or M (range)*	IGD Reporting Method	IGD Measure/ Instrument/ Assessment	IGD Num/Den	IGD Prevalence (%)<br><br>*Prevalence (95% CI)*	IGD Score<br><br>*M (SD)*
Lemmens 2015[12]	Netherlands	Cross-sectional	Adults and adolescents 13-40y	2444	NR (NR)	Self-report	IGD Scale - 9 item dichotomous	NR	6.80 (NR)	NR (NR)
							IGD Scale - dichotomous	NR	NR (NR)	4.87 (5.88)
							IGD Scale - polytomous	NR	NR (NR)	0.74 (1.02)
Monacis 2017[13]	Italy	Cross-sectional	Students	712	NR (NR)	Self-report	IGDS9-SF - Italian	NR	NR (NR)	18.67 (9.77)
Monacis 2016[14]	Italy	Cross-sectional	Students 16y and older	687	NR (NR)	Self-report	IGDS9-SF - Italian	NR	NR (NR)	18.75 (9.80)
Monacis 2018[15]	Italy	Cross-sectional	Students and gamers	455	NR (NR)	Self-report	IGDS9-SF - Italian	NR	NR (NR)	16.31 (8.22)
Pontes 2017a[16]	Portugal	Cross-sectional	Students 10-18y in grades 6, 7, 8, and 9	509	NR (NR)	Self-report	IGDS9-SF	NR	NR (NR)	18.6 (5.32)
Rehbein 2015[21]	Germany	Cross-sectional	Students 13-18y in grade 9	11003	14.94 (0.75)	Self-report	Video Game Dependency Scale	NR	2.02 (1.65 to 2.38)	NR (NR)
Vadlin 2018[24]	Sweden	Observational	Adolescents	1576	NR (NR)	Self-report	GAIT	NR	NR (NR)	NR (NR)

Study	Country	Study Design	Study Population Description	*N*	Age (years) *M (SD) or M (range)*	IGD Reporting Method	IGD Measure/ Instrument/ Assessment	IGD Num/Den	IGD Prevalence (%) *Prevalence (95% CI)*	IGD Score *M (SD)*
Wartberg 2017c[27]	Germany	Cross-sectional	People 12-25y	1531	NR (NR)	Self-report	IGD Scale - 9 item dichotomous	NR	8.40 (6.40 to 10.4)	NR (NR)
Wichstrøm 2018[29]	Norway	Observational	Children 10y	740	NR (NR)	Health professional	IGDI	NR	3.00 (1.00 to 5.00)	NR (NR)
De Pasquale 2018[30]	Italy	Cross-sectional	Students 18-25y	221	NR (NR)	Interview, not specified	IGDS9-SF	31/93	33.33* (NR)	28.03 (2.21)
Laconi 2017[34]	France	Cross-sectional	Adult gamers 18-30y	418	NR (NR)	Self-report	IGDT-10	7/212	3.30* (NR)	3.6 (3.7)
Lopez-Fernandez 2014[35]	Spain	Cross-sectional	Adolescent students 11-18y	1996	NR (11 to 12)	Self-report	PVP Scale	NR	NR (NR)	2.65 (2.06)
					NR (13 to 15)	Self-report	PVP Scale	NR	NR (NR)	2.17 (1.82)
					NR (16 to 18)	Self-report	PVP Scale	NR	NR (NR)	1.81 (1.70)
Lopez-Fernandez 2014[35]	Great Britain	Cross-sectional	Adolescent students 11-18y	1996	NR (11 to 12)	Self-report	PVP Scale	NR	NR (NR)	3.25 (2.18)
					NR (13 to 15)	Self-report	PVP Scale	NR	NR (NR)	2.8 (2.44)
					NR (16 to 18)	Self-report	PVP Scale	NR	NR (NR)	2.34 (2.74)

Study	Country	Study Design	Study Population Description	*N*	Age (years)<br><br>*M (SD) or M (range)*	IGD Reporting Method	IGD Measure/ Instrument/ Assessment	IGD Num/Den	IGD Prevalence (%)<br><br>*Prevalence (95% CI)*	IGD Score<br><br>*M (SD)*
Tejeiro 2016[40]	Spain	Cross-sectional	Adolescent students 11-17y and adult students 20-50y	909	NR (NR)	Self-report	PVP Scale	NR	NR (NR)	*Adolescents*: 2.15 (1.70)
										Adults: 1.39 (1.58)
Triberti 2018[41]	Italy	Cross-sectional	Gamers 12-47y	133	NR (NR)	Self-report	PVP Scale	22/110	20.00* (NR)	NR (NR)
Przybylski 2017b[20]	United Kingdom	Cross-sectional	Adults	1899	NR (NR)	Self-report	DSM-5 criteria for IGD	NR	0.21 (0.03 to 0.84)	NR (NR)
Przybylski 2017c[81]	Canada, Germany, United Kingdom, USA	Cross-sectional	Adults 18-24y	10099	NR (NR)	Self-report	DSM-5 criteria for IGD	NR	0.80 (0.58 to 1.10)	NR (NR)
Snodgrass 2018[44]	USA	Cross-sectional	Gamers	58	NR (NR)	Self-report	IGDS9-SF	*Scale cut-off of ≥ 28:* 6/36	16.67* (NR)	NR (NR)
								Scale cut-off of ≥ 36: 2/36	5.56* (NR)	NR (NR)
Stubblefield 2017[46]	USA	Cross-sectional	Children 11-17y	454	NR (NR)	Self-report	Problem gaming instrument	27/213	12.70 (NR)	NR (NR)

Study	Country	Study Design	Study Population Description	*N*	Age (years)<br><br>*M (SD) or M (range)*	IGD Reporting Method	IGD Measure/ Instrument/ Assessment	IGD Num/Den	IGD Prevalence (%)<br><br>*Prevalence (95% CI)*	IGD Score<br><br>*M (SD)*
Przybylski 2017a[42]	USA	Cross-sectional	Adults 18-24y	1247	NR (NR)	Self-report	DSM-5 criteria for IGD	NR	0.97 (0.43 to 2.09)	NR (NR)
Przybylski 2017d[43]	USA	Cross-sectional	Adults	5777	NR (NR)	Self-report	DSM-5 criteria for IGD	NR	0.38 (0.17 to 0.85)	NR (NR)
Cho 2017[114]	South Korea	Cross-sectional	Male adult gamers	52	23.7 (2.3 NR)	Health professional	Health professional applying DSM-5 criteria	29/52	55.77* (NR)	NR (NR)
Kim 2016b[55]	South Korea	Cross-sectional	Adult gamers 20-49y	3041	NR (NR)	Self-report	DSM-5 criteria for IGD	238/1824	13.05* (NR)	NR (NR)
Lee 2018a[115]	South Korea	Observational	Males 16-27y	41	23.1 (2.6 NR)	Health professional	Health professional applying DSM-5 criteria	23/40	57.50* (NR)	NR (NR)
Na 2017a[59]	South Korea	Cross-sectional	Gamers 20-49y	2923	NR (NR)	Self-report	DSM-5 criteria for IGD	358/2086	17.16* (NR)	NR (NR)
Na 2017b[60]	South Korea	Cross-sectional	Adults 20-49y	1819	NR (NR)	Self-report	DSM-5 criteria for IGD	150/959	15.64* (NR)	NR (NR)
Paik 2017a[61]	South Korea	Cross-sectional	Adult gamers 20-39y	3058	NR (NR)	Self-report	DSM-5 criteria for IGD	220/1548	14.20 (NR)	NR (NR)
Rho 2017[65]	South Korea	Cross-sectional	Adult gamers 20-49y	3568	NR (NR)	Self-report	DSM-5 criteria for IGD	290/2036	14.24* (NR)	NR (NR)

Study	Country	Study Design	Study Population Description	*N*	Age (years)<br><br>*M (SD) or M (range)*	IGD Reporting Method	IGD Measure/ Instrument/ Assessment	IGD Num/Den	IGD Prevalence (%)<br><br>*Prevalence (95% CI)*	IGD Score<br><br>*M (SD)*
Subramaniam 2016[67]	Singapore	Cross-sectional	Internet users 13-20y	1236	NR (NR)	Self-report	DSM-5 criteria for IGD	114/614	18.57* (NR)	NR (NR)
Yu 2016[71]	South Korea	Cross-sectional	Middle school students 13-15y	2014	NR (NR)	Self-report	DSM-5 criteria for IGD	107/1025	10.40 (NR)	NR (NR)
Females – General Population										
Bouna-Pyrrou 2015[4]	Germany	Cross-sectional	Male and female volunteers	2465	27‡ (23 to 29)^	Self-report	DSM-5 questionnaire - German	10/1524	0.70 (NR)	NR (NR)
Buiza-Aguado 2018[5]	Spain	Cross-sectional	Students 12-18y from Málaga	708	NR (NR)	Self-report	IGD Scale - 9 item dichotomous	8/313	2.50 (NR)	1.1 (1.4)
Evren 2018[7]	Turkey	Cross-sectional	University students and gamers 15-48y	1250	NR (NR)	Self-report	IGDS9-SF	NR	NR (NR)	13.07 (NR 5.29)
Jeromin 2016b[9]	Germany	Cross-sectional	Adult gamers 18-75y	894	NR (NR)	Self-report	IGDQ - German	NR	NR (NR)	1.4 (1.8π)

Study	Country	Study Design	Study Population Description	*N*	Age (years)<br><br>*M (SD) or M (range)*	IGD Reporting Method	IGD Measure/ Instrument/ Assessment	IGD Num/Den	IGD Prevalence (%)<br><br>*Prevalence (95% CI)*	IGD Score<br><br>*M (SD)*
Lemmens 2015[12]	Netherlands	Cross-sectional	Adults and adolescents 13-40y	2444	NR (NR)	Self-report	IGD Scale - 9 item dichotomous	NR	4.00 (NR)	NR (NR)
							IGD Scale - dichotomous	NR	NR (NR)	3.4 (4.60)
							IGD Scale - polytomous	NR	NR (NR)	0.39 (0.72)
Monacis 2017[13]	Italy	Cross-sectional	Students	712	NR (NR)	Self-report	IGDS9-SF - Italian	NR	NR (NR)	12.46 (6.24)
Monacis 2016[14]	Italy	Cross-sectional	Students 16y and older	687	NR (NR)	Self-report	IGDS9-SF - Italian	NR	NR (NR)	12.53 (6.40)
Monacis 2018[15]	Italy	Cross-sectional	Students and gamers	455	NR (NR)	Self-report	IGDS9-SF - Italian	NR	NR (NR)	12.32 (5.75)
Pontes 2017a[16]	Portugal	Cross-sectional	Students 10-18y in grades 6, 7, 8, and 9	509	NR (NR)	Self-report	IGDS9-SF	NR	NR (NR)	12.83 (7.17)
Rehbein 2015[21]	Germany	Cross-sectional	Students 13-18y in grade 9	11003	14.81 (0.71)	Self-report	Video Game Dependency Scale	NR	0.26 (0.12 to 0.40)	NR (NR)
Vadlin 2018[24]	Sweden	Observational	Adolescents	1576	NR (NR)	Self-report	GAIT	NR	NR (NR)	NR (NR)

Study	Country	Study Design	Study Population Description	*N*	Age (years)<br><br>*M (SD) or M (range)*	IGD Reporting Method	IGD Measure/ Instrument/ Assessment	IGD Num/Den	IGD Prevalence (%)<br><br>*Prevalence (95% CI)*	IGD Score<br><br>*M (SD)*
Wartberg 2017c[27]	Germany	Cross-sectional	People 12-25y	1531	NR (NR)	Self-report	IGD Scale - 9 item dichotomous	NR	2.90 (1.70 to 4.10)	NR (NR)
Wichstrøm 2018[29]	Norway	Observational	Children 10y	740	NR (NR)	Health professional	IGDI	NR	0.50 (0.00 to 1.20)	NR (NR)
De Pasquale 2018[30]	Italy	Cross-sectional	Students 18-25y	221	NR (NR)	Interview, not specified	IGDS9-SF	2/128	1.56* (NR)	27.29 (0.76)
Laconi 2017[34]	France	Cross-sectional	Adult gamers 18-30y	418	NR (NR)	Self-report	IGDT-10	1/206	0.49* (NR)	2.4 (2.8)
Lopez-Fernandez 2014[35]	Spain	Cross-sectional	Adolescent students 11-18y	1996	NR (11 to 12)	Self-report	PVP Scale	NR	NR (NR)	1.49 (1.37)
					NR (13 to 15)	Self-report	PVP Scale	NR	NR (NR)	1.44 (1.52)
					NR (16 to 18)	Self-report	PVP Scale	NR	NR (NR)	1.02 (1.87)
Lopez-Fernandez 2014[35]	Great Britain	Cross-sectional	Adolescent students 11-18y	1996	NR (11 to 12)	Self-report	PVP Scale	NR	NR (NR)	2.09 (2.22)
					NR (13 to 15)	Self-report	PVP Scale	NR	NR (NR)	1.76 (1.90)
					NR (16 to 18)	Self-report	PVP Scale	NR	NR (NR)	1.04 (1.86)

Study	Country	Study Design	Study Population Description	*N*	Age (years)<br><br>*M (SD) or M (range)*	IGD Reporting Method	IGD Measure/ Instrument/ Assessment	IGD Num/Den	IGD Prevalence (%)<br><br>*Prevalence (95% CI)*	IGD Score<br><br>*M (SD)*
Tejeiro 2016[40]	Spain	Cross-sectional	Adolescent students 11-17y and adult students 20-50y	909	NR (NR)	Self-report	PVP Scale	NR	NR (NR)	*Adolescents*: 1.17 (1.19)<br><br>*Adults*: 0.73 (1.07)
Triberti 2018[41]	Italy	Cross-sectional	Gamers 12-47y	133	NR (NR)	Self-report	PVP Scale	6/23	26.09* (NR)	NR (NR)
Przybylski 2017b[20]	United Kingdom	Cross-sectional	Adults	1899	NR (NR)	Self-report	DSM-5 criteria for IGD	NR	0.74 (0.32 to 1.60)	NR (NR)
Przybylski 2017c[81]	Canada, Germany, United Kingdom, USA	Cross-sectional	Adults 18-24y	10099	NR (NR)	Self-report	DSM-5 criteria for IGD	NR	0.56 (0.38 to 0.82)	NR (NR)
Stubblefield 2017[46]	USA	Cross-sectional	Children 11-17y	454	NR (NR)	Self-report	Problem gaming instrument	10/240	4.20 (NR)	NR (NR)
Przybylski 2017a[42]	USA	Cross-sectional	Adults 18-24y	1247	NR (NR)	Self-report	DSM-5 criteria for IGD	NR	1.14 (0.46 to 2.59)	NR (NR)
Przybylski 2017d[43]	USA	Cross-sectional	Adults	5777	NR (NR)	Self-report	DSM-5 criteria for IGD	NR	0.25 (0.12 to 0.53)	NR (NR)
Kim 2016b[55]	South Korea	Cross-sectional	Adult gamers 20-49y	3041	NR (NR)	Self-report	DSM-5 criteria for IGD	181/1217	14.87* (NR)	NR (NR)

**Study**	**Country**	**Study Design**	**Study Population Description**	***N***	**Age (years)**<br>*M (SD) or M (range)*	**IGD Reporting Method**	**IGD Measure/ Instrument/ Assessment**	**IGD Num/Den**	**IGD Prevalence (%)**<br>*Prevalence (95% CI)*	**IGD Score**<br>*M (SD)*
Na 2017a[59]	South Korea	Cross-sectional	Gamers 20-49y	2923	NR (NR)	Self-report	DSM-5 criteria for IGD	136/837	16.25* (NR)	NR (NR)
Na 2017b[60]	South Korea	Cross-sectional	Adults 20-49y	1819	NR (NR)	Self-report	DSM-5 criteria for IGD	107/860	12.44* (NR)	NR (NR)
Paik 2017a[61]	South Korea	Cross-sectional	Adult gamers 20-39y	3058	NR (NR)	Self-report	DSM-5 criteria for IGD	176/1510	11.70 (NR)	NR (NR)
Rho 2017[65]	South Korea	Cross-sectional	Adult gamers 20-49y	3568	NR (NR)	Self-report	DSM-5 criteria for IGD	191/1532	12.47* (NR)	NR (NR)
Subramaniam 2016[67]	Singapore	Cross-sectional	Internet users 13-20y	1236	NR (NR)	Self-report	DSM-5 criteria for IGD	58/358	16.20* (NR)	NR (NR)
Yu 2016[71]	South Korea	Cross-sectional	Middle school students 13-15y	2014	NR (NR)	Self-report	DSM-5 criteria for IGD	12/999	1.20 (NR)	NR (NR)
Males – Clinical Population										
Kaess 2017[116]	Germany	Observational	Males 13-25y with IGD	49	NR(NR)	Health professional	Health professional applying DSM-5 criteria	NR	NR (NR)	6.88π (1.42)
van Rooij 2017[117]	Netherlands	Cross-sectional	Male gamers 12-23y who were 'game addicted'	32	17.6 (2.5)	Health professional	C-VAT 2.0	29/32	91.00 (NR)	NR (NR)
						Self-report	VAT	NR	NR (NR)	2.48 (0.6)

Study	Country	Study Design	Study Population Description	*N*	Age (years) *M (SD) or M (range)*	IGD Reporting Method	IGD Measure/ Instrument/ Assessment	IGD Num/Den	IGD Prevalence (%) *Prevalence (95% CI)*	IGD Score *M (SD)*
Bae 2017[118]	South Korea	Cross-sectional	Male adult gamers with IGD, or male adults with ibGD	44	NR (NR)	NR	DSM-5 criteria for IGD	NR	NR (NR)	NR (NR)
Choi 2017[119]	South Korea	Cross-sectional	Male adult gamers	71	NR (NR)	Self-report	IGD Checklist - 9 item	NR	NR (NR)	6.27 (1.55)
Choi 2014[120]	South Korea	Observational	Males with IGD, GD, or AUD	60	NR (NR)	Health professional	Health professional applying DSM-5 criteria	NR	NR (NR)	NR (NR)
Dong 2017a[121]	China	Observational	Male university students with IGD, RGU, or NLFGU; normal/corrected to normal vision	58	NR (NR)	NR	DSM-5 criteria for IGD	NR	NR (NR)	7.5 (0.71)
Dong 2017b[122]	China	Cross-sectional	Male university students	108	20.2 NR (1.7 NR)	NR	DSM-5 criteria for IGD	NR	NR (NR)	NR (NR)
Dong 2018b[123]	China	Observational	University student gamers with IGD or RGU	119	21.14 (2.43)	NR	DSM-5 criteria for IGD	NR	NR (NR)	5.8 (1.68)

Study	Country	Study Design	Study Population Description	*N*	Age (years)<br>*M (SD) or M (range)*	IGD Reporting Method	IGD Measure/ Instrument/ Assessment	IGD Num/Den	IGD Prevalence (%)<br>*Prevalence (95% CI)*	IGD Score<br>*M (SD)*
Han 2015[124]	South Korea	Cross-sectional	Male gamers in their thirties with IGD, or males in their thirties with AD	31	NR (NR)	NR	DSM-5 criteria for IGD	NR	NR (NR)	NR (NR)
Jeong 2017[125]	South Korea	Case-control	Male adults with IGD	60	NR (NR)	Health professional	Health professional applying DSM-5 criteria	NR	NR (NR)	NR (NR)
Kim 2015[126]	South Korea	Cross-sectional	Male gamers with IGD	45	NR (NR)	NR	DSM-5 criteria for IGD	NR	NR (NR)	NR (NR)
Kim 2016a[127]	South Korea	Case-control	Males with IGD	60	NR (NR)	Health professional	Health professional applying DSM-5 criteria	NR	NR (NR)	NR (NR)
Lee 2017a[128]	South Korea	Cross-sectional	Male gamers 18-28y with IGD	61	23.5 (2.7 NR)	Health professional	Health professional applying DSM-5 criteria	NR	NR (NR)	NR (NR)
Lee 2018b[129]	South Korea	Cross-sectional	Males 19-29y with IGD, or IGD and ADHD	60	23.7 (2.5)	Health professional	Health professional applying DSM-5 criteria	NR	NR (NR)	NR (NR)

Study	Country	Study Design	Study Population Description	*N*	Age (years)<br><br>*M (SD) or M (range)*	IGD Reporting Method	IGD Measure/ Instrument/ Assessment	IGD Num/Den	IGD Prevalence (%)<br><br>*Prevalence (95% CI)*	IGD Score<br><br>*M (SD)*
Lee 2015[130]	South Korea	Observational	Male adolescent gamers with IGD	36	NR (NR)	Health professional	Health professional applying DSM-5 criteria	NR	NR (NR)	NR (NR)
Paik 2017b[100]	South Korea	Case-control	Adults 19-47y with IGD	150	NR (NR)	NR	DSM-5 criteria for IGD	39/115	33.91* (NR)	NR (NR)
Park 2017a[131]	South Korea	Cross-sectional	Children and adolescent males with ADHD, or with ADHD and IGD	46	NR (NR)	Health professional	Health professional applying DSM-5 criteria	NR	NR (NR)	NR (NR)
Park 2017b[132]	South Korea	Observational	Male gamers with IGD or AUD	77	NR (NR)	Health professional	Health professional applying DSM-5 criteria	NR	NR (NR)	NR (NR)
Park 2018[133]	South Korea	Controlled before-after	Male gamers 18-38y with IGD	62	NR (18 to 38)	Health professional	Health professional applying DSM-5 criteria	NR	NR (NR)	NR (NR)
Park 2017d[134]	South Korea	Cross-sectional	Adult males 18-60y who were gamers with IGD, or had AUD	92	NR (18 to 60)	Health professional	Health professional applying DSM-5 criteria	NR	NR (NR)	NR (NR)

Study	Country	Study Design	Study Population Description	*N*	Age (years)<br><br>*M (SD) or M (range)*	IGD Reporting Method	IGD Measure/ Instrument/ Assessment	IGD Num/Den	IGD Prevalence (%)<br><br>*Prevalence (95% CI)*	IGD Score<br><br>*M (SD)*
Sakuma 2017[135]	Japan	Uncontrolled before-after	Males with IGD	10	16.2 (2.15)	Health professional	Health professional applying DSM-5 criteria	NR	NR (NR)	NR (NR)
Seok 2018[136]	South Korea	Cross-sectional	Males 20-26y with IGD	40	NR (NR)	Health professional	Health professional applying DSM-5 criteria	NR	NR (NR)	NR (NR)
Shin 2018[137]	South Korea	Controlled after	Male adolescents and young adults 12-25y with IGD	64	NR (NR)	Health professional	Health professional applying DSM-5 criteria	NR	NR (NR)	NR (NR)
Son 2015[138]	South Korea	Cross-sectional	Male gamers with IGD, or males with AUD	76	NR (NR)	Health professional	Health professional applying DSM-5 criteria	34/76	44.74* (NR)	NR (NR)
Wang 2016a[139]	China	Observational	Males	72	NR (NR)	Health professional	Health professional applying DSM-5 criteria	NR	NR (NR)	NR (NR)
Wang 2016b[140]	China	Observational	Males	40	NR (NR)	NR	DSM-5 criteria for IGD	NR	NR (NR)	NR (NR)

Study	Country	Study Design	Study Population Description	*N*	Age (years)<br><br>*M (SD) or M (range)*	IGD Reporting Method	IGD Measure/ Instrument/ Assessment	IGD Num/Den	IGD Prevalence (%)<br><br>*Prevalence (95% CI)*	IGD Score<br><br>*M (SD)*
Wang 2017c[141]	China	Observational	Male university students	37	NR (NR)	NR	DSM-5 criteria for IGD	NR	NR (NR)	6.61 (0.92)
Wang 2018b[142]	China	Cross-sectional	Males	80	NR (NR)	Self-report	DSM-5 criteria for IGD	NR	NR (NR)	5.96 (1.01)
Wang 2017d[143]	China	Observational	Males	39	NR (NR)	NR	DSM-5 criteria for IGD	NR	NR (NR)	5.2 (0.8)
Wang 2017f[144]	China	Observational	Male students	63	NR (NR)	NR	DSM-5 criteria for IGD	NR	NR (NR)	NR (NR)
Wang 2017e[145]	China	Observational	Male university students	39	NR (NR)	NR	DSM-5 criteria for IGD	NR	NR (NR)	NR (NR)
Wu 2018b[146]	China	Observational	Male gamers with IGD	44	NR (NR)	Health professional	Health professional applying DSM-5 criteria	NR	NR (NR)	5.73 (0.94)
Yip 2018[147]	China	Observational	Male adult gamers 18-26y with IGD	47	NR (18 to 26)	Health professional	Health professional applying DSM-5 criteria	NR	NR (NR)	NR (NR)

Study	Country	Study Design	Study Population Description	*N*	Age (years)<br><br>*M (SD) or M (range)*	IGD Reporting Method	IGD Measure/ Instrument/ Assessment	IGD Num/Den	IGD Prevalence (%)<br><br>*Prevalence (95% CI)*	IGD Score<br><br>*M (SD)*
Youh 2017[148]	South Korea	Cross-sectional	Males 13-30y with MDD and IGD or with MDD	29	NR (13 to 30)	π	DSM-5 criteria for IGD	NR	NR (NR)	NR (NR)
Zhang 2016[149]	China	Observational	Male student gamers	40	NR (NR)	NR	DSM-5 criteria for IGD	NR	NR (NR)	NR (NR)
Females – Clinical Population										
Dong 2018b[123]	China	Observational	University student gamers with IGD or RGU	119	21.17 (2.10)	NR	DSM-5 criteria for IGD	NR	NR (NR)	5.72 (1.07)
Paik 2017b[100]	South Korea	Case-control	Adults 19-47y with IGD	150	NR (NR)	NR	DSM-5 criteria for IGD	24/35	69.00* (NR)	NR (NR)

Note: IGD prevalence values were charted to two decimal places. All other values are as reported by authors.
***** Calculated using raw data provided in the study; **‡** Median; **^** Inter-Quartile Range; **π** Unclear

CI: *Confidence Interval*; **C-VAT**: *Clinical Video game Addiction Test;* **GAIT***: The Gaming Addiction Identification Test;* **IGD**: *Internet Gaming Disorder*; **IGDI**: *Internet Gaming Disorder Interview;* **IGDQ**: *Internet Gaming Disorder Questionnaire***; IGDS9-SF**: *Internet Gaming Disorder Scale - Short Form*; **IGDT-10**: *Ten-Item Internet Gaming Disorder Test*; **M**: *Mean*; **N**: *Sample size*; **NR**: *Data not reported in the study*; **Num/Den**: *Prevalence Numerator/Prevalence Denominator*; **PVP Scale**: *Problematic Videogame Playing Scale*; **SD**: *Standard Deviation;* **USA**: *United States of America;* **VAT***: Video game Addiction Test*

Ranges for measures used: **DSM-5 criteria for IGD**: *0-9*; **IGD Checklist - 9 item**: *No lower or upper limit reported*; **IGD Scale - 9 item dichotomous**: *0-9*; **IGD Scale - dichotomous**: *No lower or upper limit reported*; **IGD Scale - polytomous**: *No lower or upper limit reported*; **IGDQ - German**: *0-9**; **IGDS9-SF**: *9-45*; **IGDS9-SF - Italian**: *9-45**; **IGDT-10**: *0-9*; **PVP Scale**: *0-9*; **VAT**: *0-56**

Appendix G. Study and population characteristics for studies with general or clinical populations, by age groups

Study	Country	Study Design	Study Population Description	*N*	Age (years)<br><br>*M (SD) or M (range)*	Gender / Sex (%)<br><br>*(Male/ Female/ Other)*	IGD Reporting Method	IGD Measure/ Instrument/ Assessment	IGD Num/Den	IGD Prevalence (%)<br><br>*Prevalence (95% CI)*	IGD Score<br><br>*M (SD)*
Children 0-19 years old† – General Population											
Wichstrøm 2018[29]	Norway	Observational	Children 10y	740	8, 10 NR (NR)	51/49/0	Health professional	IGDI	14/740	1.70 (0.70 to 2.70)	NR (NR)
Milani 2018[36]	Italy	Cross-sectional	Students 9-19y	612	13.94 (2.44)	53/47/0	Self-report	VGA questionnaire (revised)	13/612	2.10 (NR)	NR (NR)
Jeong 2018[53]	South Korea	Cross-sectional	Adolescents from the 3rd, 4th, and 7th grades	273	NR (NR)	45/55/0*	Health professional	Health professional applying DSM-5 criteria	45/273	16.48* (NR)	NR (NR)
Jeong 2018[53]	South Korea	Cross-sectional	Adolescents from the 3rd, 4th, and 7th grades	273	NR (NR)	45/55/0*	Self-report	DSM-5 criteria for IGD	47/273	17.22* (NR)	NR (NR)
Jeong 2018[53]	South Korea	Cross-sectional	Adolescents from the 3rd, 4th, and 7th grades	273	NR (NR)	45/55/0*	Health professional	Health professional applying DSM-5 criteria	43/834	5.16* (NR)	NR (NR)

Study	Country	Study Design	Study Population Description	*N*	Age (years)<br>*M (SD) or M (range)*	Gender / Sex (%)<br>*(Male/ Female/ Other)*	IGD Reporting Method	IGD Measure/ Instrument/ Assessment	IGD Num/Den	IGD Prevalence (%)<br>*Prevalence (95% CI)*	IGD Score<br>*M (SD)*
Adolescents 10-19 years old – General Population											
Hawi 2018[1]	Lebanon	Cross-sectional	High school students 15-19y	524	16.2 (1.0)	NR/48/NR	Self-report	IGD-20 Test	NR	9.20 (NR)	NR (NR)
Wu 2017b[2]	Iran	Cross-sectional	Adolescent students 12-19y	2363	15.6 (1.2)	NR/65/NR	Self-report	IGDS9-SF	NR	NR (NR)	24.0 (7.1)
Buiza-Aguado 2018[5]	Spain	Cross-sectional	Students 12-18yfrom Málaga	708	15.6 (2.7)	44/56/0	Self-report	IGD Scale - 9 item dichotomous	59/708	8.30 (NR)	2.1 (1.8)
Lemmens 2015[12]	Netherlands	Cross-sectional	Adults and adolescents 13-40y	2444	17.6 (2.2)	51/NR/NR	Self-report	IGD Scale - 9 item dichotomous	NR	3.90 (NR)	NR (NR)
								IGD Scale - dichotomous	NR	NR (NR)	3.22 (5.07)
								IGD Scale - polytomous	NR	NR (NR)	1.51 (0.91)
Pontes 2017a[16]	Portugal	Cross-sectional	Students 10-18y in grades 6, 7, 8, and 9	509	13.02 (1.64)	NR/54/NR	Self-report	IGDS9-SF	NR	NR (NR)	15.92 (6.99)

Study	Country	Study Design	Study Population Description	*N*	Age (years)<br><br>*M (SD) or M (range)*	Gender / Sex (%)<br><br>*(Male/ Female/ Other)*	IGD Reporting Method	IGD Measure/ Instrument/ Assessment	IGD Num/Den	IGD Prevalence (%)<br><br>*Prevalence (95% CI)*	IGD Score<br><br>*M (SD)*
Pontes 2016[17]	Slovenia	Cross-sectional	Students in grade 8 12-16y	1071	13.44 (0.59)	NR/50/NR	Self-report	IGDS9-SF	26/1071	2.60 (1.70 to 3.70)	NR (NR)
Rehbein 2015[21]	Germany	Cross-sectional	Students 13-18y in grade 9	11003	14.88 (0.74)	49/51/0*	Self-report	Video Game Dependency Scale	128/11003	1.16 (0.96 to 1.36)	NR (NR)
Vadlin 2018[24]	Sweden	Observational	Adolescents	1576	13, 15, 16, 18 NR (NR)	58/NR/NR	Self-report	GAIT	NR	NR (NR)	NR (NR)
Wartberg 2017a[25]	Germany	Cross-sectional	Adolescents 12-14y	1095	12.99 (0.82)	49/51/0	Self-report	IGD Scale - 9 item dichotomous	260/1095	23.74* (NR)	NR (NR)
Wartberg 2017b[26]	Germany	Cross-sectional	Adolescents 12-14y	1095	12.99 (0.82)	49/51/0	Self-report	IGD Scale - 9 item dichotomous	260/1095	5.50 (NR)	NR (NR)
Wartberg 2018[28]	Germany	Observational	Adolescents 12-14y	1095	*Time 1:* 12.99 (0.82)	49/51/0	NR	IGD Scale - 9 item dichotomous	242/985	24.57* (NR)	NR (NR)
					Time 2: 13.89 (0.89)	49/51/0	NR	IGD Scale - 9 item dichotomous	257/985	26.09* (NR)	NR (NR)
Gunuc 2015[33]	Turkey	Cross-sectional	Adolescents 15-18y	131	NR (15 to 18)	NR/NR/NR	Self-report	VGAQ	21/131	16.00 (NR)	21.435 (6.437)

Study	Country	Study Design	Study Population Description	*N*	Age (years)<br>*M (SD) or M (range)*	Gender / Sex (%)<br>*(Male/ Female/ Other)*	IGD Reporting Method	IGD Measure/ Instrument/ Assessment	IGD Num/Den	IGD Prevalence (%)<br>*Prevalence (95% CI)*	IGD Score<br>*M (SD)*
Lopez-Fernandez 2014[35]	Spain	Cross-sectional	Adolescent students 11-18y	1996	NR (11 to 18)	41/58/NR*	Self-report	PVP Scale	81/1047	7.74* (NR)	1.74 (1.70)
Lopez-Fernandez 2014[35]	Great Britain	Cross-sectional	Adolescent students 11-18y	1996	NR (11 to 18)	41/58/NR*	Self-report	PVP Scale	179/949	18.86* (NR)	2.47 (2.33)
Peeters 2018[38]	Netherlands	Observational	Adolescents 11-15y	544	13.9 (0.74)	NR/49/NR	Self-report	IGD Scale - 9 item dichotomous	NR	NR (NR)	*Time 1:* 0.686 (1.348)<br>*Time 2:* 0.738 (1.453)
Tejeiro 2016[40]	Spain	Cross-sectional	Adolescent students 11-17y and adult students 20-50y	909	NR (NR)	50/50/0	Self-report	PVP Scale	NR	NR (NR)	1.67 (1.55)
Stubblefield 2017[46]	USA	Cross-sectional	Children 11-17y	454	13.7 (1.9)	53/47/NR	Self-report	Problem gaming instrument	37/454	8.20 (NR)	NR (NR)

Study	Country	Study Design	Study Population Description	*N*	Age (years) *M (SD) or M (range)*	Gender / Sex (%) *(Male/ Female/ Other)*	IGD Reporting Method	IGD Measure/ Instrument/ Assessment	IGD Num/Den	IGD Prevalence (%) *Prevalence (95% CI)*	IGD Score *M (SD)*
Jo 2018[54]	South Korea	Cross-sectional	Adolescent gamers 10-19y	121	14‡(10 to 19)	26/74/0	Health professional	Health professional applying DSM-5 criteria	46/121	38.00 (NR)	NR (NR)
King 2016[56]	Australia	Cross-sectional	Secondary school students 12y and older	824	14.1 (1.5)	51/49/0*	Self-report	IGD Checklist - 12 item	26/824	3.10 (NR)	NR (NR)
Lee 2017b[58]	South Korea	Cross-sectional	First year middle school students	330	NR (NR)	51/49/0	Health professional	Health professional applying DSM-5 criteria	16/330	4.90 (NR)	NR (NR)
									Author-defined severe cut-off: 4/330	1.21* (NR)	NR (NR)
Rao 2017[64]	China	Cross-sectional	Students in grades 7, 8, 9, and 10	2590	14.1 (NR)	40/60/NR*	Self-report	IGDS9-SF	NR	NR (NR)	NR (NR)
Yu 2016[71]	South Korea	Cross-sectional	Middle school students 13-15y	2014	14.5 (0.50)	50/51/NR*	Self-report	DSM-5 criteria for IGD	119/2014	5.90 (NR)	16.63 (7.6)
King 2017c[76]	Australia	Cross-sectional	Students 12-17y	824	14.1 (1.5)	51/49/NR*	Self-report	IGD Checklist - 12 item	25/799	3.10 (NR)	NR (NR)

Study	Country	Study Design	Study Population Description	*N*	Age (years)<br>*M (SD) or M (range)*	Gender / Sex (%)<br>*(Male/ Female/ Other)*	IGD Reporting Method	IGD Measure/ Instrument/ Assessment	IGD Num/Den	IGD Prevalence (%)<br>*Prevalence (95% CI)*	IGD Score<br>*M (SD)*
Schneider 2018[78]	Australia	Cross-sectional	Adolescents 12-19y	823	14.2 (1.4)	51/49/0*	Self-report	IGD Checklist - 12 item	26/823	3.20 (NR)	NR (NR)
Adults 18 years and older – General Population											
Deleuze 2017[6]	Belgium	Observational	Adult gamers 18-39y	97	22.21 (3.73)	NR/87/NR	Self-report	DSM-5 criteria for IGD	32/97	33.00 (NR)	NR (NR)
Jeromin 2016a[8]	Germany	Observational	Adults	87	NR (NR)	0/100/0	Self-report	IGDQ - German	29/686	5.30 (NR)	NR (NR)
Jeromin 2016b[9]	Germany	Cross-sectional	Adult gamers 18-75y	894	26.49 (8.46 NR)	13/87/0	Self-report	IGDQ - German	71/894	7.94 (1.86π)	1.70 (1.86π)
Lemmens 2015[12]	Netherlands	Cross-sectional	Adults and adolescents 13-40y	2444	*Ages 31-40*: 35.9 (2.8)	51/NR/NR	Self-report	IGD Scale - 9 item dichotomous	NR	5.50 (NR)	NR (NR)
					Ages 21-30: 25.1 (2.8)	51/NR/NR	Self-report	IGD Scale - 9 item dichotomous	NR	6.70 (NR)	NR (NR)
					Ages 31-40: 35.9 (2.8)	51/NR/NR	Self-report	IGD Scale - dichotomous	NR	NR (NR)	4.48 (5.14)
					Ages 21-30: 25.1 (2.8)	51/NR/NR	Self-report	IGD Scale - dichotomous	NR	NR (NR)	4.63 (5.90)

Study	Country	Study Design	Study Population Description	*N*	Age (years)<br><br>*M (SD) or M (range)*	Gender / Sex (%)<br><br>*(Male/ Female/ Other)*	IGD Reporting Method	IGD Measure/ Instrument/ Assessment	IGD Num/Den	IGD Prevalence (%)<br><br>*Prevalence (95% CI)*	IGD Score<br><br>*M (SD)*
					Ages 31-40: 35.9 (2.8)	51/NR/NR	Self-report	IGD Scale - polytomous	NR	NR (NR)	1.57 (0.87)
					Ages 21-30: 25.1 (2.8)	51/NR/NR	Self-report	IGD Scale - polytomous	NR	NR (NR)	1.61 (0.97)
Pontes 2017b[18]	United Kingdom	Cross-sectional	Adult gamers	272	41.61 (14.03)	NR/51/NR	Self-report	IGDS9-SF	NR	NR (NR)	14.04 (5.67)
De Pasquale 2018[30]	Italy	Cross-sectional	Students 18-25y	221	21.56 (1.42)	58/42/0*	Interview, not specified	IGDS9-SF	33/221	14.90 (NR)	NR (NR)
Laconi 2017[34]	France	Cross-sectional	Adult gamers 18-30y	418	21.9 (3)	49/51/0	Self-report	IGDT-10	8/418	1.90 (NR)	28.3 (5.3)
Taquet 2017[39]	France	Cross-sectional	Adult gamers 18-53y	124	25.274 (7.424)	15/85/0*	Self-report	PVP Scale	NR	NR (NR)	4.226 (1.878)
Tejeiro 2016[40]	Spain	Cross-sectional	Adolescent students 11-17y and adult students 20-50y	909	NR (NR)	50/50/0	Self-report	PVP Scale	NR	NR (NR)	0.96 (1.31)

Study	Country	Study Design	Study Population Description	*N*	Age (years)<br><br>*M (SD) or M (range)*	Gender / Sex (%)<br><br>*(Male/ Female/ Other)*	IGD Reporting Method	IGD Measure/ Instrument/ Assessment	IGD Num/Den	IGD Prevalence (%)<br><br>*Prevalence (95% CI)*	IGD Score<br><br>*M (SD)*
Przybylski 2016a[19]	United Kingdom	Cross-sectional	Adults 18y and older	1899	NR (NR)	50/50/0*	Self-report	DSM-5 criteria for IGD	NR	NR (NR)	NR (NR)
Przybylski 2017b[20]	United Kingdom	Cross-sectional	Adults	1899	NR (NR)	50/50/0*	Self-report	DSM-5 criteria for IGD	NR	0.47 (0.23 to 0.93)	NR (NR)
Przybylski 2016b[80]	Canada, Germany, United Kingdom, USA	Cross-sectional	Adults 18-24y	10009	NR (18 to 24)	50/50/0*	Self-report	DSM-5 criteria for IGD	NR	NR (NR)	NR (NR)
Przybylski 2017c[81]	Canada, Germany, United Kingdom, USA	Cross-sectional	Adults 18-24y	10099	NR (18 to 24)	49/50/NR*	Self-report	DSM-5 criteria for IGD	NR	0.68 (0.53 to 0.87)	NR (NR)
Pontes 2017b[18]	USA	Cross-sectional	Adult gamers	405	32.57 (11.33)	NR/62/NR	Self-report	IGDS9-SF	NR	NR (NR)	18.06 (7.36)
Stockdale 2018[45]	USA	Cross-sectional	Adult university student gamers	174	NR (NR)	NR/NR/NR	Self-report	IGD Scale - 9 item	87/1205	7.22* (NR)	NR (NR)

**Study**	**Country**	**Study Design**	**Study Population Description**	***N***	**Age (years)**<br>*M (SD) or M (range)*	**Gender / Sex (%)**<br>*(Male/ Female/ Other)*	**IGD Reporting Method**	**IGD Measure/ Instrument/ Assessment**	**IGD Num/Den**	**IGD Prevalence (%)**<br>*Prevalence (95% CI)*	**IGD Score**<br>*M (SD)*
Weinstein 2017[47]	USA	Observational	Adults	2316	NR (NR)	62/38/0*	Health professional	Health professional applying DSM-5 criteria	NR	*Time 1:* 1.49 (1.11 to 2.00)	0.56 (1.13)
										Time 2: 0.99 (0.65 to 1.51)	0.47 (0.97)
Allen 2018[48]	USA	Cross-sectional	Student gamers 18-40y	315	19.34 (2.01)	19/81/0	Self-report	IGD Scale - 27 item polytomous	18/315	5.70 (NR)	0.78 (0.81)
Bargeron 2017[49]	USA	Cross-sectional	Adults	257	21.81 (6.80)	NR/NR/NR	Self-report	DSM-5 criteria for IGD	21/257	8.70 (NR)	1.92 (1.77)
Carlisle 2018[50]	USA	Cross-sectional	Adult gamers 18-95y	1881	28.27 (18 to 95)	39/59/2	Self-report	IGDT-10	79/1881	4.20 (NR)	1.16 (1.54)
Sioni 2017[52]	USA	Cross-sectional	Adult gamers 18-77y	394	34.3 (11.6)	NR/50/NR	Self-report	IGD Scale - 9 item dichotomous	102/394	25.90 (NR)	11.8 (2.7)
Przybylski 2017a[42]	USA	Cross-sectional	Adults 18-24y	1247	NR (18 to 24)	42/58/0*	Self-report	DSM-5 criteria for IGD	NR	1.04 (0.58 to 1.83)	NR (NR)
Przybylski 2017d[43]	USA	Cross-sectional	Adults	5777	46.59 (17.80)	58/42/0*	Self-report	DSM-5 criteria for IGD	NR	0.32 (0.18 to 0.56)	NR (NR)

Study	Country	Study Design	Study Population Description	*N*	Age (years)<br><br>*M (SD) or M (range)*	Gender / Sex (%)<br><br>*(Male/ Female/ Other)*	IGD Reporting Method	IGD Measure/ Instrument/ Assessment	IGD Num/Den	IGD Prevalence (%)<br><br>*Prevalence (95% CI)*	IGD Score<br><br>*M (SD)*
Pontes 2017b[18]	India	Cross-sectional	Adult gamers	336	30.37 (8.90)	NR/68/NR	Self-report	IGDS9-SF	NR	NR (NR)	25.57 (7.64)
Kim 2016b[55]	South Korea	Cross-sectional	Adult gamers 20-49y	3041	NR (NR)	40.0/60/0	Self-report	DSM-5 criteria for IGD	419/3041	13.80 (NR)	NR (NR)
					NR (20 to 29)	40.0/60/0	Self-report	DSM-5 criteria for IGD	160/1221	13.10* (NR)	NR (NR)
					NR (30 to 39)	40.0/60/0	Self-report	DSM-5 criteria for IGD	175/1215	14.40* (NR)	NR (NR)
					NR (40 to 49)	40.0/60/0	Self-report	DSM-5 criteria for IGD	84/605	13.88* (NR)	NR (NR)
King 2017a[57]	Australia	Cross-sectional	Adult gamers 18-56y	630	25.8 (7.1)	NR/76/NR*	Self-report	IGD Checklist - 9 item	20/630	3.17* (NR)	NR (NR)
Na 2017a[59]	South Korea	Cross-sectional	Gamers 20-49y	2923	NR (20 to 49)	29/71/0*	Self-report	DSM-5 criteria for IGD	494/2923	16.90* (NR)	NR (NR)
					NR (20 to 29)	29/71/0*	Self-report	DSM-5 criteria for IGD	180/1147	15.69* (NR)	NR (NR)
					NR (30 to 39)	29/71/0*	Self-report	DSM-5 criteria for IGD	222/1233	18.00* (NR)	NR (NR)
					NR (40 to 49)	29/71/0*	Self-report	DSM-5 criteria for IGD	92/543	16.94* (NR)	NR (NR)

Study	Country	Study Design	Study Population Description	*N*	Age (years) *M (SD) or M (range)*	Gender / Sex (%) *(Male/ Female/ Other)*	IGD Reporting Method	IGD Measure/ Instrument/ Assessment	IGD Num/Den	IGD Prevalence (%) *Prevalence (95% CI)*	IGD Score *M (SD)*
Na 2017b[60]	South Korea	Cross-sectional	Adults 20-49y	1819	NR (20 to 49)	47/53/0	Self-report	DSM-5 criteria for IGD	257/1819	14.13* (NR)	NR (NR)
Na 2017b[60]	South Korea	Cross-sectional	Adults 20-49y	1819	NR (20 to 29)	47/53/0	Self-report	DSM-5 criteria for IGD	89/669	13.30* (NR)	NR (NR)
Na 2017b[60]	South Korea	Cross-sectional	Adults 20-49y	1819	NR (30 to 39)	47/53/0	Self-report	DSM-5 criteria for IGD	120/804	14.93* (NR)	NR (NR)
Na 2017b[60]	South Korea	Cross-sectional	Adults 20-49y	1819	NR (40 to 39)	47/53/0	Self-report	DSM-5 criteria for IGD	48/346	13.87* (NR)	NR (NR)
Paik 2017a[61]	South Korea	Cross-sectional	Adult gamers 20-39y	3058	26.95 (5.859)	NR/NR/NR	Self-report	DSM-5 criteria for IGD	396/3058	12.90 (NR)	NR (NR)
Rho 2017[65]	South Korea	Cross-sectional	Adult gamers 20-49y	3568	NR (20 to 49)	43/57/0	Self-report	DSM-5 criteria for IGD	481/3568	13.50 (NR)	NR (NR)
Rho 2017[65]	South Korea	Cross-sectional	Adult gamers 20-49y	3568	NR(20 to 29)	43/57/0	Self-report	DSM-5 criteria for IGD	170/1259	13.50* (NR)	NR (NR)
Rho 2017[65]	South Korea	Cross-sectional	Adult gamers 20-49y	3568	NR(30 to 39)	43/57/0	Self-report	DSM-5 criteria for IGD	215/1559	13.79* (NR)	NR (NR)
Rho 2017[65]	South Korea	Cross-sectional	Adult gamers 20-49y	3568	NR(40 to 49)	43/57/0	Self-report	DSM-5 criteria for IGD	96/750	12.80* (NR)	NR (NR)

Study	Country	Study Design	Study Population Description	*N*	Age (years) *M (SD) or M (range)*	Gender / Sex (%) *(Male/ Female/ Other)*	IGD Reporting Method	IGD Measure/ Instrument/ Assessment	IGD Num/Den	IGD Prevalence (%) *Prevalence (95% CI)*	IGD Score *M (SD)*
Sigerson 2017[66]	China	Cross-sectional	Adult gamers 18-60y	502	37.1 (13.3)	50/50/0*	Self-report	C-IGDS	NR	NR (NR)	1.29 (1.73)
Wu 2017a[69]	China, Taiwan	Cross-sectional	Adult online gamers 18-82 y	383	23.7 (6.7)	45/55/0	Self-report	DSM-5 criteria for IGD	64/383	16.71* (NR)	NR (NR)
Wu 2018a[70]	China	Cross-sectional	Adults 18-97y	1000	40.0 (15.3)	56/44/0	Self-report	DSM-5 criteria for IGD	20/1000	2.00 (1.10 to 2.90)	NR (NR)
Yu 2018[72]	China	Cross-sectional	Adult gamers 18-67y	327	31.93 (9.04)	NR/79/NR	Self-report	DSM-5 criteria for IGD	NR	2.30 (NR)	NR (NR)
Adams 2018[73]	Australia	Observational	Adult gamers 18-29y	125	23.34 (3.39)	25/75/0	Self-report	IGDS9-SF	NR	NR (NR)	*Time 1:* 19.48 (7.07)
											Time 2: 18.67 (SD 6.86)
											Time 3: 17.78 (SD 5.80)

Study	Country	Study Design	Study Population Description	*N*	Age (years) *M (SD) or M (range)*	Gender / Sex (%) *(Male/ Female/ Other)*	IGD Reporting Method	IGD Measure/ Instrument/ Assessment	IGD Num/Den	IGD Prevalence (%) *Prevalence (95% CI)*	IGD Score *M (SD)*
Burleigh 2018[74]	Australia	Observational	Adult gamers 18-29y	125	23.02 (3.43)	25/75/0	Self-report	IGDS9-SF	NR	NR (NR)	*Time 1:* 19.48 (7.06)<br>*Time 2:* 18.67 (6.86)<br>*Time 3:* 17.78 (5.80)
Liew 2018[77]	Australia	Observational	Adult gamers 18-29y	125	NR (18 to 29)	25/75/0	Self-report	IGDS9-SF	NR	NR (NR)	*Time 1:* 19.44 (7.07)<br>*Time 2:* 18.8 (6.91)<br>*Time 3:* 17.95 (5.93)
Children 0-19 years old† – Clinical Population											
Krossbakken 2018[83]	Norway	RCT	Guardians of children 8-12y	1657	10.1 (NR)	46/53/NR*	Parent report	DSM-5 criteria for IGD	NR	NR (NR)	NR (NR)

Study	Country	Study Design	Study Population Description	*N*	Age (years)<br><br>*M (SD) or M (range)*	Gender / Sex (%)<br><br>*(Male/ Female/ Other)*	IGD Reporting Method	IGD Measure/ Instrument/ Assessment	IGD Num/Den	IGD Prevalence (%)<br><br>*Prevalence (95% CI)*	IGD Score<br><br>*M (SD)*
Li 2018a[99]	China	Observational	Primary and secondary school students 8-15y	241	12.09 (1.41)	43/NR/NR	Self-report	K-scale - Korean Internet Addiction Scale for Adolescents	NR	NR (NR)	*Time 1:* 31.33 (6.38)<br><br>*Time 2:* 30.92 (6.87)
Adolescents 10-19 years old – Clinical Population											
Koo 2017[96]	South Korea	Cross-sectional	Adolescents (middle school students)	236	13.61 (0.87)	29/71/0	Health professional	SCI-IGD	27/236	11.44* (NR)	NR (NR)
Lee 2018c[98]	South Korea	Case-control	Adolescents 12-18y with IGD	87	NR (12 to 18)	NR/NR/NR	Self-report	DSM-5 criteria for IGD	251/3166	7.93* (NR)	NR (NR)
Tian 2018[113]	China	Observational	Adolescents with IGD	88	NR (NR)	45/49/NR*	Health professional	Health professional applying DSM-5 criteria	NR	NR (NR)	NR (NR)
Adults 18 years and older – Clinical Population											
Mallorquí-Bagué 2017[84]	Spain	Cross-sectional	Adults with IGD or online gambling disorder	288	NR (NR)	5/95/0*	Health professional	Health professional applying DSM-5 criteria	27/288	9.38* (NR)	NR (NR)

Study	Country	Study Design	Study Population Description	*N*	Age (years)<br><br>*M (SD) or M (range)*	Gender / Sex (%)<br><br>*(Male/ Female/ Other)*	IGD Reporting Method	IGD Measure/ Instrument/ Assessment	IGD Num/Den	IGD Prevalence (%)<br><br>*Prevalence (95% CI)*	IGD Score<br><br>*M (SD)*
Sanders 2017[85]	Canada	Cross-sectional	Adults	542	π	39/61/7	Self-report	DSM-5 criteria for IGD	39/1238	3.20 (NR)	NR (NR)
Cai 2016[86]	China	Observational	Adult gamers with IGD	57	NR (NR)	NR/79/NR*	Self-report	DSM-5 criteria for IGD	NR	NR (NR)	NR (NR)
Kaptsis 2016[89]	Australia	Uncontrolled before-after	Adult gamers 18-25y	24	24.6 (5.1)	17/83/0*	Self-report	IGD Checklist - 9 item	NR	NR (NR)	5.89π (0.86)
King 2018a[92]	Australia	Quasi-experimental	Adult gamers 18-48y with gaming problems	186	23.4 (5.2)	NR/95/NR	Self-report	IGD Checklist - 9 item	NR	NR (NR)	NR (NR)
King 2017b[93]	Australia	Uncontrolled before-after	Adult gamers 18-36y	24	24.6 (5.1)	17/83/0*	Self-report	IGD Checklist - 9 item	9/24	37.50* (NR)	NR (NR)
Ko 2017[94]	Taiwan	Observational	Gamers 20-30y with IGD	174	NR (20 to 30)	NR/NR/NR	Health professional	Health professional applying DSM-5 criteria	NR	NR (NR)	NR (NR)
Ko 2014[95]	Taiwan	Cross-sectional	Gamers 20-30y with IGD or in IGD remission	225	NR (NR)	16/84/0	Health professional	Health professional applying DSM-5 criteria	NR	NR (NR)	NR (NR)

Study	Country	Study Design	Study Population Description	*N*	Age (years)<br><br>*M (SD) or M (range)*	Gender / Sex (%)<br><br>*(Male/ Female/ Other)*	IGD Reporting Method	IGD Measure/ Instrument/ Assessment	IGD Num/Den	IGD Prevalence (%)<br><br>*Prevalence (95% CI)*	IGD Score<br><br>*M (SD)*
Lee 2016[97]	South Korea	Observational	Adult gamers with IGD	48	NR (NR)	NR/NR/NR	Health professional	Health professional applying DSM-5 criteria	NR	NR (NR)	NR (NR)
Paik 2017b[100]	South Korea	Case-control	Adults 19-47y with IGD	150	30.09 (6.343)	NR/77/NR	NR	DSM-5 criteria for IGD	63/150	42.00* (NR)	NR (NR)
Rho 2016[102]	South Korea	Cross-sectional	Adult gamers 20-49y	1022	NR (20 to 49)	40/60/0	Self-report	DSM-5 criteria for IGD	511/3881	13.17* (NR)	NR (NR)
Ryu 2018[103]	South Korea	Cross-sectional	Young adults with IGD	123	24.92 (4.71)	NR/79/NR	Health professional	Health professional applying DSM-5 criteria	NR	NR (NR)	NR (NR)
Wang 2017a[104]	Taiwan	Cross-sectional	Adult gamers 20-30y with IGD	383	NR (20 to 30)	45/55/0	Health professional	Health professional applying DSM-5 criteria	NR	NR (NR)	NR (NR)
Yeh 2017[107]	Taiwan	Cross-sectional	Gamers 20-30y with IGD	174	NR (20 to 30)	20/80/0*	Health professional	Health professional applying DSM-5 criteria	NR	NR (NR)	NR (NR)

Study	Country	Study Design	Study Population Description	*N*	Age (years)<br><br>*M (SD) or M (range)*	Gender / Sex (%)<br><br>*(Male/ Female/ Other)*	IGD Reporting Method	IGD Measure/ Instrument/ Assessment	IGD Num/Den	IGD Prevalence (%)<br><br>*Prevalence (95% CI)*	IGD Score<br><br>*M (SD)*
Yen 2017a[108]	Taiwan	Cross-sectional	Gamers 20-30y with IGD	174	NR (20 to 30)	20/80/0*	Health professional	Health professional applying DSM-5 criteria	NR	NR (NR)	NR (NR)
Yen 2017b[109]	Taiwan	Cross-sectional	Gamers 20-30y with IGD	174	NR (20 to 30)	20/80/0*	Health professional	Health professional applying DSM-5 criteria	NR	NR (NR)	NR (NR)
King 2018b[112]	Australia	Cross-sectional	Adult gamers 18-56y	630	NR (NR)	NR/76/NR*	Self-report	IGD Scale - 9 item	20/630	4.00 (NR)	NR (NR)

Note: IGD prevalence values were charted to two decimal places. All other values are as reported by authors.
***** Calculated using raw data provided in the study; **†** Does not include adolescent-only populations; **‡** Median; **π** Unclear

CI: *Confidence Interval*; **C-IGDS**: *Chinese Internet Gaming Disorder Scale;* **GAIT**: *The Gaming Addiction Identification Test;* **IGD**: *Internet Gaming Disorder*; **IGDI**: *Internet Gaming Disorder Interview;* **IGDQ**: *Internet Gaming Disorder Questionnaire;* **IGDS9-SF**: *Internet Gaming Disorder Scale - Short Form*; **IGDT-10**: *Ten-Item Internet Gaming Disorder Test*; **M**: *Mean*; **N**: *Sample size*; **NR**: *Data not reported in the study*; **Num/Den**: *Prevalence Numerator/Prevalence Denominator*; **PVP Scale**: *Problematic Videogame Playing Scale;* **SCI-IGD**: *Structured Clinical Interview for Internet Gaming Disorder*; **SD**: *Standard Deviation;* **USA**: *United States of America;* **VGA**: *Video Game Addiction;* **VGAQ**: *Video Game Addiction Questionnaire*

Ranges for measures used: **C-IGDS**: *0-9**; **DSM-5 criteria for IGD**: *0-9*; **IGD Checklist - 9 item**: *No lower or upper limit reported*; **IGD Scale - 9 item dichotomous**: *0-9*; **IGD Scale - 27 item polytomous**: *0-135**; **IGD Scale - dichotomous**: *No lower or upper limit reported*; **IGD Scale - polytomous**: *No lower or upper limit reported*; **IGDQ - German**: *0-9**; **IGDS9-SF**: *9-45*; **IGDT-10**: *0-9*; **K-scale - Korean Internet Addiction Scale for Adolescents**: *20-80*; **PVP Scale**: *0-9*; **VGAQ**: *9-45**

Appendix H. Study and population characteristics for studies with populations undergoing an intervention (severe)

Study	Country	Study Design	Study Population Description	*N*	Age (years)<br><br>*M (SD) or M (range)*	Gender/ Sex (%)<br><br>*(Male/ Female/ Other)*	IGD Reporting Method	IGD Measure/ Instrument/ Assessment	IGD Num/Den	IGD Prevalence (%)<br><br>*Prevalence (95% CI)*	IGD Score<br><br>*M (SD)*
European Region											
González-Bueso 2018[150]	Spain	Controlled before-after	Males 12-21y with IGD	60	NR (NR)	0/100/0	Self-report and health professional	DQVMIA	NR	NR (NR)	NR (NR)
Martín-Fernández 2017[151]	Spain	Uncontrolled before-after	Children and adolescents 12-17y with IGD	59	14.83 (1.45)	NR/97/NR	Health professional	Health professional applying DSM-5 criteria	59/86	68.60* (NR)	NR (NR)
Region of the Americas											
Li 2018b[152]	USA	RCT	Adult gamers 18-35y who met 3 or more DSM-5 IGD criteria	30	25 (5.4)	17/80/3	Health professional	Health professional applying DSM-5 criteria	23/30	76.60 (NR)	6.0 (1.9)
Li 2017b[153]	USA	RCT	Adults gamers 18-35y who met 3 or more DSM-5 IGD criteria	30	25.0 (5.4)	17/80/3	Health professional	Health professional applying DSM-5 criteria	23/30	76.60 (NR)	6.0 (1.9)

Study	Country	Study Design	Study Population Description	*N*	Age (years)<br><br>*M (SD) or M (range)*	Gender/ Sex (%)<br><br>*(Male/ Female/ Other)*	IGD Reporting Method	IGD Measure/ Instrument/ Assessment	IGD Num/Den	IGD Prevalence (%)<br><br>*Prevalence (95% CI)*	IGD Score<br><br>*M (SD)*
Li 2017a[51]	USA	RCT	Adults	30	25.0 (5.4)	17/80/3.3	Self-report	DSM-5 criteria for IGD	23/30	76.60 (NR)	5.7 (1.9)
Western Pacific Region											
Bae 2018[154]	South Korea	Uncontrolled before-after	Male adults with IGD or ibGD	47	NR (NR)	0/100/0	NR	DSM-5 criteria for IGD	NR	NR (NR)	NR (NR)
Kim 2017b[155]	South Korea	Controlled before-after	Male gamers with IGD	49	NR (NR)	0/100/0	Health professional	Health professional applying DSM-5 criteria	NR	NR (NR)	NR (NR)
Lim 2016[156]	South Korea	Controlled before-after	Male gamers with IGD	84	NR (NR)	0/100/0	Health professional	Health professional applying DSM-5 criteria	NR	NR (NR)	NR (NR)
Park 2016a[157]	South Korea	RCT	Adolescent males 13-18y with ADHD and IGD	84	NR (13 to 18)	0/100/0	Health professional	Health professional applying DSM-5 criteria	84/106	79.25* (NR)	NR (NR)
Park 2017c[158]	South Korea	Controlled before-after	Males with IGD	47	NR (NR)	0/100/0	Health professional	Health professional applying DSM-5 criteria	NR	NR (NR)	NR (NR)

Study	Country	Study Design	Study Population Description	*N*	Age (years)<br><br>*M (SD) or M (range)*	Gender/ Sex (%)<br><br>*(Male/ Female/ Other)*	IGD Reporting Method	IGD Measure/ Instrument/ Assessment	IGD Num/Den	IGD Prevalence (%)<br><br>*Prevalence (95% CI)*	IGD Score<br><br>*M (SD)*
Song 2016[159]	South Korea	RCT	Adolescent and adult males 13-45y with problematic gaming	119	NR (13 to 45)	0/100/0	Health professional	Health professional applying DSM-5 criteria	119/236	50.42* (NR)	NR (NR)
Yao 2017[160]	China	Controlled before-after	Adult gamers 18-26y with IGD	46	NR (18 to 26)	NR/NR/NR	Health professional	Health professional applying DSM-5 criteria	NR	NR (NR)	NR (NR)

Note: No data found for African region, Eastern Mediterranean region, and South East Asia region; IGD prevalence values were charted to two decimal places. All other values are as reported by authors.
***** Calculated using raw data provided in the study

CI: *Confidence Interval*; **DQVMIA**: *Diagnostic Questionnaires for Video Games, Mobile Phone or Internet Addiction;* **IGD**: *Internet Gaming Disorder*; **M**: *Mean*; **N**: *Sample size*; **NR**: *Data not reported in the study*; **Num/Den**: *Prevalence Numerator/Prevalence Denominator*; **SD**: *Standard Deviation;* **USA**: *United States of America*

Ranges for measures used: **DSM-5 criteria for IGD**: *0-9*

Appendix I. Frequency of variables reported in populations with internet gaming disorder

Variables	Number of Times Reported in this Review*
Demographic Characteristics	
Education-related outcome	41
Intelligence	28
Job-related outcome	7
Marital status	4
Income-related outcome	3
Living situation	3
Ethnicity	2
Socioeconomic status	2
Nationality	1
Drug-related Variables	
Alcohol-related outcome	22
Smoking-related outcome	13
Medication/drug use	3
Cannabis-related outcome	2
Substance use disorder	1
Game-related Variables	
Gaming time	69
Gaming context	34
Gaming cost-related outcome	4
Gaming cognition	3
Gaming motivation	1
Game playing	1
Internet gaming behavior	1
Mental Health/Well-being	
Depression/depressiveness	67
Internet addiction	54
Anxiety	48
Impulsiveness/impulsivity	37
Attention deficit hyperactivity disorder/attention deficit disorder	24
Anger/aggression/hostility	18
Behavioral inhibition/Behavioral activation	13
Well-being/quality of life	9
Obsessive compulsive/obsession-compulsion	6
Craving	6
Self-esteem	6

Stress	6
Self-control	5
Distress	4
Gambling problem	4
Autism	3
Disruptive behavior disorder	2
Loneliness	2
Paranoid ideation	2
Personality disorder	2
Positive affect and negative affect	2
Psychiatric condition	2
Psychoticism	2
Self-perception	2
Somatization	2
Temperament and character	2
Adjustment disorder	1
Adverse childhood experience	1
Affective disorder	1
Been told they have a learning disorder	1
Burnout	1
Cognitive coping	1
Cognitive distortion	1
Cognitive functioning	1
Cognitive reappraisal and expressive suppression	1
Conduct problem	1
Confusion	1
Deviant behavior	1
Dissocial personality disorder	1
Dysthymic disorder	1
Eating disorder	1
Emotional regulation	1
Externalizing disorder	1
Family psychiatric health	1
Food addiction	1
Functional impairment	1
Gaming treatment	1
Hyperactivity/inattention	1
Hypomania	1
Internalizing disorder	1
Internet gaming withdrawal	1
Mania	1

Mental health	1
Mindfulness	1
Mood regulation disorder	1
Need satisfaction and frustration	1
Oppositional defiant disorder	1
Panic/anxiety disorder	1
Pervasive developmental disorder	1
Phobia	1
Pornography addiction	1
Problems with the social environment and education	1
Procrastination	1
Psychological resilience	1
Psychosis/schizophrenia	1
Purpose in life	1
Somatic complaint	1
Suicide	1
Tension	1
Tolerance	1
Vigor	1
Withdrawal	1
Miscellaneous	
Task-related outcome	36
Brain imaging characteristics	28
Duration of illness	4
Virtual reality experience	4
Online time	3
Clinical global impression	2
Fatigue	2
Bullying-related outcome	1
Clinical impairment	1
Complaints when Internet not available	1
Daily life disturbance	1
Decision-making	1
Disturbance of reality testing	1
Global severity index	1
Hypermasculinity	1
K symptom scale	1
School-related outcome	1
Self-efficacy	1
Sports	1

Physical/Physiological Characteristics	
Anthropometric measure	8
Sleep-related outcome	4
Biological outcome	3
Handedness	2
Exercise time	1
Knee problem	1
Physical health	1
Relationship-related Variables	
Relationship-related outcome	19
Interpersonal sensitivity	2
Community membership	1
Interpersonal problem	1
Prosocial behaviour	1
Virtual interpersonal relationship	1

***** Each variable can be reported multiple times in one study

References

1. Hawi NS, Samaha M, Griffiths MD. Internet gaming disorder in Lebanon: Relationships with age, sleep habits, and academic achievement. *J Behav Addict.* 2018;7(1):70-78.
2. Wu TY, Lin CY, Arestedt K, Griffiths MD, Brostrom A, Pakpour AH. Psychometric validation of the Persian nine-item Internet Gaming Disorder Scale - Short Form: Does gender and hours spent online gaming affect the interpretations of item descriptions? *J Behav Addict.* 2017b;6(2):256-263.
3. Arcelus J, Bouman WP, Jones BA, Richards C, Jimenez-Murcia S, Griffiths MD. Video gaming and gaming addiction in transgender people: An exploratory study. *J Behav Addict.* 2017;6(1):21-29.
4. Bouna-Pyrrou P, Muhle C, Kornhuber J, Lenz B. Internet gaming disorder, social network disorder and laterality: handedness relates to pathological use of social networks. *J Neural Transm.* 2015;122(8):1187-1196.
5. Buiza-Aguado C, Alonso-Canovas A, Conde-Mateos C, Buiza-Navarrete JJ, Gentile D. Problematic Video Gaming in a Young Spanish Population: Association with Psychosocial Health. *Cyberpsychol Behav Soc Netw.* 2018;21(6):388-394.
6. Deleuze J, Nuyens F, Rochat L, Rothen S, Maurage P, Billieux J. Established risk factors for addiction fail to discriminate between healthy gamers and gamers endorsing DSM-5 Internet gaming disorder. *J Behav Addict.* 2017;6(4):516-524.
7. Evren C, Dalbudak E, Topcu M, Kutlu N, Evren B, Pontes HM. Psychometric validation of the Turkish nine-item Internet Gaming Disorder Scale-Short Form (IGDS9-SF). *Psychiatry Res.* 2018;265:349-354.
8. Jeromin F, Rief W, Barke A. Using two web-based addiction Stroops to measure the attentional bias in adults with Internet Gaming Disorder. *J Behav Addict.* 2016a;5(4):666-673.
9. Jeromin F, Rief W, Barke A. Validation of the Internet Gaming Disorder Questionnaire in a Sample of Adult German-Speaking Internet Gamers. *Cyberpsychol Behav Soc Netw.* 2016b;19(7):453-459.
10. Király O, Sleczka P, Pontes HM, Urban R, Griffiths MD, Demetrovics Z. Validation of the Ten-Item Internet Gaming Disorder Test (IGDT-10) and evaluation of the nine DSM-5 Internet Gaming Disorder criteria. *Addict Behav.* 2017;64:253-260.
11. Lemmens JS, Hendriks SJ. Addictive Online Games: Examining the Relationship Between Game Genres and Internet Gaming Disorder. *Cyberpsychol Behav Soc Netw.* 2016;19(4):270-276.
12. Lemmens JS, Valkenburg PM, Gentile DA. The Internet Gaming Disorder Scale. *Psychol Assess.* 2015;27(2):567-582.
13. Monacis L, de Palo V, Griffiths MD, Sinatra M. Exploring Individual Differences in Online Addictions: the Role of Identity and Attachment. *Int J Ment Health Addict.* 2017;15(4):853-868.
14. Monacis L, de Palo V, Griffiths MD, Sinatra M. Validation of the Internet Gaming Disorder Scale - Short-Form (IGDS9-SF) in an Italian-speaking sample. *J Behav Addict.* 2016;5(4):683-690.
15. Monacis L, Sinatra M, Griffiths MD, de Palo V. Assessment of the Italian Version of the Internet Disorder Scale (IDS-15). *Int J Ment Health Addict.* 2018;16(3):680-691.
16. Pontes HM. Investigating the differential effects of social networking site addiction and Internet gaming disorder on psychological health. *J Behav Addict.* 2017a;6(4):601-610.
17. Pontes HM, Macur M, Griffiths MD. Internet Gaming Disorder Among Slovenian Primary Schoolchildren: Findings From a Nationally Representative Sample of Adolescents. *J Behav Addict.* 2016;5(2):304-310.

18. Pontes HM, Stavropoulos V, Griffiths MD. Measurement Invariance of the Internet Gaming Disorder Scale-Short-Form (IGDS9-SF) between the United States of America, India and the United Kingdom. *Psychiatry Res.* 2017b;257:472-478.
19. Przybylski AK. Mischievous responding in Internet Gaming Disorder research. *PeerJ.* 2016a;4:e2401.
20. Przybylski AK, Weinstein N, Murayama K. Internet Gaming Disorder: Investigating the Clinical Relevance of a New Phenomenon. *Am J Psychiatry*. 2017b;174(3):230-236.
21. Rehbein F, Kliem S, Baier D, Mosle T, Petry NM. Prevalence of Internet gaming disorder in German adolescents: diagnostic contribution of the nine DSM-5 criteria in a state-wide representative sample. *Addiction.* 2015;110(5):842-851.
22. Thomsen KR, Callesen MB, Hesse M, Kvamme TL, Pedersen MM, Pedersen MU et al. Impulsivity traits and addiction-related behaviors in youth. *J Behav Addict.* 2018;7(2):317-330.
23. Stavropoulos V, Beard C, Griffiths MD, Buleigh T, Gomez R, Pontes HM. Measurement Invariance of the Internet Gaming Disorder Scale-Short-Form (IGDS9-SF) Between Australia, the USA, and the UK. *Int J Ment Health Addict.* 2018;16(2):377-392.
24. Vadlin S, Aslund C, Nilsson KW. A longitudinal study of the individual- and group-level problematic gaming and associations with problem gambling among Swedish adolescents. *Brain and Behavior.* 2018;8(4):e00949.
25. Wartberg L, Kriston L, Kammerl R. Associations of Social Support, Friends Only Known Through the Internet, and Health-Related Quality of Life with Internet Gaming Disorder in Adolescence. *Cyberpsychol Behav Soc Netw.* 2017a;20(7):436-441.
26. Wartberg L, Kriston L, Kramer M, Schwedler A, Lincoln TM, Kammerl R. Internet gaming disorder in early adolescence: Associations with parental and adolescent mental health. *Eur Psychiatry.* 2017b;43:14-18.
27. Wartberg L, Kriston L, Thomasius R. The Prevalence and Psychosocial Correlates of Internet Gaming Disorder. *Dtsch.* 2017c;114(25):419-424.
28. Wartberg L, Kriston L, Zieglmeier M, Lincoln T, Kammerl R. A longitudinal study on psychosocial causes and consequences of Internet gaming disorder in adolescence. *Psychol Med.* 2018:1-8.
29. Wichstrom L, Stenseng F, Belsky J, von Soest T, Hygen BW. Symptoms of Internet Gaming Disorder in Youth: Predictors and Comorbidity. *J Abnorm Child Psychol.* 2018;05:05.
30. De Pasquale C, Dinaro C, Sciacca F. Relationship of Internet gaming disorder with dissociative experience in Italian university students. *Ann Gen Psychiatry.* 2018;17:28.
31. Evren C, Dalbudak E, Topcu M, Kutlu N, Evren B. The psychometric properties of the Turkish version of the Internet Gaming Disorder Scale. *Dusunen Adam.* 2017;30(4):316-324.
32. Fuster H, Carbonell X, Pontes HM, Griffiths MD. Spanish validation of the Internet Gaming Disorder-20 (IGD-20) Test. *Comput Human Behav.* 2016;56:215-224.
33. Gunuc S. Relationships and associations between video game and Internet addictions: Is tolerance a symptom seen in all conditions. *Comput Human Behav.* 2015;49:517-525.
34. Laconi S, Pires S, Chabrol H. Internet gaming disorder, motives, game genres and psychopathology. *Comput Human Behav.* 2017;75:652-659.
35. Lopez-Fernandez O, Honrubia-Serrano M, Baguley T, Griffiths MD. Pathological video game playing in Spanish and British adolescents: Towards the exploration of Internet Gaming Disorder symptomatology. *Comput Human Behav.* 2014;41:304-312.
36. Milani L, La Torre G, Fiore M, Grumi S, Gentile DA, Ferrante M et al. Internet gaming addiction in adolescence: Risk factors and maladjustment correlates. *Int J Ment Health Addict.* 2018;16(4):888-904.

37. Palo V, Monacis L, Sinatra M, Griffiths MD, Pontes H, Petro M et al. Measurement invariance of the nine-item internet gaming disorder scale (IGDS9-SF) across Albania, USA, UK, and Italy. *Int J Ment Health Addict.* 2018:1-12.
38. Peeters M, Koning I, van den Eijnden R. Predicting Internet Gaming Disorder symptoms in young adolescents: A one-year follow-up study. *Comput Human Behav.* 2018;80:255-261.
39. Taquet P, Romo L, Cottencin O, Ortiz D, Hautekeete M. Video Game Addiction: Cognitive, emotional, and behavioral determinants for CBT treatment. *Journal de Therapie Comportementale et Cognitive.* 2017;27(3):118-128.
40. Tejeiro R, Espada J, Gonzalvez M, Christiansen P. Psychometric properties of the Problem Video Game Playing scale in adults. *Eur Rev Appl Psychol.* 2016;66(1):9-13.
41. Triberti S, Milani L, Villani D, Grumi S, Peracchia S, Curcio G et al. What matters is when you play: Investigating the relationship between online video games addiction and time spent playing over specific day phases. *Addict Behav Rep.* 2018;8:185-188.
42. Przybylski AK, Weinstein N, Murayama K. Internet Gaming Disorder: Investigating the Clinical Relevance of a New Phenomenon. *Am J Psychiatry.* 2017a;174(3):230-236.
43. Przybylski AK, Weinstein N, Murayama K. Internet Gaming Disorder: Investigating the Clinical Relevance of a New Phenomenon. *Am J Psychiatry*. 2017d;174(3):230-236.
44. Snodgrass JG, Dengah Ii HJF, Lacy MG, Else RJ, Polzer ER, Arevalo JMG et al. Social genomics of healthy and disordered internet gaming. *Am J Hum Biol.* 2018;30(5):e23146.
45. Stockdale L, Coyne SM. Video game addiction in emerging adulthood: Cross-sectional evidence of pathology in video game addicts as compared to matched healthy controls. *J Affect Disord.* 2018;225:265-272.
46. Stubblefield S, Datto G, Phan TT, Werk LN, Stackpole K, Siegel R et al. Problem Video Gaming Among Children Enrolled in Tertiary Weight Management Programs. *Cyberpsychol Behav Soc Netw.* 2017;20(2):109-116.
47. Weinstein N, Przybylski AK, Murayama K. A prospective study of the motivational and health dynamics of Internet Gaming Disorder. *PeerJ.* 2017;5:e3838.
48. Allen JJ, Anderson CA. Satisfaction and frustration of basic psychological needs in the real world and in video games predict internet gaming disorder scores and well-being. *Comput Human Behav.* 2018;84:220-229.
49. Bargeron AH, Hormes JM. Psychosocial correlates of internet gaming disorder: Psychopathology, life satisfaction, and impulsivity. *Comput Human Behav.* 2017;68:388-394.
50. Carlisle KL. Personality, motivation, and internet gaming disorder: Understanding the addiction. *Diss Abstr Int: Section B: The Sciences and Engineering.* 2018;78(11-B(E))
51. Li W. Mindfulness-Oriented Recovery Enhancement for video game addiction in U.S. emerging adults. *Diss Abstr Int Section A: Humanities and Social Sciences*. 2017a. 77(11-A(E)).
52. Sioni SR, Burleson MH, Bekerian DA. Internet gaming disorder: Social phobia and identifying with your virtual self. *Comput Human Behav.* 2017;71:11-15.
53. Jeong H, Yim HW, Lee SY, Lee HK, Potenza MN, Kwon JH et al. Discordance between self-report and clinical diagnosis of Internet gaming disorder in adolescents. *Sci Rep.* 2018;8(1):10084.
54. Jo SJ, Yim HW, Lee HK, Lee HC, Choi JS, Baek KY. The Internet Game Use-Elicited Symptom Screen proved to be a valid tool for adolescents aged 10-19 years. *Acta Paediatr.* 2018;107(3):511-516.
55. Kim NR, Hwang SS, Choi JS, Kim DJ, Demetrovics Z, Kiraly O et al. Characteristics and Psychiatric Symptoms of Internet Gaming Disorder among Adults Using Self-Reported DSM-5 Criteria. *Psychiatry Investig.* 2016b;13(1):58-66.

56. King DL, Delfabbro PH. The Cognitive Psychopathology of Internet Gaming Disorder in Adolescence. *J Abnorm Child Psychol.* 2016;44(8):1635-1645.
57. King DL, Herd MCE, Delfabbro PH. Tolerance in Internet gaming disorder: A need for increasing gaming time or something else? *J Behav Addict.* 2017a;6(4):525-533.
58. Lee SY, Lee HK, Jeong H, Yim HW, Bhang SY, Jo SJ et al. The Hierarchical Implications of Internet Gaming Disorder Criteria: Which Indicate more Severe Pathology? *Psychiatry Investig.* 2017b;14(3):249-259.
59. Na E, Choi I, Lee TH, Lee H, Rho MJ, Cho H et al. The influence of game genre on Internet gaming disorder. *J Behav Addict.* 2017a:1-8.
60. Na E, Lee H, Choi I, Kim DJ. Comorbidity of Internet gaming disorder and alcohol use disorder: A focus on clinical characteristics and gaming patterns. *Am J Addict.* 2017b;26(4):326-334.
61. Paik SH, Cho H, Chun JW, Jeong JE, Kim DJ. Gaming Device Usage Patterns Predict Internet Gaming Disorder: Comparison across Different Gaming Device Usage Patterns. *Int J Environ Res Public Health.* 2017a;14(12):05.
62. Pearcy BT, McEvoy PM, Roberts LD. Internet Gaming Disorder Explains Unique Variance in Psychological Distress and Disability After Controlling for Comorbid Depression, OCD, ADHD, and Anxiety. *Cyberpsychol Behav Soc Netw.* 2017;20(2):126-132.
63. Pearcy BT, Roberts LD, McEvoy PM. Psychometric Testing of the Personal Internet Gaming Disorder Evaluation-9: A New Measure Designed to Assess Internet Gaming Disorder. *Cyberpsychol Behav Soc Netw.* 2016;19(5):335-341.
64. Rao J, Wang H, Pang M, Yang J, Zhang J, Ye Y et al. Cyberbullying perpetration and victimisation among junior and senior high school students in Guangzhou, China. *Inj Prev.* 2017;06:06.
65. Rho MJ, Lee H, Lee TH, Cho H, Jung DJ, Kim DJ et al. Risk Factors for Internet Gaming Disorder: Psychological Factors and Internet Gaming Characteristics. *Int J Environ Res Public Health.* 2017;15(1):27.
66. Sigerson L, Li AY, Cheung MW, Luk JW, Cheng C. Psychometric properties of the Chinese Internet Gaming Disorder Scale. *Addict Behav.* 2017;74:20-26.
67. Subramaniam M, Chua BY, Abdin E, Pang S, Satghare P, Vaingankar JA et al. Prevalence and Correlates of Internet Gaming Problem among Internet Users: Results from an Internet Survey. *Ann Acad Med Singapore.* 2016;45(5):174-183.
68. Wang HR, Cho H, Kim DJ. Prevalence and correlates of comorbid depression in a nonclinical online sample with DSM-5 internet gaming disorder. *J Affect Disord.* 2018a;226:1-5.
69. Wu AM, Lai MH, Yu S, Lau JT, Lei MW. Motives for online gaming questionnaire: Its psychometric properties and correlation with Internet gaming disorder symptoms among Chinese people. *J Behav Addict.* 2017a;6(1):11-20.
70. Wu AMS, Chen JH, Tong KK, Yu S, Lau JTF. Prevalence and associated factors of Internet gaming disorder among community dwelling adults in Macao, China. *J Behav Addict.* 2018a;7(1):62-69.
71. Yu H, Cho J. Prevalence of Internet Gaming Disorder among Korean Adolescents and Associations with Non-psychotic Psychological Symptoms, and Physical Aggression. *Am J Health Behav.* 2016;40(6):705-716.
72. Yu S, Mao S, Wu AMS. The interplay among stress, frustration tolerance, mindfulness, and social support in Internet gaming disorder symptoms among Chinese working adults. *Asia Pac Psychiatry.* 2018;10(4):e12319.
73. Adams BL, Stavropoulos V, Burleigh TL, Liew LW, Beard CL, Griffiths MD. Internet gaming disorder behaviors in emergent adulthood: A pilot study examining the interplay between anxiety and family cohesion. *Int J Ment Health Addict.* 2018:1-17.

74. Burleigh TL, Stavropoulos V, Liew LW, Adams BL, Griffiths MD. Depression, internet gaming disorder, and the moderating effect of the gamer-avatar relationship: An exploratory longitudinal study. *Int J Ment Health Addict.* 2018;16(1):102-124.
75. Fu W, Yu CK-C. Predicting disordered gambling with illusory control, gaming preferences, and Internet gaming addiction among Chinese youth. *Int J Ment Health Addict.* 2015;13(3):391-401.
76. King DL, Delfabbro PH. Features of parent-child relationships in adolescents with Internet gaming disorder. *Int J Ment Health Addict.* 2017c;15(6):1270-1283.
77. Liew LW, Stavropoulos V, Adams BL, Burleigh TL, Griffiths MD. Internet gaming disorder: The interplay between physical activity and user-avatar relationship. *Behav Inf Technol.* 2018;37(6):558-574.
78. Schneider LA, King DL, Delfabbro PH. Maladaptive coping styles in adolescents with internet gaming disorder symptoms. *Int J Ment Health Addict.* 2018;16(4):905-916.
79. Pontes HM, Kiraly O, Demetrovics Z, Griffiths MD. The conceptualisation and measurement of DSM-5 Internet Gaming Disorder: the development of the IGD-20 Test. *PLoS One.* 2014;9(10):e110137.
80. Przybylski AK. Mischievous responding in Internet Gaming Disorder research. *PeerJ*. 2016b;4:e2401.
81. Przybylski AK, Weinstein N, Murayama K. Internet Gaming Disorder: Investigating the Clinical Relevance of a New Phenomenon. *Am J Psychiatry*. 2017c;174(3):230-236.
82. Pontes HM, Griffiths MD. Measuring DSM-5 Internet Gaming Disorder: Development and validation of a short psychometric scale. *Comput Human Behav.* 2015;45:137-143.
83. Krossbakken E, Torsheim T, Mentzoni RA, King DL, Bjorvatn B, Lorvik IM et al. The effectiveness of a parental guide for prevention of problematic video gaming in children: A public health randomized controlled intervention study. *J Behav Addict.* 2018;7(1):52-61.
84. Mallorquí-Bagué N, Fernandez-Aranda F, Lozano-Madrid M, Granero R, Mestre-Bach G, Bano M et al. Internet gaming disorder and online gambling disorder: Clinical and personality correlates. *J Behav Addict.* 2017;6(4):669-677.
85. Sanders J, Williams R, Damgaard M. Video Game Play and Internet Gaming Disorder Among Canadian Adults: A National Survey. *Can J Addict.* 2017;8(2):6-12.
86. Cai C, Yuan K, Yin J, Feng D, Bi Y, Li Y et al. Striatum morphometry is associated with cognitive control deficits and symptom severity in internet gaming disorder. *Brain Imaging Behav.* 2016;10(1):12-20.
87. Dong G, Wang L, Du X, Potenza MN. Gaming Increases Craving to Gaming-Related Stimuli in Individuals With Internet Gaming Disorder. *Biol Psychiatry Cogn Neurosci Neuroimaging.* 2017c;2(5):404-412.
88. Dong G WL, Wang Z, Wang Y, Du X, Potenza MN. Diffusion-weighted MRI measures suggest increased white-matter integrity in Internet gaming disorder: Evidence from the comparison with recreational Internet game users. *Addict Behav.* 2018a;81:32-38.
89. Kaptsis D, King DL, Delfabbro PH, Gradisar M. Trajectories of abstinence-induced Internet gaming withdrawal symptoms: A prospective pilot study. *Addict Behav Rep.* 2016;4:24-30.
90. Kim M, Lee TH, Choi JS, Kwak YB, Hwang WJ, Kim T et al. Neurophysiological correlates of altered response inhibition in internet gaming disorder and obsessive-compulsive disorder: Perspectives from impulsivity and compulsivity. *Sci Rep.* 2017a;7:41742.
91. Kim YJ, Lim JA, Lee JY, Oh S, Kim SN, Kim DJ et al. Impulsivity and compulsivity in Internet gaming disorder: A comparison with obsessive-compulsive disorder and alcohol use disorder. *J Behav Addict.* 2017c;6(4):545-553.

92. King DL, Adair C, Saunders JB, Delfabbro PH. Clinical predictors of gaming abstinence in help-seeking adult problematic gamers. *Psychiatry Res.* 2018a;261:581-588.
93. King DL, Kaptsis D, Delfabbro PH, Gradisar M. Effectiveness of Brief Abstinence for Modifying Problematic Internet Gaming Cognitions and Behaviors. *J Clin Psychol.* 2017b;73(12):1573-1585.
94. Ko CH, Wang PW, Liu TL, Chen CS, Yen CF, Yen JY. The adaptive decision-making, risky decision, and decision-making style of Internet gaming disorder. *Eur Psychiatry.* 2017;44:189-197.
95. Ko CH, Yen JY, Chen SH, Wang PW, Chen CS, Yen CF. Evaluation of the diagnostic criteria of Internet gaming disorder in the DSM-5 among young adults in Taiwan.[Erratum appears in J Psychiatr Res. 2014 Oct;57():185]. *J Psychiatr Res.* 2014;53:103-110.
96. Koo HJ, Han DH, Park SY, Kwon JH. The Structured Clinical Interview for DSM-5 Internet Gaming Disorder: Development and Validation for Diagnosing IGD in Adolescents. *Psychiatry Investig.* 2017;14(1):21-29.
97. Lee D, Lee J, Yoon KJ, Kee N, Jung YC. Impaired anterior insular activation during risky decision making in young adults with internet gaming disorder. *Neuroreport.* 2016;27(8):605-609.
98. Lee M, Cho H, Jung SH, Yim SH, Cho SM, Chun JW et al. Circulating MicroRNA Expression Levels Associated With Internet Gaming Disorder. *Front Psychiatry.* 2018c;9:81.
99. Li AY, Lo BC, Cheng C. It Is the Family Context That Matters: Concurrent and Predictive Effects of Aspects of Parent-Child Interaction on Video Gaming-Related Problems. *Cyberpsychol Behav Soc Netw.* 2018a;21(6):374-380.
100. Paik SH, Choi MR, Kwak SM, Bang SH, Chun JW, Kim JY et al. An association study of Taq1A ANKK1 and C957T and - 141C DRD2 polymorphisms in adults with internet gaming disorder: a pilot study. *Ann Gen Psychiatry.* 2017b;16:45.
101. Park M, Choi JS, Park SM, Lee JY, Jung HY, Sohn BK et al. Dysfunctional information processing during an auditory event-related potential task in individuals with Internet gaming disorder. *Transl Psychiatry.* 2016b;6:e721.
102. Rho MJ, Jeong JE, Chun JW, Cho H, Jung DJ, Choi IY et al. Predictors and patterns of problematic Internet game use using a decision tree model. *J Behav Addict.* 2016;5(3):500-509.
103. Ryu H, Lee JY, Choi A, Park S, Kim DJ, Choi JS. The Relationship between Impulsivity and Internet Gaming Disorder in Young Adults: Mediating Effects of Interpersonal Relationships and Depression. *Int J Environ Res Public Health.* 2018;15(3):06.
104. Wang CY, Wu YC, Su CH, Lin PC, Ko CH, Yen JY. Association between Internet gaming disorder and generalized anxiety disorder. *J Behav Addict.* 2017a;6(4):564-571.
105. Wang L, Wu L, Wang Y, Li H, Liu X, Du X et al. Altered Brain Activities Associated with Craving and Cue Reactivity in People with Internet Gaming Disorder: Evidence from the Comparison with Recreational Internet Game Users. *Front Psychol.* 2017b;8:1150.
106. Wang Z, Wu L, Yuan K, Hu Y, Zheng H, Du X et al. Cortical thickness and volume abnormalities in Internet gaming disorder: Evidence from comparison of recreational Internet game users. *Eur J Neurosci.* 2018c;08:08.
107. Yeh YC, Wang PW, Huang MF, Lin PC, Chen CS, Ko CH. The procrastination of Internet gaming disorder in young adults: The clinical severity. *Psychiatry Res.* 2017;254:258-262.
108. Yen JY, Liu TL, Wang PW, Chen CS, Yen CF, Ko CH. Association between Internet gaming disorder and adult attention deficit and hyperactivity disorder and their correlates: Impulsivity and hostility. *Addict Behav.* 2017a;64:308-313.

109. Yen JY, Yeh YC, Wang PW, Liu TL, Chen YY, Ko CH. Emotional Regulation in Young Adults with Internet Gaming Disorder. *Int J Environ Res Public Health.* 2017b;15(1):25.
110. Yuan K, Yu D, Cai C, Feng D, Li Y, Bi Y et al. Frontostriatal circuits, resting state functional connectivity and cognitive control in internet gaming disorder. *Addict Biol.* 2017;22(3):813-822.
111. Zhai J, Luo L, Qiu L, Kang Y, Liu B, Yu D et al. The topological organization of white matter network in internet gaming disorder individuals. *Brain Imaging Behav.* 2017;11(6):1769-1778.
112. King DL, Herd MC, Delfabbro PH. Motivational components of tolerance in internet gaming disorder. *Comput Human Behav.* 2018b;78:133-141.
113. Tian M, Tao R, Zheng Y, Zhang H, Yang G, Li Q et al. Internet gaming disorder in adolescents is linked to delay discounting but not probability discounting. *Comput Human Behav.* 2018;80:59-66.
114. Cho YU, Lee D, Lee JE, Kim KH, Lee DY, Jung YC. Exploratory metabolomics of biomarker identification for the internet gaming disorder in young Korean males. *J Chromatogr B Analyt Technol Biomed Life Sci.* 2017;1057:24-31.
115. Lee D, Hong SJ, Jung YC, Park J, Kim IY, Namkoong K. Altered Heart Rate Variability During Gaming in Internet Gaming Disorder. *Cyberpsychol Behav Soc Netw.* 2018a;21(4):259-267.
116. Kaess M, Parzer P, Mehl L, Weil L, Strittmatter E, Resch F et al. Stress vulnerability in male youth with Internet Gaming Disorder. *Psychoneuroendocrinology.* 2017;77:244-251.
117. van Rooij AJ, Schoenmakers TM, van de Mheen D. Clinical validation of the C-VAT 2.0 assessment tool for gaming disorder: A sensitivity analysis of the proposed DSM-5 criteria and the clinical characteristics of young patients with 'video game addiction'. *Addict Behav.* 2017;64:269-274.
118. Bae S, Han DH, Jung J, Nam KC, Renshaw PF. Comparison of brain connectivity between Internet gambling disorder and Internet gaming disorder: A preliminary study. *J Behav Addict.* 2017;6(4):505-515.
119. Choi J, Cho H, Kim JY, Jung DJ, Ahn KJ, Kang HB et al. Structural alterations in the prefrontal cortex mediate the relationship between Internet gaming disorder and depressed mood. *Sci Rep.* 2017;7(1):1245.
120. Choi SW, Kim HS, Kim GY, Jeon Y, Park SM, Lee JY et al. Similarities and differences among Internet gaming disorder, gambling disorder and alcohol use disorder: a focus on impulsivity and compulsivity. *J Behav Addict.* 2014;3(4):246-253.
121. Dong G, Li H, Wang L, Potenza MN. Cognitive control and reward/loss processing in Internet gaming disorder: Results from a comparison with recreational Internet game-users. *Eur Psychiatry.* 2017a;44:30-38.
122. Dong G, Li H, Wang L, Potenza MN. The correlation between mood states and functional connectivity within the default mode network can differentiate Internet gaming disorder from healthy controls. *Prog Neuropsychopharmacol Biol Psychiatry.* 2017b;77:185-193.
123. Dong G WZ, Wang Y, Du X, Potenza MN. Gender-related functional connectivity and craving during gaming and immediate abstinence during a mandatory break: Implications for development and progression of internet gaming disorder. *Prog Neuropsychopharmacol Biol Psychiatry.* 2018b;88:1-10.
124. Han JW, Han DH, Bolo N, Kim B, Kim BN, Renshaw PF. Differences in functional connectivity between alcohol dependence and internet gaming disorder. *Addict Behav.* 2015;41:12-19.

125. Jeong JE, Rhee JK, Kim TM, Kwak SM, Bang SH, Cho H et al. The association between the nicotinic acetylcholine receptor alpha4 subunit gene (CHRNA4) rs1044396 and Internet gaming disorder in Korean male adults. *PLoS One.* 2017;12(12):e0188358.
126. Kim H, Kim YK, Gwak AR, Lim JA, Lee JY, Jung HY et al. Resting-state regional homogeneity as a biological marker for patients with Internet gaming disorder: A comparison with patients with alcohol use disorder and healthy controls. *Prog Neuropsychopharmacol Biol Psychiatry.* 2015;60:104-111.
127. Kim JY, Jeong JE, Rhee JK, Cho H, Chun JW, Kim TM et al. Targeted exome sequencing for the identification of a protective variant against Internet gaming disorder at rs2229910 of neurotrophic tyrosine kinase receptor, type 3 (NTRK3): A pilot study. *J Behav Addict.* 2016a;5(4):631-638.
128. Lee D, Namkoong K, Lee J, Jung YC. Abnormal gray matter volume and impulsivity in young adults with Internet gaming disorder. *Addict Biol.* 2017a;08:08.
129. Lee D, Namkoong K, Lee J, Jung YC. Preliminary evidence of altered gray matter volume in subjects with internet gaming disorder: associations with history of childhood attention-deficit/hyperactivity disorder symptoms. *Brain Imaging Behav.* 2018b;11:11.
130. Lee J, Lee S, Chun JW, Cho H, Kim DJ, Jung YC. Compromised Prefrontal Cognitive Control Over Emotional Interference in Adolescents with Internet Gaming Disorder. *Cyberpsychol Behav Soc Netw.* 2015;18(11):661-668.
131. Park JH, Hong JS, Han DH, Min KJ, Lee YS, Kee BS et al. Comparison of QEEG Findings between Adolescents with Attention Deficit Hyperactivity Disorder (ADHD) without Comorbidity and ADHD Comorbid with Internet Gaming Disorder. *J Korean Med Sci.* 2017a;32(3):514-521.
132. Park M, Kim YJ, Kim DJ, Choi JS. Differential neurophysiological correlates of information processing in Internet gaming disorder and alcohol use disorder measured by event-related potentials. *Sci Rep.* 2017b;7(1):9062.
133. Park S, Ryu H, Lee JY, Choi A, Kim DJ, Kim SN et al. Longitudinal Changes in Neural Connectivity in Patients With Internet Gaming Disorder: A Resting-State EEG Coherence Study. *Front Psychiatry.* 2018;9:252.
134. Park SM, Lee JY, Kim YJ, Lee JY, Jung HY, Sohn BK et al. Neural connectivity in Internet gaming disorder and alcohol use disorder: A resting-state EEG coherence study. *Sci Rep.* 2017d;7(1):1333.
135. Sakuma H, Mihara S, Nakayama H, Miura K, Kitayuguchi T, Maezono M et al. Treatment with the Self-Discovery Camp (SDiC) improves Internet gaming disorder. *Addict Behav.* 2017;64:357-362.
136. Seok JW, Sohn JH. Altered Gray Matter Volume and Resting-State Connectivity in Individuals With Internet Gaming Disorder: A Voxel-Based Morphometry and Resting-State Functional Magnetic Resonance Imaging Study. *Front Psychiatry.* 2018;9:77.
137. Shin YB, Kim JJ, Kim MK, Kyeong S, Jung YH, Eom H et al. Development of an effective virtual environment in eliciting craving in adolescents and young adults with internet gaming disorder. *PLoS One.* 2018;13(4):e0195677.
138. Son KL, Choi JS, Lee J, Park SM, Lim JA, Lee JY et al. Neurophysiological features of Internet gaming disorder and alcohol use disorder: a resting-state EEG study. *Transl Psychiatry Psychiatry.* 2015;5:e628.
139. Wang L, Wu L, Lin X, Zhang Y, Zhou H, Du X et al. Altered brain functional networks in people with Internet gaming disorder: Evidence from resting-state fMRI. *Psychiatry Res Neuroimaging.* 2016a;254:156-163.
140. Wang L, Wu L, Lin X, Zhang Y, Zhou H, Du X et al. Dysfunctional default mode network and executive control network in people with Internet gaming disorder: Independent component analysis under a probability discounting task. *Eur Psychiatry.* 2016b;34:36-42.

141. Wang L, Zhang Y, Lin X, Zhou H, Du X, Dong G. Group independent component analysis reveals alternation of right executive control network in Internet gaming disorder. *Cns Spectrums.* 2017c:1-11.
142. Wang S, Liu J, Tian L, Chen L, Wang J, Tang Q et al. Increased Insular Cortical Thickness Associated With Symptom Severity in Male Youths With Internet Gaming Disorder: A Surface-Based Morphometric Study. *Front Psychiatry.* 2018b;9:99.
143. Wang Y, Hu Y, Xu J, Zhou H, Lin X, Du X et al. Dysfunctional Prefrontal Function Is Associated with Impulsivity in People with Internet Gaming Disorder during a Delay Discounting Task. *Front Psychiatry.* 2017d;8:287.
144. Wang Y, Wu L, Wang L, Zhang Y, Du X, Dong G. Impaired decision-making and impulse control in Internet gaming addicts: evidence from the comparison with recreational Internet game users. Addict Biol. 2017f;22(6):1610-1621.
145. Wang Y, Wu L, Zhou H, Lin X, Zhang Y, Du X et al. Impaired executive control and reward circuit in Internet gaming addicts under a delay discounting task: independent component analysis. *Eur Arch Psychiatry Clin Neurosci.* 2017e;267(3):245-255.
146. Wu Y, Sescousse G, Yu H, Clark L, Li H. Cognitive distortions and gambling near-misses in Internet Gaming Disorder: A preliminary study. *PLoS One.* 2018b;13(1):e0191110.
147. Yip SW, Gross JJ, Chawla M, Ma SS, Shi XH, Liu L et al. Is Neural Processing of Negative Stimuli Altered in Addiction Independent of Drug Effects? Findings From Drug-Naive Youth with Internet Gaming Disorder. *Neuropsychopharmacology.* 2018;43(6):1364-1372.
148. Youh J, Hong JS, Han DH, Chung US, Min KJ, Lee YS et al. Comparison of Electroencephalography (EEG) Coherence between Major Depressive Disorder (MDD) without Comorbidity and MDD Comorbid with Internet Gaming Disorder. *J Korean Med Sci.* 2017;32(7):1160-1165.
149. Zhang Y, Lin X, Zhou H, Xu J, Du X, Dong G. Brain Activity toward Gaming-Related Cues in Internet Gaming Disorder during an Addiction Stroop Task. *Front Psychol.* 2016;7:714.
150. Gonzalez-Bueso V, Santamaria JJ, Fernandez D, Merino L, Montero E, Jimenez-Murcia S et al. Internet Gaming Disorder in Adolescents: Personality, Psychopathology and Evaluation of a Psychological Intervention Combined With Parent Psychoeducation. *Front Psychol.* 2018;9:787.
151. Martin-Fernandez M, Matali JL, Garcia-Sanchez S, Pardo M, Lleras M, Castellano-Tejedor C. Adolescents with Internet Gaming Disorder (IGD): profiles and treatment response. *Adicciones.* 2017;29(2):125-133.
152. Li W, Garland EL, Howard MO. Therapeutic mechanisms of Mindfulness-Oriented Recovery Enhancement for internet gaming disorder: Reducing craving and addictive behavior by targeting cognitive processes. *J Addict Dis.* 2018b:1-9.
153. Li W, Garland EL, McGovern P, O'Brien JE, Tronnier C, Howard MO. Mindfulness-oriented recovery enhancement for internet gaming disorder in U.S. adults: A stage I randomized controlled trial. *Psychol Addict Behav.* 2017b;31(4):393-402.
154. Bae S, Hong JS, Kim SM, Han DH. Bupropion Shows Different Effects on Brain Functional Connectivity in Patients With Internet-Based Gambling Disorder and Internet Gaming Disorder. *Front Psychiatry.* 2018;9:130.
155. Kim YJ, Lee JY, Oh S, Park M, Jung HY, Sohn BK et al. Associations between prospective symptom changes and slow-wave activity in patients with Internet gaming disorder: A resting-state EEG study. *Medicine.* 2017b;96(8):e6178.
156. Lim JA, Lee JY, Jung HY, Sohn BK, Choi SW, Kim YJ et al. Changes of quality of life and cognitive function in individuals with Internet gaming disorder: A 6-month follow-up. *Medicine.* 2016;95(50):e5695.

157. Park JH, Lee YS, Sohn JH, Han DH. Effectiveness of atomoxetine and methylphenidate for problematic online gaming in adolescents with attention deficit hyperactivity disorder. *Hum Psychopharmacol.* 2016a;31(6):427-432.
158. Park M, Kim YJ, Kim DJ, Choi JS. Sustained dysfunctional information processing in patients with Internet gaming disorder: 6-month follow-up ERP study. *Medicine.* 2017c;96(36):e7995.
159. Song J, Park JH, Han DH, Roh S, Son JH, Choi TY et al. Comparative study of the effects of bupropion and escitalopram on Internet gaming disorder. *Psychiatry Clin Neurosci.* 2016;70(11):527-535.
160. Yao YW, Chen PR, Li CR, Hare TA, Li S, Zhang JT et al. Combined reality therapy and mindfulness meditation decrease intertemporal decisional impulsivity in young adults with Internet gaming disorder. *Comput Human Behav.* 2017;68:210-216.
